# Effectiveness of nordic walking in patients with asthma: A study protocol of a randomized controlled trial

**DOI:** 10.1371/journal.pone.0281007

**Published:** 2023-03-09

**Authors:** María Vilanova-Pereira, Cristina Jácome, Manuel Jorge Rial Prado, Margarita Barral-Fernández, Marina Blanco Aparicio, Lara Fontán García-Boente, Ana Lista-Paz

**Affiliations:** 1 The Faculty of Physiotherapy, The University of A Coruña, A Coruña, Galicia, Spain; 2 Center for Health Technology and Services Research (CINTESIS), Faculty of Medicine, University of Porto (FMUP), Porto, Portugal; 3 Department of Allergy, University Hospital of A Coruña, A Coruña, Galicia, Spain; 4 Department of Respiratory Medicine, University Hospital of A Coruña, A Coruña, Galicia, Spain; 5 Pulmonology Service, Hospital HM Modelo, A Coruña, Galicia, Spain; 6 Psychosocial and Functional Rehabilitation Intervention Research Group, The University of A Coruña, A Coruña, Galicia, Spain; Public Library of Science, UNITED KINGDOM

## Abstract

**Background:**

Patients with asthma often consider their symptomatology a barrier to exercise, leading to a reduced physical activity level. This study aims to determine whether the effect of a Nordic walking (NW) training program plus education and usual care is superior to educational and usual care only, in terms of exercise tolerance and other health-related outcomes in patients with asthma. The second aim is to explore the patients’ experience with the NW program.

**Methods:**

A randomized controlled trial will be conducted with 114 adults with asthma recruited in sanitary area of A Coruña, Spain. Participants will be randomized to NW or control groups in blocks of six and in the same proportion in each group. Participants in the NW group will enrol in supervised sessions during eight weeks, three times/week. All participants will receive three educational sessions on asthma self-management plus usual care (S1 Appendix). Outcomes such as exercise tolerance (primary outcome), physical activity level, asthma-related symptoms and asthma control, dyspnea, lung function, handgrip strength, health related quality of life, quality of sleep, treatment adherence and healthcare resources use will be measured pre and postintervention, and at three and six months of follow-up. Participants in the NW group will additionally participate in focus groups.

**Discussion:**

This is the first study analysing the effect of NW in patients with asthma. NW combined with education and usual care is expected to improve exercise tolerance, but also asthma-related outcomes. If this hypothesis is confirmed, a new community-based therapeutic approach will be available for patients with asthma.

**Trial registration:**

Study registered in ClinicalTrials.gov with number of register NCT05482620.

## Introduction

Asthma is one of the world´s largest non-communicable diseases [1], affecting 339 million people [2]. It is considered a major social and health concern, being associated with a high economic burden, related to days out of work and school, emergency department visits, hospitalizations and mortality [3, 4].

Asthma is characterized by a reversible airway obstruction presented with any combination of the following manifestations and symptoms: wheezing, dyspnea, cough, chest tightness, mucus hypersecretion and airway hyperresponsiveness [5]. Due to these symptoms, patients with asthma, in comparison to their peers, are characterized by lower levels of physical activity [6–10]. They also commonly exercise at lower intensities, mostly because they believe that their disease is a barrier to exercise [9, 11, 12]. In its turn, inactivity can lead to worsening in asthma symptoms and control, health-related quality of life (HRQoL) [13], and a decline in lung function, particularly in forced expiratory volume in one second (FEV_1_), in FEV_1_/forced vital capacity (FVC) ratio, and peak expiratory flow (PEF) [11, 14, 15].

According to the Global Initiative for Asthma (GINA) [16], adults with asthma should engage in physical activity and exercise programs for their overall health benefit. Nevertheless, recommendations on how to perform physical activity or exercise as a part of their treatment is missing in this guideline. There is some evidence showing a positive effect of exercise programs, such as walking, jogging or cycling, reducing exacerbations and rescue medication use [17], and improving asthma-related symptoms, asthma control, lung function [18], exercise tolerance, and HRQoL [19].

Nordic walking (NW) is one of the exercise programs that could have potential benefits for people with asthma. The main difference between NW and conventional walking is the use of two poles to boost the walk, involving for instance the upper limb during the march. It was born in Finland during the 1930s as a cross-country skiers training, and it was been developed, transitioning from skiers to university and educative fields, and then to literature and scientific publications [20, 21]. The evidence is still scarce, but it has shown benefits in exercise tolerance and HRQoL in other chronic conditions, such as metabolic syndrome, musculoskeletal disorders, Parkinson disease [22–24], cardiovascular diseases [25] and chronic obstructive pulmonary disease (COPD) [26]. In COPD, NW improved exercise tolerance (measured as meters walked in six-minute walking test-6MWT); daily physical activity levels; dyspnea; anxiety and HRQoL. COPD and asthma are both respiratory diseases that share common symptoms and signs, such as cough, dyspnea and limited air flow, as well as limited exercise tolerance. Therefore, we hypothesize that similar benefits with NW can also be achieved in patients with asthma.

Thus, the primary aim of this clinical trial is to determine whether the effect of a NW training program plus education and usual care is superior to education and usual care only, in terms of exercise tolerance and other health-related outcomes, in patients with asthma. Secondary, we aim to explore the patients’ experience with the NW program.

## Material and methods

### Study design

This is a two-arm, parallel, randomized controlled trial (RCT) to analyse the effects of an eight-week NW program plus education and usual care compared to education and usual care alone in patients with asthma (NCT05482620). Assessments will take place at baseline, after 8-weeks and then at three and six months of follow-up. This study will be conducted in A Coruña, Spain between June 2022 and June 2024. This protocol is reported according to the Standard Protocol Items: Recommendations for Interventional Trials (SPIRIT) guidelines (S1 Checklist) [27].

### Eligibility criteria

Subjects will be included if they have a clinical diagnosis of asthma, have at least 18 years old, have the desire of take part in the study, and the ability to understand and sign the informed consent. Exclusion criteria include: smokers, diagnosis of other respiratory disease; occurrence of an asthma exacerbation or respiratory tract infection in the last four weeks; acute myocardial infarction in the last six months; cardiac arrhythmia with a IIIb or superior grade in Lown scale; gait disorders due to neuro-muscle-skeletal system problems; comorbidities that implies reduced ability to exercise (e.g., severe anaemia, electrolyte imbalance, hyperthyroidism) [28, 29]. Subjects that are already engaging in exercise sessions of more than 30 minutes per day with a moderate or vigorous intensity; participated in a pulmonary rehabilitation program in the last three months [30], and are pregnant and lactating women will be excluded. In addition, subjects that present contraindications to perform cardiovascular exercise, following the American Heart Association [31] and to perform the 6MWT following the American Thoracic Society / European Respiratory Society (ATS/ERS) [32] criteria, will be excluded too.

### Sample size

To estimate the sample size needed, we used the tool created by the Clinic Epidemiologic and Biostatistics service from A Coruña University Hospital Complex (CHUAC) (http://bitly.ws/sDIp). This calculation was based on the 6MWT minimum clinically important difference in patients with asthma, which is set at 26 meters [33]. Using a standard deviation (SD) of 45.49 meters, obtained from a previous pilot study [34], and considering a power of 80%, an alpha level of 0.05, and a dropout rate of 15%, a total sample size of 114 is estimated (57 in each group).

### Recruitment

Subjects will be screened by physicians during consultations in the pulmonology and allergy services from the pulmonology and allergy services from A Coruña sanitary area and in primary care centres.

Subjects eligible in the study will be listed, and one researcher will be responsible to randomise blocks of six to either the NW group or control group (CG) in the same proportion. Randomization will be performed via a computer program for randomization (http://randomization.com/). The allocation will be made attending to the identification (ID) number, which will be then linked to the participants’ name in the list. The order will be unchangeable, once assigned.

### Blinding (masking)

The assignment to the groups will be made after the baseline evaluation. The physiotherapist in charge of the evaluation will be concealed from which group belongs each subject, and data obtained will be indexed in the database, using study ID numbers only to identify data. Due to the nature of the interventions, the physiotherapist in charge of conducting it, will not be blinded to the allocation.

### Intervention

Participants in both groups will attend three educational sessions together after the baseline assessment (S1 Appendix) (before the NW program starts for the experimental group). Participants will only know the result of the randomization after the education. Educational sessions will be conducted with groups of approximately 6–10 patients. Sessions will be facilitated by a physiotherapist, and will take place in the Faculty of Physiotherapy of The University of A Coruña. Different concepts and guidance related to asthma self-management will be addressed, based on an informative brochure built by the research team. This brochure will be provided to the participants at the end of the education component. If patients miss one session, they will be phone called to wonder about reasons, to be encouraged to assist next sessions and an individual education session will be scheduled to address the missed topic. Besides, patients should keep their usual care, namely attend their regular medical appointments, take the medication under prescription and follow physician recommendations as usual.

Participants in the NW group will additionally enrol in an eight-week NW program [35] of three sessions per week (total of 24 sessions). Feasibility of the NW program has previously been tested in four patients, who showed a good acceptance and satisfaction with intervention received [34]. Each session will last one hour and include: a warm-up period of 15 minutes with articular mobility, body-weight exercises with walking poles, and five minutes of walking without poles; 30 minutes of NW, with an intensity of 70–85% theoretical maximum heart rate (HRmax = 206.9 –(0.7 x age)) [36]; cold-down period of five minutes of relaxed walk without poles, stretching and breathing exercises. NW program will be provided by the same physiotherapist in charge of the educational component, who was trained in NW in Finland. When participants fail one exercise session, he/she will be called to wonder about reasons and to be encouraged to participate in the next session. After finishing the eight-week period, participants will be encouraged to continue by themselves the NW sessions and a pair of poles will be provided to achieve this objective.

### Outcomes

#### Primary outcome

The primary outcome will be exercise tolerance, measured by the distance walked during the 6MWT, a reliable and validated test in patients with asthma [37], which is strongly related with important clinical outcomes as dyspnea and fatigue perceived [32].

#### Secondary outcomes

Exercise tolerance will also be assessed through the number of repetitions during the one-minute sit-to-stand test (1MSTST). This test is focused in lower limb function, especially quadriceps force [38].

The secondary outcome measurements will include physical activity level (accelerometry, International Physical Activity Questionnaire -IPAQ, and patient’s diary), asthma-related symptoms and asthma control (Control of Allergic Rhinitis and Asthma Test -CARAT and patient’s diary), dyspnea (modified Medical Research Council -mMRC, and Borg scale), lung function (spirometry), handgrip strength (hand dynamometry), HRQoL (European Quality of Life Questionnaire– 5 dimensions– 5 levels -EQ-5D-5L, mini Asthma Quality of Life Questionnaire -miniAQLQ), quality of sleep (Pittsburgh Quality of Sleep Index -PQSI), treatment adherence (Test of Adhesion to Inhalers -TAI, and patient’s diary), and healthcare resources use (patient’s diary).

#### Other measures

Respiratory muscle strength (maximum inspiratory -MIP and expiratory pressure -MEP) will be assessed to characterize sample, since no change with NW program is expected. Correlation with other outcomes such as exercise tolerance and physical activity will be explored [39].

Adverse effects reported by the participants will be registered.

Qualitative data will be collected through focus groups to a better understanding of participants’ experience with NW, namely: experience and satisfaction with the intervention received, facilitators and barriers to their participation perceived improvement in asthma management and the way of afront their disease.

### Data collection methods

All the assessments will take place at the Faculty of Physiotherapy in The University of A Coruña. Assessments will be performed by the same trained physiotherapist (different from the one in charge of interventions), blinded to the group allocation. The evaluator has been trained to perform these assessments, in which she also has previous experience. To avoid the risk that participants reveal to which group they belong during the first evaluation, the allocation will be only made after all baseline measurements and the education component. The evaluator will be also blinded during the following assessment visits. In the case this blinding is compromised due to the possible comments of the participants, this will be reported. When a participant fails an evaluation appointment, the evaluator will contact him/her in order to propose a date to a new appointment.

After reading the information sheet and signing the informed consent, an anamnesis will be performed in order to collect the sociodemographic variables (age, sex, occupation…), past history (e.g., surgical, comorbidities, etc.) and to confirm the fulfilment of the eligibility criteria.

All questionnaires, excepting IPAQ, will be self-completed by the participants after a brief explanation of the evaluator. The evaluator will be available to clarify any doubts during the questionnaires filling and at the end will check if all questions were covered.

All outcomes will be measured at baseline, postintervention, and at three and six months follow-up, except respiratory muscle strength (measured only at baseline) and treatment adherence and healthcare resources use (not analysed at baseline). Adverse effects related to following described outcomes, or during intervention, will be registered if pertinent.

Exercise tolerance will be measured with 6MWT and 1MSTS. To perform the 6MWT, an indoor corridor 30 metres long will be used, following ATS/ERS guidelines [32]. Before and after the test, vital signs (heart rate, oxygen saturation and blood pressure), fatigue of the lower limbs and dyspnea (using the modified Borg scale 1–10) will be recorded [40]. Two tests will be performed, with a minimum rest of 30 minutes, and the longest distance of the two tests will be selected for the analysis. For the 1MSTST, an armless chair will be used to ensure the upper limbs are not taking part in action. Subjects will be instructed to perform as many sit-to-stand movements that they can do in one minute [41, 42].

Accelerometry and the IPAQ, short form will be used to assess physical activity. The accelerometer MoveMonitor DynaPort®MM+ (McRoberts, The Hague, the Netherlands) will be used to register physical activity during seven days. Time lying down, sitting, standing up and walking, number of steps per day, intensity of movement, kilocalories consumed and metabolic equivalent of task (MET), will be analysed. Validity of accelerometer has been documented in COPD [43] and this tool has been used in previous studies in patients with asthma [44]. Besides, we will use IPAQ-short form, administrated through personal interview. The questionnaire has been validated in the Spanish population [45] and results will be analysed following the indications of the IPAQ group [46]. Also, number of steps per day should be recorded by patients in their patient’s diary, registered by a Google Fit ® Smartphone App (Google LLC, Mountain View, CA, USA); and register of their physical activity or exercise performed on the non-session’s day and once the sessions period has finished.

Asthma-related symptoms and asthma control will be measured using the CARAT, validated in patients with asthma [47], which has been used in Spanish population [48]. Additional analysis will be made from data extracted from the patient´s diary, in terms of dyspnea measured with modified Borg scale, expectoration, cough, perception of wheezing, work absence due to asthma symptoms or avoidance of doing some activity due to same reason. Related to dyspnea, it will be assessed also during personal interview using the mMRC scale [49]. In addition, modified Borg scale will be used before and after each NW session [40].

Lung function will be assessed with a spirometry following international recommendations of ATS/ERS [50]. FEV_1_, FVC, FEV_1_/FVC, forced expiratory flow at 25 and 75% of the pulmonary volume (FEF_25-75%_) and PEF will be recorded, obtained with a spirometer (Datospir® 120C, Sibelmed, Barcelona, Spain). Moreover, PEF will be assessed every day by patients in their diary. A peak-flow meter will be provided to each patient by the study.

Handgrip strength will be measured in both hands, registering data in kilograms, and using Jamar® hand-dynamometer (Performance Health, Warrenville, IL, USA) and following Mathiowetz et al. protocol [51].

HRQoL will be assessed using a generic and asthma-specific questionnaires. EQ-5D-5L has been validated in patients with asthma [52] and in Spanish population [53]. MiniAQLQ has been validated to use in this population [54], and has already been used in Spanish context [55, 56].

Quality of sleep will be assessed using PQSI, validated in Spanish [57].

Related to treatment adherence, inhaler adherence will be evaluated using the TAI, validated in patients with asthma and in the Spanish population [58]. Also, participants should note in their diary when, what and in which dosage need take medicine in order to relieve symptoms.

Healthcare resources use will be analysed as well taking in count data from patient’s diary (emergency service or unscheduled medical visits due to asthmatic symptoms).

Respiratory muscle strength will be measured by MIP and MEP, following Spanish Society of Pneumology and Thoracic Surgery (SEPAR) recommendations [59]. A digital manometer MicroRPM® (Vyaire Medical GmbH, Hoechberg, Germany), a flanged mouthpiece and the PUMA® software (Vyaire Medical GmbH, Hoechberg, Germany) will be used. At least six acceptable measurements are required and three of them should be reproductible, with less than 5% of difference between them.

Qualitative data will be obtained through focus group meetings, moderated by an experienced researcher. Another researcher will take part in the meeting to observe and take notes. These two researchers, will start introducing themselves and then moderator will give a clear explanation of the aim of the meeting before starting. Then, following a semi-structured guide with open-ended questions (S2 Appendix), focus groups will be conducted in order to gather a better understanding of participants experience. Around 15 participants will participate in focus groups [60], after receiving the NW program. Every NW group will constitute a focus group. Focus groups will be conducted in a room of the Faculty of Physiotherapy, in the University of A Coruña, and are expected to last about 90 minutes. Focus groups will be recorded (video and audio) and transcribed under consent of participants.

Participants dropping out of the study, if any, will be contacted to record possible reasons, and in case they are from the NW group they will also asked regarding the possibility to participate in the focus group and to provide data registered in their diary.

#### Participant timeline

**Fig 1:**
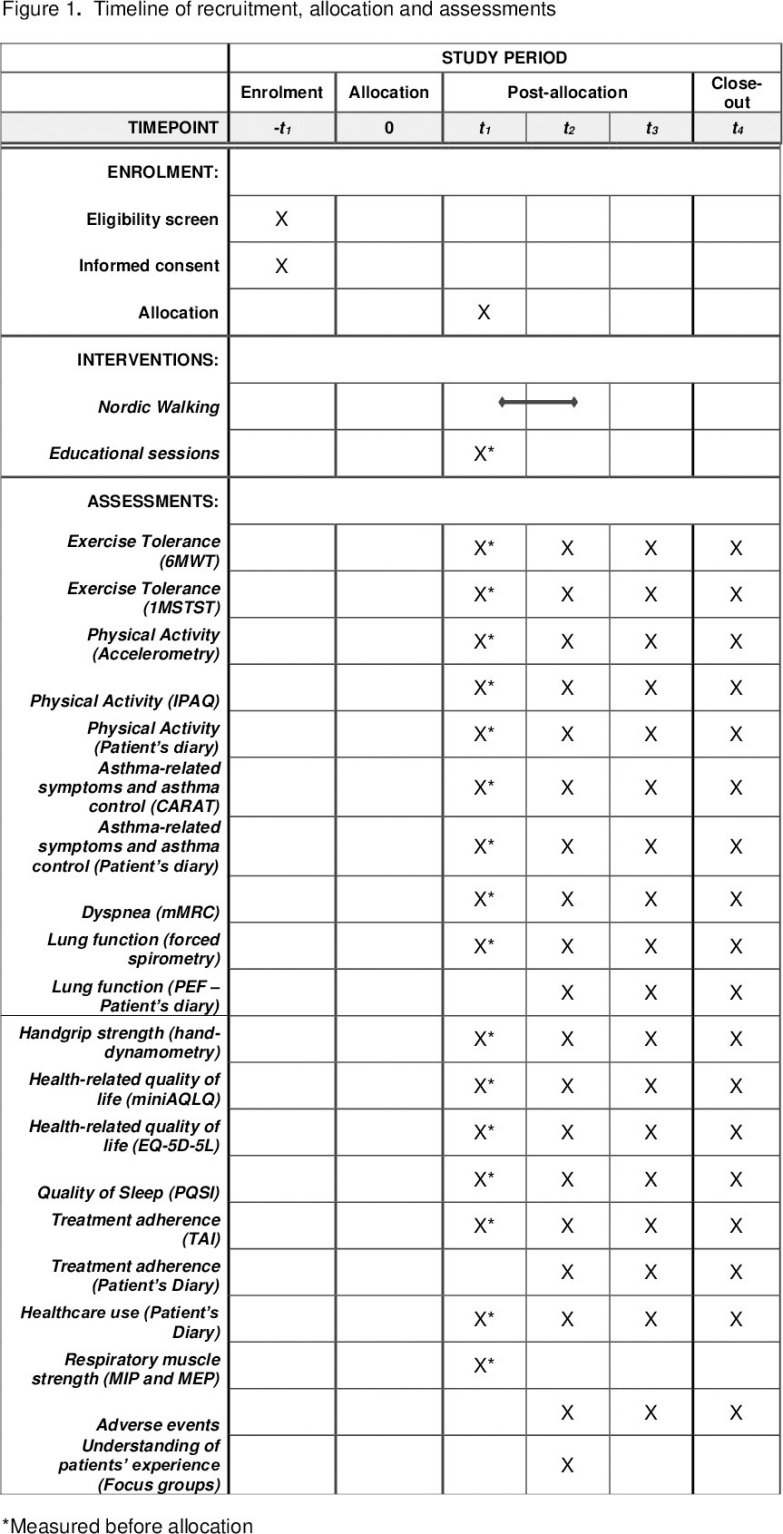
Timeline of recruitment, allocation and assessments. *Measured before allocation.

Participants will be assessed at baseline (T0), post-intervention (T1), and then at three (T2) and six months (T3) follow-up, as shown in Fig 1. After intervention, only NW group will be interviewed through focus groups.

### Data management and dissemination

Following what is established in Regulation (EU) 2016/679, it will be strictly respected the confidentiality of personal and health data of participants. Any data relative to participants will be stored coded with an alphanumeric ID.

All paper information will be stored in locked filing cabinet in the Faculty of Physiotherapy, The University of A Coruña. The principal researcher (ALP) and the physiotherapist in charge of intervention (MVP) of this study will be the only ones with access to this data.

Results of this study will be disseminated through scientific publications (original articles, abstracts) and communications in national and international congresses. Nonetheless, in none of the dissemination strategies, the identification of participants will be revealed.

Three of the authors (ALP, MVP and CJ) will compose the data monitoring committee.

### Ethics

This study has been approved by the Clinical Research Ethics Committee (CEIC) of Galicia (Spain) in July 20^th^, 2020 (code number 2019/574) (S3–S6 Appendices).

During the study, truthful and understandable information will be provided to the participants, referred to the objectives of this trial, to the procedures (during assessments and intervention), contraindications of them, and possible complications that can arise during it development (any inconvenient is expected, despite those referred from physical activity and regular healthcare practice). Both oral and written information will be provided, and informed consent will be obtained written.

Any important protocol modification will be communicated to CEIC. All changes will be registered as modifications in the clinical trial register database (www.clinicaltrials.gov) with register number NCT05482620.

### Statistical analysis

Quantitative data will be expressed as mean and SD when normally distributed and as median and quartiles (Q1-Q3) when not normally distributed. Normality will be explored with Kolmogorov-Smirnov tests. To compare outcomes between NW group and CG, ANOVA tests for repeated measures or Kruskal-Wallis tests will be used, as appropriate in function of normality of data distribution. All this data will be analysed using SPSS (IBM Corp., Armonk, NY, USA) program, version 26.00.

To analyse qualitative data, two of the researchers, following Braun et al. [61] recommendations, will deep read all transcripts and will make notes through text, to generate initial codes independently from each other. Then, the text will be re-read, searching and reviewing themes in relation to these codes, to be able to define and name the themes. After that, report can be produced. Another member of the team will check data to ensure credibility and trustworthy of findings, and through peer debriefing technique [62], researchers can be aware of their interpretation with the possibility to clean them of preconceptions or wrong assumptions. Agreement will be measured through agreement percentages, calculated as the number of thematic units in which evaluators agree divided by total number of units. Also kappa of Cohen will be used, considering >0.81 almost a perfect agreement [63].

## Results and discussion

This RCT aims to assess effectiveness of NW in patients with asthma. The study has been prospectively registered, and to our knowledge, it is the first study exploring this topic. Nordic walking is still a recent and novel exercise training modality, with few studies published in the health field. Based on previous studies about exercise programs [17–19], we hypothesise that the NW program in patients with asthma will increase exercise tolerance, physical activity level, asthma-related symptoms, asthma control, and HRQoL. Due to the characteristics of the intervention (involvement of the upper limbs), handgrip strength could also get improved [64]. We are expecting that lung function remains unchanged [65, 66], but as there is some controversy in current literature, we have decided to collect this data at all time-points [18, 19]. If the beneficial effects of NW are demonstrated, a new community-based therapeutic approach can be added to the current recommended and implemented in clinical practice. Nordic walking is affordable and feasible in the community, taking advantage of blue and green spaces in every patient´s environment–following Urban Training® concept–that have already shown benefits in terms of physical activity in patients with COPD [67]. Indirectly, the demonstration of our hypothesis could lead to added benefits, if the use of rescue medication, or medical visits (emergency department or unscheduled visits) are reduced: deriving from that reduction in healthcare resources use, a mitigation in socio-sanitary costs could be achieved.

### Strengthens and weaknesses

The NW program proposed in this RCT has been previously tested in a feasibility pilot study in patients with asthma. We found that NW is a well-tolerated and satisfactory activity, and people specially enjoy the possibility of make an activity group. Also, this pilot study allowed us to estimate the sample size needed [34]. Participants will be recruited during two years, and in consequence, NW program will be conducted across the seasons, bringing different challenges, such as exposure to environment allergens for patients with allergic asthma. This augmented exposure happens specially from January to June, in which pollen concentration are higher, and maybe during this period an attempt to schedule sessions in hours with lower pollen concentrations (hours with warm temperatures [68]) could be sought [69].

Due to the nature of the intervention, neither participants nor physiotherapist in charge are blinded to allocation. The possible bias can be minimized by the presence of a blinded evaluator. Nevertheless, after the intervention the evaluator´s blinding could be compromised due to possible comments of participants. If this happens, this will be reported.

Finally, some of the outcomes included in this study have already been analysed in patients with asthma after an exercise training programme and were shown to be sensitive to change [17–19, 26]. In addition, we will use both quantitative and qualitative methods, which can give a wider understanding of the feasibility of NW for patients, permitting analysing also the experience of participants with the exercise program. To our knowledge, this is the second study planning a focus group evaluation of NW training [70], and the third study approaching any qualitative study method, taking in account focus groups [71].

## Conclusion

Nordic Walking could be a new-community based therapeutic approach in patients with asthma, being affordable and feasible. Combined with educational and usual care, NW could improve exercises tolerance and other asthma related outcomes.

## Supporting information

S1 Checklist SPIRIT 2013 checklist: Recommended items to address in a clinical trial protocol and related documents*. (PDF)

S1 Appendix Index of educational sessions materials. (PDF)

S2 Appendix Semi-structured guide to focus groups. (PDF)

S3 Appendix Approval by ethics committee. Original document of approval of the clinical trial by Ethics Committee (in Spanish) (PDF)

S4 Appendix Translated approval by ethics committee. Translated document of approval of the clinical trial by the Ethics Committee (in English) (PDF)

S5 Appendix Original protocol sent to ethics committee. Original protocol sent to Ethics Committee (in Spanish). (PDF)

S6 Appendix Translate to English of protocol sent to ethics committee. Translated protocol sent Ethics Committee (in English). (PDF)
