# Supplementary material for: Effectiveness of nordic walking in patients with asthma: A study protocol of a randomized controlled trial
Source: PLoS One. 2023 Mar 9;18(3):e0281007. doi: 10.1371/journal.pone.0281007 (PMC9997906; doi:10.1371/journal.pone.0281007)
Supplement: S1 Appendix — (PDF) [file pone.0281007.s002.pdf]

Appendix 1. Index of Educational Sessions materials

- Anatomy of respiratory system.
    - Extrapulmonary airways.
    - Intrapulmonary airways.
- Control system breathing.
- What is asthma?
    - How many people are suffering from asthma? Prevalence of asthma.
    - What are the risk factors of asthma?
    - What causes asthma? Pathogeny.
- How is doing asthma diagnosis?
- Classification of asthma.
    - Classification related to severity.
    - Classification related to asthma control.
- Asthmatic exacerbation.
- Asthma treatment.
    - Maintenance treatment.
    - Rescue treatment.
- Correct use of inhalers (practical demonstrations).
    - Types of inhalers.
    - Pressurized metered dose inhaler (pMDI)
    - Spacers and inhalation chamber.
    - Dry-powder inhalers.
- What is peak expiratory flow? Practical demonstration of its assessment
- Asthma trigger factors.
- How to control environment as asthma treatment.
- Recommendations for a healthy lifestyle.

- Physical activity recommendations.
- Nutritional recommendations.

- Breathing control as asthma control tool.
- How to act in case of asthma emergency? Action plan.
