# Supplementary material for: Effectiveness of nordic walking in patients with asthma: A study protocol of a randomized controlled trial
Source: PLoS One. 2023 Mar 9;18(3):e0281007. doi: 10.1371/journal.pone.0281007 (PMC9997906; doi:10.1371/journal.pone.0281007)
Supplement: S2 Appendix — (PDF) [file pone.0281007.s003.pdf]

Appendix 2. Semi-structured guide to focus groups.

- What have changed in your way to live with asthma after realize this intervention?
- Would you recommend to other people with asthma to try Nordic walking? Why?
- Can you point some positive aspects do you find in its realization?
- What negative aspect do you find in this intervention (Nordic walking)?
- What would you change, if possible, in the sessions/intervention?
