# Supplementary material for: Effectiveness of nordic walking in patients with asthma: A study protocol of a randomized controlled trial
Source: PLoS One. 2023 Mar 9;18(3):e0281007. doi: 10.1371/journal.pone.0281007 (PMC9997906; doi:10.1371/journal.pone.0281007)
Supplement: S3 Appendix — (PDF) [file pone.0281007.s004.pdf]

DICTAMEN DEL COMITÉ DE ÉTICA DE LA INVESTIGACIÓN DE A CORUÑA - FERROL

Natalia Cal Purriños, Secretaria del Comité de Ética de la Investigación de A Coruña-Ferrol

CERTIFICA:

Que este Comité evaluó en su reunión del día 25/04/22 la modificación del estudio**:**

> **Título:** Comparación de un programa de marcha nórdica frente al uso exclusivo de un plan educacional en pacientes con asma
> **Versión modificación**: *modificación Febrero 2022*
> **Promotor/a**:*Universidade da Coruña. Ministerio de Educación Cultura y Deporte.*
> **Investigador/a**: María Vilanova Pereira
> **Código de Registro**: 2019/574

Y que este Comité acepta de conformidad con sus procedimientos normalizados de trabajo y tomando en cuenta los requisitos éticos, metodológicos y legales exigibles a los estudios de investigación con seres humanos, sus muestras o registros, que dicha modificación sea incorporada al estudio de investigación mencionado.

Documento asinado dixitalmente por:
Natalia Cal Purriños (10/05/2022 09:42)
https://sede.xunta.gal/cve?idcve=SAOC-K4G4-BOAH-OF4O-TL5K-9XZC-A7Y2-S165-2168-5709-86

Y HACE CONSTAR QUE:

1. El Comité Territorial de Ética de la Investigación de A Coruña-Ferrol cumple los requisitos legales vigentes

2. La composición actual del Comité Territorial de Ética de la Investigación de A Coruña-Ferrol es:

Carmen Mella Pérez. (Presidenta). Médica especialista en Medicina Interna. Área Sanitaria Ferrol.
Belén López Viñas. (Vicepresidenta). Médica especialista en Obstetricia y Ginecología. Área Sanitaria A Coruña y Cee.
Natalia Cal Purriños. (Secretaria). Licenciada en Derecho. Delegada de Protección de Datos. Fundación "Profesor Novoa Santos", A Coruña.
Sonia Pértega Díaz. (Vicesecretaria). Matemática. Área Sanitaria A Coruña y Cee.
Francisco Javier Afonso Afonso. Médico especialista en Oncología. Área Sanitaria Ferrol.
Juana Mª Cruz del Rio. Trabajadora social. Consellería de Sanidad.
María Ángeles Freire Fojo. Farmacéutica. Especialista en Farmacia Hospitalaria. Área Sanitaria Ferrol.
Salvador García Ruise. Médico especialista en Medicina Preventiva. Área Sanitaria Ferrol.
Portal González Lorenzo. Médica especialista en Medicina Familiar y Comunitaria. Área Sanitaria Ferrol.
Daniel Llamas Gómez. Ingeniero Informático. Experto en RGPD. Área Sanitaria A Coruña y Cee.
Isaac Martínez Bendayán. Médico especialista en Cardiología. Licenciado en Derecho. Área Sanitaria Ferrol.
Gonzalo Peña Pérez. Médico especialista en Cardiología. Hospital de San Rafael, A Coruña.
Carlos Rodríguez Moreno. Médico especialista en Farmacología Clínica. Área Sanitaria Santiago de Compostela y Barbanza.
José Mª Rumbo Prieto. Diplomado en Enfermería. Área Sanitaria Ferrol.

Para que conste donde proceda, y a petición de quien corresponda, en A Coruña.

La Secretaria del Comité Territorial de Ética de la Investigación de A Coruña – Ferrol,

Natalia Cal Purriños

Documento asinado dixitalmente por:
Natalia Cal Purriños (10/05/2022 09:42)
https://sede.xunta.gal/cve?idcve=SAOC-K4G4-BOAH-OF4O-TL5K-9XZC-A7Y2-S165-2168-5709-86
