# Supplementary material for: Effectiveness of nordic walking in patients with asthma: A study protocol of a randomized controlled trial
Source: PLoS One. 2023 Mar 9;18(3):e0281007. doi: 10.1371/journal.pone.0281007 (PMC9997906; doi:10.1371/journal.pone.0281007)
Supplement: S4 Appendix — (PDF) [file pone.0281007.s005.pdf]

TRADUCTION OF ORIGINAL LETTER OF APPROVAL

Dictum of Clinical Research Ethics Committee (CEIC) of A Coruña - Ferrol

Natalia Cal Purriños, Secretary of Clinical Research Ethics Committee (CEIC) of A Coruña-Ferrol

CERTIFIES:

This Committee evaluated in their meeting of 20th of July, 2020 the study:

> **Title:** Comparison of a Nordic walking program versus the exclusive use of an educational plan in patients with asthma.
>
> **Version:**
>
> **Promotor:** University of A Coruña. Education, Culture and Sports Ministers.
>
> **Researcher:** María Vilanova Periera
>
> **Register code:** 2019/575

And this Committee, considering the ownership of the study, the available knowledge, the ethics, methodologic and legal requirements for researcher studies related to human beings, their samples or registers, and the Normalized Procedures of Work of this Committee, emits a **FAVORABLE*** dictum for the realization of this study.

***NOTE:**

It is needed the modification in the document of Informed Consent, in section "¿Why am I offered to participate?". The paragraph: "You are invited to participate because you appear in the lists of Allergology area of University and Hospitalary Complex of A Coruña and HM Modelo Hospital from A Coruña, and furthermore, you accomplish the inclusion criteria of this study. Only the text "You are invited to participate because you are under 18 years old and you have a diagnosis of asthma. Moreover, you have the desire to participate in this study, and have the ability to sign the informed consent. For this reasons, you specialist physician have propose you in medical appointment to engage this project" should be maintained.

AND MAKES NOTICES THAT:

1. Clinical Research Ethics Committee (CEIC) of A Coruña – Ferrol accomplish the legal requirments in force.
2. The currently composition of Clinical Research Ethics Committee (CEIC) of A Coruña – Ferrol are:

Carmen Mella Pérez (President). Specialist Physician in Internal Medicine. Area of Integrated Gestion of Ferrol.
Angel Lopez-Silvarrey Varela. (Vice-president). Specialist Physician in Paediatrics. Area of Integrated Gestion of A Coruña.
Natalia Cal Purriños. (Secretary). Graduated in laws. Fundation "Profesor Novoa Santos". A Coruña.
Sonia Pértega Díaz. (Vice-secretary). Mathematician. Area of Integrated Gestion of A Coruña.

Juana Mª Cruz del Rio. Social work. Consellería de Sanidad.
María Ángeles Freire Fojo. Pharmacist. Specialist in Hospitalary Pharmacy. Area of Integrated Gestion of Ferrol.
Portal González Lorenzo. Specialist Physician of Family and Community Medicine. Area of Integrated Gestion of Ferrol.
Isaac Martínez Bendayán. Specialist Physician of Cardiology. Area of Integrated Gestion of A Coruña.
María Otero Santiago. Médica especialista en Medicina Preventiva y Salud Pública. Area of Integrated Gestion of A Coruña.
Alejandro Pazos Sierra. Physician. University of A Coruña
Gonzalo Peña Pérez. Specialist Physician of Cardiology. Area of Integrated Gestion of A Coruña.
Carlos Rodríguez Moreno. Specialist Physician of Clinical Pharmacology. Area of Integrated Gestion of Santiago.
José Mª Rumbo Prieto. Graduated in Nursing. Area of Integrated Gestion of Ferrol.
María Isabel Sastre Gervás. Pharmacist of Primary Attention. Area of Integrated Gestion of A Coruña.

For the record where appropriate, and at the request of the appropriate party, in A Coruña.

The Secretary of the Regional Research Ethics Committee of A Coruña – Ferrol,

Natalia Cal Purriños
