# Supplementary material for: Effectiveness of nordic walking in patients with asthma: A study protocol of a randomized controlled trial
Source: PLoS One. 2023 Mar 9;18(3):e0281007. doi: 10.1371/journal.pone.0281007 (PMC9997906; doi:10.1371/journal.pone.0281007)
Supplement: S5 Appendix — (PDF) [file pone.0281007.s006.pdf]

COMPARACIÓN DE UN PROGRAMA DE MARCHA NÓRDICA

FRENTE AL USO EXCLUSIVO DE UN PLAN EDUCACIONAL

EN PACIENTES CON ASMA

Investigadora principal

Dña. María Vilanova Pereira

Investigadores colaboradores

Dra. Ana Lista Paz

Dña. Margarita Barral Fernández

Dr. Alejandro Quintela del Río

Dr. Larissa Patricia Fontán García Boente

Dra. Marina Blanco Aparicio

Dr. Manuel Jorge Rial Prado

Tabla de contenido

Tabla de contenido II
Índice de acrónimos y abreviaturas VI
1. Resumen 1
1. Resumo 3
2. Contextualización 4
2.1 Antecedentes 4
3.1.1 Marcha nórdica 4
2.1.2 Asma y Fisioterapia 13
2.2 Justificación del trabajo 16
3. Hipótesis y objetivos 16
3.1 Hipótesis: nula y alternativa 16
3.2 Pregunta de investigación 16
3.3 Objetivos: generales y específicos 17
4. Metodología 17
4.1 Ámbito de estudio 17
4.2 Período de estudio 18
4.3 Tipo de trabajo 18
4.4 Criterios de selección 18
Criterios de inclusión: 18
Criterios de exclusión: 18
4.5 Justificación del tamaño muestral 19
4.6 Selección de la muestra 20
4.7 Descripción de las variables 20
4.7.1 Tolerancia al ejercicio 20
4.7.2 Nivel de actividad física diaria 20
4.7.3 Calidad de vida 20
4.7.4 Control del asma y síntomas 21
4.7.5 Disnea 21

4.7.6 Función pulmonar 21
4.7.7 Fuerza musculatura respiratoria 22
4.7.8 Fuerza-resistencia de la musculatura 22
4.7.9 Toma de medicamentos 22
4.7.10 Visitas a urgencias 22
4.7.11 Adherencia 22
4.7.12. Análisis cualitativo 23
4.8 Mediciones e intervención 23
4.8.1 Mediciones 23
4.8.2 Intervención 27
4.9 Análisis estadístico 31
5. Cronograma y etapas del desarrollo 32
5.1. Diseño del proyecto 32
5.2 Comité de ética. 32
5.3 Selección de la muestra 32
5.4 Desarrollo de la investigación 33
5.5 Análisis de los resultados 33
5.6 Difusión de los resultados. 33
6. Aspectos éticos-legales 33
7. Aplicabilidad del estudio 34
8. Plan de difusión de los resultados 36
8.1 Congresos 36
8.2 Revistas 36
9. Memoria económica 37
9.1 Recursos necesarios 37
9.2 Posibles fuentes de financiación 37
10. Medios disponibles y equipo investigador para la realización de la propuesta 38
10.1 Experiencia del equipo investigador 38
10.2 Recursos materiales disponibles 39

11. Bibliografía.......... 41

12. Anexos.......... 50

Anexo 1: Contraindicaciones para la participación en el estudio.......... 50

Anexo 2. Diario del paciente.......... 52

Anexo 3. Hoja de información del paciente (castellano y gallego).......... 53

Anexo 5. Cuaderno de recogida de datos del paciente.......... 67

Anexo 6. Registro del test de 6 minutos marcha.......... 73

Anexo 7. Cuestionario Internacional de Actividad Física. Versión corta.......... 74

Anexo 8. Cuestionario de calidad de vida *Short Form- Health Survey* 36.......... 76

Anexo 9. *Asthma Quality of Life Questionnaire*.......... 80

Anexo 10. *Asthma Control Test*.......... 82

Anexo 11. Test de Adhesión a los Inhaladores (TAI).......... 84

Anexo 12. Escala modificada de la *Medical Research Council*.......... 85

Anexo 13. Escala de Borg modificada.......... 86

Anexo 14. Plan educacional para los pacientes.......... 87

Anexo 15. Guion método cualitativo.......... 99

Anexo 16. Guía del paciente.......... 100

Índice de tablas

Tabla 1. Longitud del bastón de marcha nórdica en función de la altura de la persona. 5
Tabla 2. Clasifiación del asma en función de los síntomas. 14
Tabla 3 . Distancia mínima clínicamente significativa del SF-36 clasificado por secciones. 21
Tabla 4. Variables de estudio e instrumento de medida. 23
Tabla 5. Plan educacional para los pacientes 28
Tabla 6. Plan de entrenamiento. 29
Tabla 7. Cronograma y plan de trabajo 31
Tabla 8. Material necesario. 36

Índice de acrónimos y abreviaturas

**6MWT**	Test de 6 Minutos Marcha
**ACV**	Accidente cerebrovascular
**ATS/ERS**	*American Thoracic Society / European Respiratory Society*
**ACT**	*Asthma Control Test*
**AQLQ**	*Asthma Quality Of Life Questionnaire*
**COFIGA**	Colexio Oficial De Fisioterapeutas De Galicia
**CHUAC**	Complejo Hospitalario Universitario A Coruña
**VO_2**	Consumo de oxígeno
**VO_{2pico}**	Consumo de oxígeno pico
**$VO_{2máx}$**	Consumo de oxígeno máximo
**MCID**	Diferencia mínima clinicamente significativa
**EAP**	Enfermedad arterial periferica
**EPOC**	Enfermedad pulmonar obstructiva crónica
**MET**	Equivalentes metabólicos
**mMRC**	Escala Modificada del *Medical Research Council*
**FEV_1**	Flujo espiratorio máximo en el primer segundo
**FC**	Frecuencia cardíaca
**FCmáx**	Frecuencia cardíaca máxima
**FVC**	Capacidad vital forzada
**IMC**	Índice de masa corporal
**IP**	Investigadora principal
**IPAQ**	*Internacional Physical Activity Questionaire*
**MRC**	*Medical research council*
**PEF**	Pico expiratorio flujo
**PEM**	Presión expiratoria máxima
**PIM**	Presión inspiratoria máxima
**SF-36**	*Short Form Health Survey*
**SATSE**	Sindicato De Enfermería
**SEPAR**	Sociedad Española De Neumología Y Cirugía Torácica
**TAI**	Test de Adhesión a los Inhaladores

1. Resumen

Introducción

La marcha nórdica es un tipo de marcha que se diferencia de la tradicional principalmente por el uso de dos bastones especialmente diseñados para ello que se usan para impulsarse. Ha demostrado múltiples beneficios con respecto a la marcha tradicional, al igual que beneficios en multiples patologías cardiovasculares, músculo-esqueléticas y respiratorias. Hasta el momento, no ha sido estudiada en relación al asma.

Objetivo

El objetivo de este estudio es analizar si la marcha nórdica presenta, con respecto a un plan educacional, beneficios en pacientes asmáticos.

Metodología

Para responder a la pregunta de investigación, se ha diseñado un ensayo clínico aleatorizado simple ciego, con pacientes reclutados por los médicos de neumología y alergología del Complejo Hospitalario Universitario de A Coruña (CHUAC) y de neumología del Hospital HM Modelo de A Coruña. Este programa será combinado con un plan educacional en el grupo de estudio, mientras que el grupo control será solo participe del plan educacional. El programa durará 8 semanas y constará de 3 sesiones semanales en la que los participantes realizarán 30 minutos de marcha nórdica a una intensidad incremental a lo largo de la sesiones, empezando al 50% y subiendo 5% por semana, hasta alcanzar el 75% de la frecuencia cardíaca máxima teórica. Se analizarán posibles diferencias en la tolerancia al ejercicio, nivel de actividad física, calidad de vida, control de asma y síntomas, disnea, función pulmonar, fuerza-resistencia de la musculatura, sibilancias, toma de medicamentos, visitas a urgencias y adherencia al tratamiento. Se obtendrá información cualitativa a través de una entrevista semi estruturada en grupo mediante grupos focales. Los datos cualitativos recabados se analizarán mediante análisis del contenido temático.

Palabras clave:

Asma; Ejercicio; Marcha; Fisioterapia; Tolerancia Al Ejercicio.

1.Abstract

Introduction

Nordic walking is a type of walk that differs from the traditional one mainly by the use of two sticks specially designed for it, that are used to propolse. It has shown multiple benefits with respect to the traditional walk, as well as benefits in multiple cardiovascular, musculoskeletal and respiratory pathologies. So far, it has not been studied in relation to asthma

Objective

The main objective in this study is to analyse if Nordic walking has, compared with conventional cares and recommendations, benefits in asthmatic patients.

Methodology

To answer the research question, a simple blind randomized clinical trial, with patients recruited by the doctors of pulmonology and allergology of the A Coruña University Hospitalary Complex (CHUAC) and pulmonology of the Hospital HM Modelo of A Coruña. This program will be combined with an educational plan in the study group, while the control group will only participate in the recommendations and advice on conventional care. The program will last 8 weeks and will consist of 3 weekly sessions in which the participants will perform 30 minutes of Nordic walking at an incremental intensity throughout the sessions, starting at 50% and increasing 5% per week, until reaching 75% of the theoretical maximum heart rate. Possible differences in exercise tolerance, physical activity level, quality of life, asthma control and symptoms, dyspnoea, pulmonary function, strength-endurance of the musculature, wheezing, taking medications, visits to the emergency system and adherence to treatment will be analysed. Qualitative data will be achieved through focus groups meetings, with semi-structured interviews guides, and qualitative data will be analysed through thematic qualitative analysis.

Keywords

Asthma; Exercise; Walking; Physical Therapy, Modalities; Exercise Tolerance.

1. Resumo

Introdución:

A marcha nórdica é un tipo de marcha que se diferencia da tradicional principalmente polo uso de dous bastóns especialmente deseñados, que se usan para impulsarse. Demostrou múltiples beneficios con respecto á marcha tradicional, ao igual que beneficios en múltiples patoloxías cardiovasculares, músculo-esqueléticas e respiratorias. Ata o momento, non foi estudada en relación ao asma.

Obxectivos:

O obxectivo deste estudio é analizar se a marcha nórdica presenta, con respecto aos coidados e recomendacións convencionais, beneficios en pacientes asmáticos.

Metodoloxía:

Para responder á pregunta de investigación, deseñouse un ensaio clínico aleatorizado simple cego, con pacientes reclutados por los médicos de neumoloxía e arlegoloxía do Complejo Hospitalario Universitario de A Coruña (CHUAC) e neuomoloxía do Hospital HM Modelo de A Coruña. Este programa será combinado cun plan educacional no grupo de estudo, mentres que o grupo control será só participe das recomendacións e consellos sobre os coidados convencionais. O programa durará 8 semanas, constará de 3 sesións semanais nas que os participantes realizarán 30 minutos de marcha nórdica a unha intensidade incremental ao largo das sesións, empezando ao 50% e subindo 5% por semana, ata alcanzar o 75% da frecuencia cardíaca máxima teórica. Analizaranse posibles diferenzas na tolerancia ao exercicio, nivel de actividade física, calidade de vida, control do asma e síntomas, disnea, función pulmonar, forza-resistencia da musculatura, sibilancias, toma de medicamentos, visitas a urxencias e aderenza ao tratamento. Obterase información cualitativa a través dunha entrevista semi estructurada en grupo mediante grupos focales. Os datos cualitativos recabados analizaranse mediante análise de contido temático.

Palabras chave:

Asma; Marcha; Ejercicio; Fisioterapia; Tolerancia Ao Exercicio.

2. Contextualización

Este epígrafe tiene como objetivo explicar al lector qué es y cómo surge la marcha nórdica entrando en detalle a continuación en sus múltiples beneficios fisiológicos y biomecánicos, así como en sus beneficios en diferentes patologías y condiciones, centrándonos especialmente en las patologías cardiovasculares y respiratorias, hacia las cuales está orientado este proyecto.

Por último, se profundiza en la patología asmática, que es la finalmente elegida para la realización de la investigación, por no existir hasta la fecha de redacción de este manuscrito, ninguna investigación científica que relacione ambas: marcha nórdica y asma. Además, se explica la última evidencia disponible sobre el abordaje del asma desde la Fisioterapia, antes de presentar el proyecto de investigación.

2.1 Antecedentes

3.1.1 Marcha nórdica

La marcha nórdica es un tipo de actividad física, que se sirve de dos bastones especialmente diseñados para ello, que impulsan la marcha tradicional, respetando la biomecánica y postura natural de ésta (1). Hasta la fecha ha sido estudiada en relación a sus beneficios en múltiples patologías, entre ellas enfermedades reumáticas, neurológicas, como Parkinson, cardiovasculares o respiratorias, entre otras (2–4).

2.1.1.1 Historia

No está claro cuál es el origen de la marcha nórdica. La *World Record – Nordic Walking*, la data en los años 30, naciendo como entrenamiento de verano para esquiadores de fondo, pudiendo ser realizado en caminos de tierra o en asfalto (5).

Sin embargo, la *International Nordic Walking Asociation* sitúa el nacimiento de esta nueva forma de marcha en el año 1966, cuando Leena Jääskeläinem introduce los bastones como complemento para la marcha en una clase de educación física. Consciente de que la marcha nórdica aumentaba los beneficios de la caminata tradicional, continuó recomendando los bastones como una herramienta más de la marcha, en sus clases en la universidad (6).

El primero en escribir sobre el concepto fue Mauri Repo en la publicación *“Hiihdon Lajiosa” (TUL, 1979)*, traducido*: A part of cross-country skiing training methodic* (5). Un texto que fue utilizado para la promoción de la marcha nórdica (6), por lo que se considera el fundador de la técnica (5).

En los 90 llega a la población general como una actividad recreativa, gracias a Tuomo Jantunen, director de *Suomen Latu* (*The Central Association for Recreational Sports and Outdoor Activities*). En el año 1996, junto con Matti Heikkilä, director del laboratorio *Vierumäki Sports Institute and Aki Karihtala*, también vicepresidente de *Exel Oy*, y del director de producción de esta misma empresa, Taisto Manninen, desarrollan unos bastones especiales para esta nueva forma de caminar (6).

En este momento, la actividad se nombra oficialmente como *Sauvakävely*, "marcha con bastones" en finés, siendo sustituido más tarde a "Marcha Nórdica" o *"Nordic Walking"* por Exel, empresa que había desarrollado el primer bastón específico que registró con el nombre "*Nordic Walker*", en el año 1997 (6).

Aki Karihtala, fundador de *International Nordic Walking Asociation*, desarrolla "*How to introduce Nordic Walking Internationally*", que mezcla datos educacionales e informativos, con psicología, marketing y productos, que llegan a la población por diversos medios de comunicación, con el objetivo de impulsar la nueva actividad física que había nacido. Finlandia es el primer país donde se impulsa, siendo expandido a otros países tardíamente gracias a Exel. Finalmente, en el año 2000, se funda la *International Nordic Walking Asociation*, primera de las federaciones de marcha nórdica (6). En España, existe también delegación de la *International Nordic Walking Asociation* (7).

En el año 2008 nace la *World Record – Nordic Walking*, en 2011 *la Original Nordic Walking from Finland* y en 2015 la *European New Walking Organization* (5).

3.1.1.2 Técnica

A pesar de haber surgido como un recurso para las épocas estivales en sustitución del esquí de fondo, los bastones utilizados para la marcha nórdica son más cortos que los bastones de esquí, ya que las condiciones del terreno, sin nieve, son distintas. La recomendación acerca de la longitud del bastón que debe utilizar cada persona se realiza en función de la altura, quedando de la forma que se muestra en la Tabla 1 (8):

Tabla 1. Longitud del bastón de marcha nórdica en función de la altura de la persona.

Altura de la persona (cm)	Tamaño del bastón (cm)
<150	105-110
150-160	115
161-170	120
171-180	125

181-190	130
191-200	135
>200	140-145

Los bastones deben tener un agarre ergonómico que permita realizar una técnica correcta, a buen ritmo y con calidad de movimiento, un palo rígido y flexible, para que absorba el impacto, y ligero, y una punta especialmente diseñada para que se adapte a todo tipo de terreno (8).

Existen diferentes técnicas de Marcha Nórdica: ONW, INWA (ambas muy similares y correspondientes a la federación con la que comparten nombre), FITTREK y KEENFIT, aunque todas se desarrollan a partir de la misma técnica básica. Se camina realizando el movimiento natural, alternado de brazos y piernas, ayudándose de dos bastones de Marcha Nórdica. El apoyo del bastón derecho se realiza al mismo tiempo que el apoyo del pie izquierdo, y el apoyo del pie izquierdo, se realiza al mismo tiempo que el apoyo del bastón derecho (9,10).

2.1.1.2 Beneficios de la marcha nórdica

Realizando una búsqueda en términos generales en relación a la marcha nórdica, nos encontramos con la siguiente evidencia científica.

La marcha nórdica, en oposición a la marcha tradicional sin bastones, ha demostrado mayor trabajo mecánico y un mayor gasto energético, resultando sin embargo en una menor eficiencia metabólica. Esta disminución en la eficiencia metabólica es consecuencia de una mayor implicación de la musculatura superior del cuerpo, que aumenta la producción de trabajo, al acompañarse de contracciones isométricas y coactivaciones en la parte superior del cuerpo, resultando en el mismo desplazamiento, de ahí la reducción de eficiencia metabólica. El incremento en el trabajo mecánico y gasto energético es consecuencia del mayor movimiento del centro de masas durante la marcha (11).

El gasto energético es comparable en la caminata sin bastones y la marcha nórdica, cuando ambas se realizan con pendiente ascendente. Presumiblemente, esto ocurre porque el esfuerzo es realizado por las piernas en ambas actividades. Sin embargo, podemos deducir que, si pedimos al sujeto que ejerza fuerza con los bastones para impulsarse, el gasto energético se verá aumentado, comparado con la fuerza que ejerce espontáneamente (12).

Se ha demostrado también que previene el daño en las articulaciones de las extremidades inferiores y reduce la carga en las articulaciones de las rodillas (13). Esto es debido a que la

carga se distribuye de forma más uniforme por el cuerpo, trasladándola a la parte superior del tronco (14).

Aumenta el consumo de oxígeno en comparación a la marcha sin bastones, sin aumentar la fatiga, por tanto, aumenta los beneficios del ejercicio sin suponer un mayor esfuerzo para la persona (13). Esto es debido a la implicación de los músculos del tronco superior (12).

En cuanto a la marcha, aumenta la longitud de paso y en relación a la fase de apoyo, aumenta el tiempo de apoyo y reduce el tiempo de despegue. Dado que el apoyo se acompaña del apoyo de los bastones y el despegue se se combina con el empuje de los bastones contra el suelo. Gracias a esto puede ser beneficiosa para pacientes con dolor en las extremidades inferiores, artritis o diabetes, reduciendo los problemas resultantes de la presión plantar (13).

En cuanto a actividad muscular, se ha visto un aumento en la actividad del tríceps, y dorsal ancho, implicados en la fase de apoyo del bastón. Se observa también un incremento en la activación del bíceps braquial y del deltoides anterior y medio, y en todo el ciclo de marcha, ya que se coactivan durante la fase de extensión y apoyo del bastón, con el tríceps y el dorsal ancho. Mientras que en los miembros inferiores no se observan diferencias de activación respecto a la marcha normal: recto femoral, bíceps femoral, tibial anterior y gastrocnemios muestran una activación similar (12,13). Se ha observado también mayor activación del recto anterior, para estabilizar todo el ciclo de marcha y disminución de la contracción del erector espinal, remarcable esto último, teniendo en cuenta que un sobreuso del mismo suele desembocar en dolor lumbar, siendo por tanto recomendable la marcha nórdica en pacientes con dolor lumbar o de espalda en general (12).

Gran parte de los estudios que existen hasta la fecha sobre marcha nórdica tienen como muestra del estudio la población femenina. En estas pacientes, un estudio realizado por Park et al. (15)., ha demostrado disminuir el dolor lumbar, debido a la coordinación que se produce entre el movimiento de las extremidades inferiores y superiores, lo que favorece la posición neutra de la columna. Se desarrolla simultáneamente, como ya ha sido comentado, la musculatura superior e inferior. Se fortalece la musculatura paraespinal, lo que permite a los pacientes mantener la columna en una posición correcta durante un mayor período de tiempo, al estar trabajando en conjunto la musculatura abdominal, pélvica y sistema nervioso.

Se han demostrado beneficios en diversas patologías y condiciones que pueden estar afectando a la vida de la persona, relacionadas con un plan de entrenamiento basado en marcha nórdica. Por ejemplo, la **depresión**, que interviene en el normal desarrollo de la vida de quien la padece, aumentando la mortalidad e influyendo en el ámbito psíquico, físico y

social. La marcha nórdica disminuye la prevalencia de la depresión y la puntuación de depresión de la población estudiada, con diferencia significativa respecto a la marcha sin bastones. Se posiciona por tanto como una herramienta estabilizadora psicológicamente, una alternativa eficaz para el tratamiento de la depresión y de los trastornos del sueño en personas de avanzada edad (16).

Vílchez Barrera et al (17), realizan una revisión bibliográfica sobre la marcha nórdica y su uso en la Fisioterapia. En la misma analizan los resultados de estudios sobre marcha nórdica y: síndrome metabólico y obesidad (18–23), trastornos osteomusculares (24–26), enfermedad vascular (27,28), Parkinson, enfermedad pulmonar obstructiva crónica (EPOC) (4) e insuficiencia cardíaca (29). La conclusión general de la revisión es que la marcha nórdica debe ser incorporada como una forma de actividad física segura en un contexto fisioterapéutico. Se analizarán algunos de estos artículos pormenorizadamente a continuación (17).

En población femenina, Skórkowska-Telichowska et al. (2), estudiaron también los efectos en enfermedad arterial coronaria, fallo cardíaco, hipertensión arterial, hiperlipidemia, sarcopenia, enfermedad arterial periférica, síndrome metabólico y prótesis de cadera. Resulta beneficioso en la sarcopenia, reduciendo el riesgo en mujeres; para el síndrome metabólico en estadios tempranos y pacientes no diabéticos. En la rehabilitación post-prótesis de cadera, motiva a los pacientes al cumplimiento de la misma y ayuda a mantener la condición física y una buena condición musculoesquelética, importante para la mejora de la calidad de vida.

Para testar la eficacia en la **osteoartritis de cadera**, se ha analizado la actividad muscular alrededor de la articulación y el movimiento de la pelvis tras la práctica de marcha nórdica. Se concluye que puede prevenir el dolor de cadera y el dolor lumbar, además de prevenir discapacidades relacionadas (30). Todo esto se consigue gracias a la disminución de la activación del erector espinal, disminuyendo su sobreuso, y de la musculatura abductora y adductora de la cadera, lo que reduce la compresión a nivel de la articulación (12,30).

A continuación se analizan de manera más pormenorizada los resultados obtenidos con marcha nórdica sobre personas con patologías cardiovasculares, pues un alto porcentaje de pacientes con enfermedad pulmonar crónica presenta alguna comorbilidad cardiovascular (31).

El único metaánalisis sobre enfermedades cardiovasculares y marcha nórdica, analizó artículos de enfermedad arterial coronaria, enfermedad arterial periférica, insuficiencia cardíaca y accidente cerebrovascular (ACV) (32). En este la marcha nórdica solo se demuestra significativamente más beneficiosa combinada con rehabilitación cardiovascular

convencional en enfermedad arterial coronaria (33,34). En el estudio mencionado previamente de Skórkowska-Telichowska et al. (2), en enfermedad arterial coronaria, la marcha nórdica demuestra mejoras significativas respecto a la marcha normal en la capacidad de realizar ejercicio, medida en equivalentes metabólicos (METs), en el equilibrio dinámico y en la fatiga. Esta mejora coincide con la mejora que se produce en la tolerancia al ejercicio, la fuerza y la coordinación de la musculatura superior. Se considera por tanto un método efectivo y seguro.

En **ACV**, la marcha nórdica, realizada en cinta rodante al igual que la marcha sin bastones, demuestra mayores beneficios que ésta y una diferencia significativamente mayor en la distancia caminada en el test de 6 minutos marcha (6MWT) (35,36).

Del mismo modo la marcha nórdica ha demostrado ser una herramienta útil para el tratamiento no farmacológico de la **hipertensión arterial**, objetivándose un descenso de la presión arterial significativo después de un entrenamiento de este tipo, respecto al grupo control, estudiado en mujeres en edad menopáusica y mayores con obesidad. Se concluye su validez como un ejercicio lo suficientemente intenso, pero no demasiado vigoroso, que induce cambios favorables en la tensión arterial (2,37).

Tiene un efecto beneficioso en la profilaxis de las enfermedades cardiovasculares, ya que ha demostrado mayor eficacia para la reducción de peso corporal, glucosa en sangre, colesterol total, liporoteínas de baja densidad, lipoproteínas de alta densidad y triglicéridos, además de mayor adherencia al programa, comparado con un entrenamiento más convencional realizado con el método Pilates (2).

Por otra parte, la marcha nórdica permite a los pacientes con **insuficiencia cardíaca** aumentar la intensidad del ejercicio, y con ello aumentar los beneficios cardiorrespiratorios de forma segura: aumenta la captación de oxígeno, la frecuencia cardíaca máxima, la presión sistólica máxima y la fatiga, sin aparecer isquemia cardíaca ni arritmias significativas. Es una forma de ejercicio segura, bien tolerada y eficaz, que reduce el número de ingresos en el hospital por exacerbaciones (2). También en insuficiencia cardíaca ha sido demostrado que la marcha nórdica mejora el consumo de oxígeno (VO_2), la tasa de intercambio respiratorio, volumen espiratorio, la presión parcial de dióxido de carbono exhalado, la frecuencia cardíaca (FC), la presión arterial sistólica y la sensación de fatiga percibida según la escala de Borg, en relación al grupo que realizó marcha sin bastones. Este estudio comparó además estos resultados con el mismo protocolo en dos grupos de personas sanas, uno por cada grupo de entrenamiento, no obteniendo cambios en los mismos (38).

Otro estudio avala el uso de marcha nórdica en este tipo de pacientes, con insuficiencia cardíaca. Tras 12 semanas de intervención el 6MWT demostró una mejoría del 18% en los pacientes que realizaron marcha nórdica, aumentaron el tiempo libre que dedicaban a realizar ejercicio, la fuerza de agarre con la mano derecha, predictor de la salud del corazón (39), y los datos de la escala de ansiedad y depresión hospitalaria (HADS). No hubo, sin embargo, diferencias en VO_2, fuerza de agarre de la mano izquierda, peso corporal y circunferencia de la cintura (29).

Ha sido demostrada eficaz y segura, al no haberse producido ningún evento grave del tipo exacerbaciones u hospitalizaciones, también realizada de forma independiente en pacientes con insuficiencia cardíaca, telemonitorizada por profesionales, mejorando VO_2, duración de la carga de trabajo en un test de ejercicio cardiopulmonar, la distancia recorrida en 6MWT y la calidad de vida según el cuestionario de calidad de vida (QoL-Q) (40).

En la **enfermedad arterial periférica (EAP)** consigue mejorar la salud cardiovascular, funcional y calidad de vida (27,41–44). Además, respecto a la marcha tradicional, se consiguen mejoras en los tiempos de duración del ejercicio y en el consumo pico de oxígeno (VO_{2pico}) (28).

Spafford et al. (27), realizaron un estudio en el que compararon en pacientes con EAP la marcha nórdica con la marcha convencional, no demostrando cambios en la distancia de claudicación, viéndose aumentada en ambos grupos, al igual que la distancia caminada en el 6MWT; si bien es cierto que estos cambios fueron mayores en el grupo de marcha nórdica. La sensación de fatiga mejoró también de forma más importante en este grupo, lo que se tradujo en esos mayores cambios en la distancia caminada (41). Se demuestra también una mejora significativa en los valores obtenidos en el índice tobillo-brazo al final de los test iniciales con bastones, respecto al mismo test sin bastones, al igual que al final del entrenamiento, donde ambas formas de entrenamiento resultaron en unas mejores marcas, pero especialmente en el grupo de marcha nórdica. Se vio aumentado también el gasto calórico de forma significativa con respecto al grupo control. No se observaron diferencias en dolor, fatiga percibida ni colaboración de los participantes (27).

Un año más tarde se realizó un estudio complementario a este último, en el que se intentó analizar la adherencia en ambos grupos un año después de haber finalizado las sesiones: el grupo de marcha nórdica caminaba más distancia semanalmente y a una mayor velocidad media, además, superaban en un 21% la adherencia al ejercicio respecto al grupo control, el cual había dejado de mejorar, alcanzando estabilidad en las mediciones. Se concluye en este

estudio que un programa telemonitorizado basado en marcha nórdica es más beneficioso que uno basado en marcha tradicional (46).

Un estudio en el que se comparó un grupo de marcha normal en cinta rodante, con un grupo de marcha nórdica, y un grupo de entrenamiento combinado consistente en entrenamiento de resistencia y marcha nórdica, destacó como más beneficioso el entrenamiento combinado de resistencia y marcha nórdica, por ser el que más mejora la fuerza y además sirve para incrementar la distancia de claudicación. Se destaca también, que la marcha nórdica sirve de atractivo a la hora de asegurarse el cumplimiento y motivación con la pauta de entrenamiento (47).

Otro estudio confirma este incremento mayor de la distancia caminada en 6MWT tras un protocolo de marcha nórdica, además de una mayor motivación de los pacientes, que caminaban más distancia y más rápido (48). Esto último se confirma en el estudio de Collins et al. (28), en el que el único parámetro en el que se observan diferencias es la duración de la caminata, siendo mayor en el grupo de marcha nórdica, y siendo por tanto, más beneficiosa para los pacientes con EAP.

En cuanto al **síndrome coronario**, un ensayo clínico comparó un grupo control con pauta de entrenamiento tradicional de calistenia y ejercicio de resistencia con cicloergómetro, un grupo de marcha nórdica y un tercer grupo de marcha sin bastones (ambos combinados también con calistenia). Al final del entrenamiento el gasto energético medido con acelerómetro fue significativamente mayor en el grupo de marcha nórdica, no así, el gasto energético medido con el medidor de frecuencias. La capacidad de realizar ejercicio, medida en METs, se vió solo incrementada en los dos grupos de marcha. En el resto de test realizados (Fullerton, 6MWT, y *test get up and go*) la marcha nórdica resultó ser el método de entrenamiento más beneficioso (34).

Por otra parte, en el **síndrome metabólico** se ha demostrado que aumenta la capacidad de realizar ejercicio medida según el VO_{2pico}, en el peso corporal, en el índice de masa corporal (IMC) y en la circunferencia de la cintura, cambios significativos en el grupo de estudio, respecto a los controles, en aquellos pacientes que mostraban una satisfacción con su programa de entrenamiento igual o superior al 80% (18). Además, frente a un entrenamiento de resistencia, presenta mayores beneficios a la hora de disminuir el índice aterogénico plasmático y la puntuación y la prevalencia de la población con síndrome metabólico, sin realizar ningún cambio en la dieta (19).

En cuanto a enfermedades respiratorias, según nuestro conocimiento, la **EPOC**, es la única que ha sido estudiada en relación a un entrenamiento de marcha con bastones, o marcha nórdica.

Breyer et al. (4), en su estudio realizado en el año 2010, compararon un grupo de entrenamiento con marcha nórdica, realizado a una frecuencia cardíaca del 75% de la frecuencia cardíaca máxima inicial, 1 hora al día, tres veces por semana, durante 3 meses, con un grupo control que no recibió ningún tipo de entrenamiento. La marcha nórdica permite alcanzar en estos pacientes la intensidad de ejercicio deseada, medida por la frecuencia cardíaca, sin dificultad y realizando la técnica de forma correcta. En comparación al grupo control, se vió aumentada la actividad física diaria, al igual que el tiempo que pasaban caminando y el tiempo que permanecían de pie. La distancia caminada en el 6MWT fue también mayor, y la escala de Borg usada para medir la disnea, evidenció del mismo modo beneficios. Mediante la escala HADS se objetivó una mejoría significativa en los pacientes que realizaron marcha nórdica a los 3 meses, y esta mejoría se mantuvo en el tiempo (9 meses más tarde), del mismo modo que la calidad de vida, en contraposición al grupo control, cuyos datos permanecieron invariables.

Por último, en otro estudio realizado con pacientes EPOC se compararon distintos entrenamientos: entrenamiento tradicional de fuerza-resistencia supervisado frente a un entrenamiento combinado y supervisado solo de forma periódica consistente en marcha nórdica, circuito de entrenamiento y clases de aerobic (ejercicios aeróbicos de flexibilidad y equilibrio). El número de sesiones supervisadas fueron disminuyendo cada 5 semanas, empezando en 3 sesiones/semana. A partir de la 15º y hasta la semana 28º la totalidad de las sesiones se realizaron sin supervisión. Cada sesión consistía en 60 minutos en los que se alternaban los 3 tipos de ejercicio. Las sesiones de marcha nórdica duraban entre 10 y 20 minutos en los que los participantes caminaban a la velocidad escogida por ellos teniendo en cuenta que el esfuerzo percibido debía rondar los 3-4 puntos en una escala de Borg. Las clases de aerobic estaban dosificadas siguiendo el mismo criterio de intensidad, y consistían en ejercicios con peso libre realizados a 1 serie de 8 repeticiones. Por último, el circuito de entrenamiento, en el que los participantes realizaban diferentes tipos de ejercicios, como sentadillas o zancadas, realizando ente 10 y 15 repeticiones por ejercicio, y dando entre 2-4 vueltas al circuito. La adherencia fue mejor (100% frente a 87%) con el entrenamiento alternativo frente al convencional, siendo sin embargo una diferencia no significativa. En todas las mediciones, los resultados son comparables (49).

En cuanto a pacientes candidatos a **transplante de pulmón**, la marcha nórdica, tras un periodo de 12 semanas de entrenamiento, presenta una mejora significativa en el 6MWT de 64 metros y en el *Baseline Dyspnea Index* (BDI). La marcha nórdica se realizó en dos ciclos de 6 semanas cada uno, de las cuales las 2 primeras se realizaron en el hospital bajo la supervisión de fisioterapeutas y las 4 últimas las realizaron los pacientes en casa. Durante el entrenamiento se midió la FC y la saturación de oxígeno. Las recomendaciones seguidas para la realización de este fueron las de la guía de entrenamiento para pacientes con EPOC (50). El grupo control empeoró significativamente los valores alcanzados en la escala de *Medical Research Council* (MRC) respecto al grupo de estudio. Importante destacar que en este estudio el grupo control no recibió ningún tipo de entrenamiento (51).

El mismo grupo de investigación había realizado previamente un estudio basándose en el mismo entrenamiento, en el que se analizó la distancia recorrida en el 6MWT, la capacidad vital forzada y el volumen espiratorio forzado en el primer segundo (FEV_1), calidad de vida y el grado de disnea como una variación en el MRC, BDI o en el diagrama de coste de oxígeno. El grupo de estudio aumentó su distancia recorrida en 6MWT, 51,6m de media. De los valores espirométricos solo la capacidad vital forzada (FVC) mostró una mejoría significativa y el cuestionario *Short Form Health Survey (*SF-36) usado para valorar la calidad de vida muestra especialmente mejoras en la función social y física, mejoras que se mantienen significativas también 12 semanas después del programa de rehabilitación. No se muestran cambios significativos en el grado de disnea (52).

2.1.2 Asma y Fisioterapia

Dentro de lo que se conoce como asma se encuentran diversos fenotipos que comparten manifestaciones clínicas similares, pero de etiología diferente. Se considera una enfermedad inflamatoria crónica de las vías respiratorias, en cuya patogenia intervienen diversas células y mediadores de la inflamación, condicionada por factores genéticos y que cursa con hiperrespuesta bronquial y una obstrucción al flujo aéreo variable, que puede ser total o parcialmente reversible: por acción medicamentosa o espontáneamente. La exacerbación asmática se produce por estrechamiento de la vía aérea, que produce la obstrucción del músculo liso bronquial, edema e hipersecreción de la mucosa. Es característica de esta enfermedad la hiperrespuesta bronquial, definida como respuesta broncoconstrictora exagerada a una variedad de estímulos físicos, químicos o biológicos (53).

Los signos y síntomas característicos del asma son disnea, tos, sibilancias y presión torácica. Estos síntomas y signos son susceptibles a las variaciones estacionales y a los antecedentes

familiares y personales de atopia. Dado que son síntomas comunes a otras enfermedades, para su diagnóstico es necesaria la realización de pruebas funcionales respiratorias (53).

El asma se clasifica en función de los síntomas diurnos y nocturnos, la medicación de rescate, la limitación de la actividad, la función pulmonar (FEV_1) flujo espiratorio máximo (PEF) % teórico y exacerbaciones, como vemos en la Tabla 2 (53).

Tabla 2. Clasificación del asma en función de los síntomas.

	Intermitente	Persistente leve	Persistente moderada	Persistente grave
Síntomas diurnos	No (2 días o menos a la semana)	Más de dos días a la semana	A diario	Continuos y varias veces al día
Medicación de alivio	No (2 o menos días a la semana)	Más de dos días por semana, pero no a diario	Todos los días	Varias veces al día
Síntomas nocturnos	No más de dos veces al mes	Más de dos veces al mes	Más de una vez a la semana	Frecuentes
Limitación de la actividad	Ninguna	Algo	Bastante	Mucha
Función pulmonar (FEV_1 o PEF) % teórico	>80%	>80%	>60-80%	≤ 60%
Exacerbaciones	Ninguna	Una o ninguna al año	Dos o más al año	Dos o más al año

FEV_1: Volumen espiratorio forzado en el primer segundo; PEF: flujo espiratorio máximo

Se considera exacerbación a los episodios agudos o subagudos caracterizados por un aumento progresivo de uno o más de los síntomas típicos (disnea, tos, sibilancias y opresión torácica) acompañados de una disminución del PEF (53).

El asma es abordado desde la Fisioterapia a través de dos enfoques: ejercicios respiratorios (54–59) y ejercicio físico, generalmente aeróbico (55,60). Existen dos revisiones sistemáticas recientes que analizan el asma y su intervención desde la Fisioterapia en el paciente adulto (61,62), y una orientada al análisis de sus beneficios en población infantil (63). Una de las

revisiones sistemáticas en adultos se realiza a partir de 21 ensayos clínicos, de las cuales solo once están centrados en el ejercicio terapéutico como tratamiento para el asma (61). De estos once, seis evalúan la mejora de la calidad de vida en relación al ejercicio (64–69), obteniendo en cinco de ellos una mejora significativa (64–68). En cuanto a los síntomas, fueron analizados en tres de los estudios (64,65,70), observándose mejora significativa en uno de ellos (70) y aumento de los días libres de síntomas en otro (64). Solo uno de los estudios valoró el control del asma, que había mejorado significativamente (66), y el uso de medicación disminuyó en uno (67) de los tres ensayos en los que se estudió (65,67,68). El VO_2 mejoró en la totalidad de estudios que lo analizaron (64,68,70), el FEV_1 solo en uno de los nueve en los que se estudió (71); FVC en uno de los siete (71) y PEF en uno de los cuatro (72). Se concluye que el ejercicio terapéutico puede mejorar la calidad de vida, disminuir los síntomas y mejorar la resistencia cardiopulmonar y la condición física, sin cambios en la función pulmonar (61).

Por último, las conclusiones obtenidas en niños son similares a las mencionadas anteriormente: la Fisioterapia, y concretamente el ejercicio físico y deporte (como baloncesto, natación, ciclismo, ejercicio en cinta, ejercicio en casa…), mejoran la función cardiorrespiratoria (consumo máximo de oxígeno (VO_{2max}), pulso de oxígeno, umbral ventilatorio) y sus beneficios en la función pulmonar se limitan únicamente al PEF. En cuanto a calidad de vida se muestra una tendencia positiva, si bien los resultados no son concluyentes (63).

Existe otra revisión sistemática y metanálisis, realizada exclusivamente con ejercicio físico como herramienta terapéutica. Todos los grupos de estudio mejoraron los síntomas respecto a los controles; en cuanto a hiperreactividad bronquial se demuestra una tendencia significativamente positiva a favor del grupo de estudio, al igual que la capacidad aeróbica medida según VO_{2max}, carga máxima de trabajo y resistencia al ejercicio. La calidad de vida mejoró también de forma significativa, aunque fue imposible realizar un metanálisis de los datos debido a la variedad de cuestionarios empleados, igual que con los síntomas (62).

Diferentes estudios han demostrado que el ejercicio aeróbico reduce la hiperreactividad bronquial, las citoquinas proinflamatorias, mejora la calidad de vida y la exacerbación de los síntomas en pacientes asmáticos (55). Al igual que mejora el $VO_{2máx}$, pulso de oxígeno, volumen espiratorio y la tasa máxima de trabajo. En cuanto a la calidad de vida, se produce una mejora estadísticamente significativa, aunque no lo suficientemente elevada para sobrepasar el umbral de la significación clínica, y según el *Asthma Quality of Life*

Questionnaire (AQLQ), la mejora sí se produce de forma también significativa, pero solo en las actividades, emociones y calidad de vida general, no así en los síntomas (60).

2.2 Justificación del trabajo

Según se ha visto en la revisión de la literatura realizada para la contextualización de este trabajo, hasta la fecha, no existe bibliografía que evidencie la validez de un entrenamiento basado en marcha nórdica en pacientes con asma. Ese es por tanto el objetivo de este trabajo, a través del cual, se pretende obtener una posible pauta de entrenamiento para personas diagnosticadas con asma, que genere la adherencia suficiente y los beneficios necesarios para obtener una mejora en su condición física, en su salud y en su calidad de vida.

La posibilidad de realizar este entrenamiento, al que nos podríamos referir como tratamiento, es especialmente interesante, ya que sería realizado en un contexto de entornos urbanos verdes y azules, siguiendo el modelo de trabajo de *Urban Training*®, que ha demostrado mejores resultados que los cuidados convencionales en pacientes con EPOC y que además encaja en el modo de vida de una cultura mediterránea (73), resultados que podría ser extrapolables a pacientes con asma, dadas las similares características de ambas patologías: en ambas existe una limitación al flujo aéreo (aunque reversible en el caso del asma), y comparten del mismo modo síntomas, como tos, disnea y limitación de la tolerancia al ejercicio (53,74).

3. Hipótesis y objetivos

3.1 Hipótesis: nula y alternativa

Hipótesis nula

La marcha nórdica no presenta beneficios añadidos a un plan educacional en pacientes con asma.

Hipótesis alternativa

Existen diferencias estadísticamente significativas entre la aplicación de un programa de marcha nórdica y de un plan educacional frente a la aplicación exclusiva de un plan educacional en pacientes con asma, a favor del primero.

3.2 Pregunta de investigación

¿Ofrece la marcha nórdica beneficios añadidos frente a la aplicación exclusiva de un plan educacional en pacientes con asma?

3.3 Objetivos: generales y específicos

Objetivos generales

- Analizar si existen diferencias, estadísticamente significativas, en relación a la tolerancia al ejercicio y calidad de vida, en personas con asma, cuando al procedimiento habitual de un plan educacional se suma un programa de entrenamiento basado en marcha nórdica.
- Analizar la experiencia de los pacientes con respecto a la marcha nórdica como tratamiento para el asma.

Objetivos específicos

- Analizar si existen diferencias estadísticamente significativas en personas con asma, cuando al procedimiento habitual de un plan educacional se suma un programa de entrenamiento basado en marcha nórdica en relación a:
  - Tolerancia al ejercicio físico.
  - Calidad de vida.
  - Actividad física semanal.
  - Función pulmonar.
  - Aparición y control de signos y síntomas.
  - Número de visitas a urgencias.
  - Número de veces que los pacientes necesitan recurrir a un inhalador de rescate.
  - Adherencia al tratamiento.
- Analizar, desde un enfoque cualitativo, la experiencia del grupo de marcha nórdica en relación a:
  - Forma de afrontar la enfermedad.
  - Satisfacción con la intervención realizada.
  - Progreso percibido en el manejo de su enfermedad tras la intervención.

4. Metodología

4.1 Ámbito de estudio

Para la realización de este estudio se seleccionarán personas diagnosticadas de asma, mayores de edad, seleccionadas por los responsables del área de Neumología del Hospital HM Modelo de A Coruña, y de las áreas de Alergología y neumología del Complejo Hospitalario Universitario de A Coruña (CHUAC). Se basarán en los criterios de inclusión y

exclusión, y ofrecerán a los pacientes la posibilidad de participar en el estudio. De aceptar, será el propio paciente el que se ponga en contacto con nosotros.

Una vez elegidos a los participantes, estos serán distribuidos de forma aleatoria en el grupo control o grupo de estudio.

4.2 Período de estudio

El período de estudio comprende desde diciembre de 2018, que se inicia este proyecto, hasta marzo de 2024, cuando se planea que la investigación esté concluida.

4.3 Tipo de trabajo

Se trata de un ensayo clínico aleatorizado simple ciego, en el cual, solo la persona que realiza las evaluaciones y la que analiza los datos desconocen a qué grupo pertenecen los mismos.

4.4 Criterios de selección

Criterios de inclusión:

- Personas mayores de 18 años.
- Con diagnóstico médico de asma.
- Personas que deseen participar en el estudio.
- Personas con capacidad de firmar el consentimiento informado.

Criterios de exclusión:

Se usan como criterios de exclusión las contraindicaciones para el ejercicio cardiovascular de la Sociedad Americana del Corazón (75), las contraindicaciones para la realización de un 6MWT según la *American Thoracic Society European Respiratory Society* (ATS/ERS) (76), de la espirometría según los criterios de la SEPAR (77). Todos ellos están disponibles en el Anexo 1. Se añaden los siguientes criterios, que son los utilizados en el estudio realizado con marcha nórdica y pacientes con EPOC (4), y los de un estudio realizado siguiendo un protocolo de entrenamiento en pacientes con asma (66):

- Contraindicaciones para la realización del 6MWT según la ATS/ERS (Anexo 1).
- Contraindicaciones para la realización de una espirometría según la SEPAR (Anexo 1).
- Personas que presenten otros diagnósticos de enfermedad respiratoria además de asma (EPOC, bronquiectasias, etc).
- Exacerbación de asma en las últimas 12 semanas. Se define exacerbación como episodios de empeoramiento de la situación basal del paciente que requieren

modificaciones en el tratamiento. Se identifican por cambios en los síntomas, en la medicación de alivio, o en la función pulmonar respecto a la variación diaria de un paciente en concreto. Retrospectivamente, también se podrían identificar por un incremento de la dosis del tratamiento de mantenimiento durante al menos 3 días. En el Anexo 1 de contraindicaciones aparece también una tabla clasificatoria de la gravedad de la exacerbación asmática (53).

- Infarto agudo de miocardio en los últimos 6 meses.
- Arritmias cardíacas de grado mayor a IIIb en escala Lown.
- Trastornos de la marcha debido a problemas del sistema músculo-esquelético.
- Infección respiratoria en las últimas 4 semanas.
- Fumadores.
- Exfumadores de menos de 2 años.
- Comorbilidades que impliquen capacidad reducida de realizar ejercicio: anemia importante, desequilibrio electrolítico o hipertiroidisimo.
- Participantes actuales en sesiones de ejercicio de más de 30 minutos/día de forma moderada o vigorosa.
- Participación en un programa de rehabilitación pulmonar en los últimos 12 meses.
- Mujeres embarazadas y lactantes.

4.5 Justificación del tamaño muestral

El tamaño muestral se selecciona en función de la distancia caminada en el 6MWT por considerarse la variable resultado principal del estudio. Conociendo los datos de la mínima diferencia clínicamente relevante (MCID), 30,5m (78) y usando el dato de desviación típica del estudio de Coelho et al. (79), en el que los sujetos con asma presentaron una desviación típica de 41,5m en el 6MWT, para una hipótesis bilateral, introduciendo una significación del 0,05, un poder estadístico del 80%, y suponiendo un 10% de pérdidas a lo largo del período de entrenamiento, **se requieren 34 sujetos por grupo**. Para el cálculo del tamaño muestral se empleó la herramienta creada por la Unidad de Epidemiología Clínica y Bioestadística del CHUAC (https://bit.ly/2JsRUzh).

El cálculo del tamaño de la muestra (n=68) se ha llevado a cabo para garantizar que a pesar de que se produzcan pérdidas de seguimiento (estimadas en un 10%) el estudio tenga el poder suficiente para observar diferencias estadísticamente significativas entre los grupos (en caso de haberlas). Además, el impacto de las pérdidas de seguimiento se evaluará a posteriori comparando las características basales de los pacientes que no finalicen el estudio y los pacientes que lo completen según lo previsto.

4.6 Selección de la muestra

Se enviará la carta de invitación a participar a los jefes del servicio de Neumología y Alergología del CHUAC y de Neumología del Hospital HM Modelo, para que puedan informar a sus pacientes sobre la realización de este estudio durante una visita rutinaria a consulta en su centro de referencia. Serán los responsables de cada área, investigadores colaboradores de este estudio, los que recluten a los participantes. Dra. Larissa Patricia Fontán García Boente como responsable del área de Neumología del Hospital HM Modelo, la Dra. Marina Blanco Aparicio como responsable de Neumología del CHUAC y el Dr. Manuel Jorge Rial Prado como responsable del área de Alergología del CHUAC.

Una persona ajena a la investigación distribuirá a los participantes en ambos grupos mediante un programa informático de aleatorización.

4.7 Descripción de las variables

Se detallan a continuación las variables de estudio, resumidas en la tabla 4.

4.7.1 Tolerancia al ejercicio

Se utilizará el 6MWT para medir la capacidad de realizar ejercicio y la dificultad respiratoria asociada al mismo. Es un test validado por la *American Thoracic Society* (76) con una MCID conocida, de 30,5 metros (78).

4.7.2 Nivel de actividad física diaria

Las herramientas utilizadas para la objetivación de los cambios conseguidos en este aspecto serán:

- *International Physical Activity Questionnaire* (IPAQ) versión corta, cuestionario validado (80), en su versión en castellano.
- Número de pasos. Los pacientes deberán anotar el valor de la app en diario en su diario del paciente (Anexo 2).

4.7.3 Calidad de vida

En cuanto a la calidad de vida, serán usados tres cuestionarios para analizar los cambios conseguidos en dicha variable:

- *SF-36, cuestionario validado* (81), también en español (82), con MCID, clasificada en función de la significación de la misma (significación baja, moderada o alta), en sus 8 secciones. Tabla 3 (83).

Tabla 3. Mínima diferencia clínicamente significativa clasificado por significancia y por secciones del Short Form - Health Survey.

	Significación baja	Significación moderada	Significación alta
Función física	10	20	30
Limitación por problemas físicos	12,5	25	30
Dolor corporal	10	20	37,5
Salud General	10	20	30
Vitalidad	12,5	25	37,5
Papel social	12,5	25	37,5
emocional	16,7	33,3	50
Salud mental	10	20	30

- *Asthma Quality of Life Questionnaire* (AQLQ), cuestionario validado en pacientes con asma (84), así como validada su versión en español (85), con MCID conocida: 0,5 puntos de diferencia (86).

4.7.4 Control del asma y síntomas

Para el control del asma se usará el cuestionario *Asthma Control Test* (ACT) (87), con una MCID de 2,2 de media (88) y validado para su uso con población hispanohablante (89). Se usará también el cuestionario Test de Adhesión a los Inhaladores (TAI), cuestionario validado en español (90). En cuanto a los síntomas, se analizarán las características e intensidad de estos y los días que los pacientes han pasado sin síntomas a través del mismo diario utilizado para las mediciones anteriores (Anexo 2).

4.7.5 Disnea

Se usará la escala de Borg (91) y la *Modified Medical Research Council* (mMRC) (92).

4.7.6 Función pulmonar

La función pulmonar debe ser medida regularmente en pacientes asmáticos (93), utilizando para ello una espirometría forzada, con la cual obtendremos los valores de FEV_1 y FVC así como el cociente entre ambos parámetros.

El PEF sirve para medir la variación o fluctuación de los síntomas y de la función pulmonar en el tiempo, es decir, la variabilidad característica del asma. El paciente realizará la medición del mismo a diario, con un medidor portátil de PEF, y deberá anotar el valor del mismo en el diario de síntomas (Anexo 2) (53,94).

4.7.7 Fuerza musculatura respiratoria

Se medirá también la presión inspiratoria máxima (PIM) y la presión espiratoria máxima (PEM).

4.7.8 Fuerza-resistencia de la musculatura

Se realizará una medición de la fuerza de agarre con un dinamómetro (Jamar Dynamometer) (95). Esta medición ha sido validada como útil y confiable para valorar la fuerza muscular en un contexto clínico (96). La fuerza de agarre está relacionada con la mortalidad y morbilidad a corto plazo, con el riesgo de hospitalización, re-hospitalización, complicaciones postquirúrgicas, pérdida de independencia, limitaciones funcionales y es un marcador del estado nutricional del paciente (97).

4.7.9 Toma de medicamentos

A través de un diario que se proporcionará al paciente (Anexo 2).

4.7.10 Visitas a urgencias

A través de un diario que se proporcionará al paciente, y en relación con el apartado anterior (Anexo 2).

4.7.11 Adherencia

A través de un diario que se proporcionará al paciente (Anexo 2).

En la tabla 4 se muestran las variables de estudio elegidas para este ensayo clínico y el instrumento de medida correspondiente a cada una de ellas.

Tabla 4. Variables de estudio e instrumentos de medida.

VARIABLE DE ESTUDIO	INSTRUMENTO DE MEDIDA
Tolerancia al ejercicio	Test de 6 minutos marcha (6MWT)
Nivel de actividad física	Cuestionario internacional de actividad física (IPAQ)<br>APP (contador de pasos)
Calidad de vida	*Short-form Health Survey*<br>*Asthma Quality of Life Questionnaire*
Control del asma y síntomas	*Asthma Control Test*<br>Test de adhesión a los inhaladores<br>Diario del paciente
Disnea	Escala de Borg<br>*Medical Research Council modificada*
Función pulmonar	Espirometría<br>Medidor de flujo espiratorio máximo (Peak-Flow)

Presiones respiratorias máximas	Medidor de PIM y PEM
Fuerza-resistencia de la musculatura	Dinamómetro
Toma de medicamentos	Diario del paciente
Visitas a urgencias	Diario del paciente
Adherencia	Diario del paciente

4.7.12. Datos cualitativos

Se recogerán datos cualitativos a partir de una entrevista semi estructurada grupal en formato grupos focales, con el objetivo de conseguir un mejor entendimiento de la experiencia de los participantes con la actividad propuesta (marcha nórdica), y si es posible, comparar estos hallazgos cualitativos, relacionándolos y completándolos, con la información cuantitativa, proporcionando por tanto una visión comprehensiva entre ambos enfoques, mediante un estudio de método-mixto.

4.8 Mediciones e intervención

4.8.1 Mediciones

Todas las mediciones serán llevadas a cabo por la misma fisioterapeuta previamente entrenada para ello. Se realizarán pre y postratamiento, así mismo, a los 3 y 6 meses de la última sesión, a fin de evaluar los efectos a medio y largo plazo.

4.8.1.1 Entrevista personal y exploración física

Se realizará una entrevista personal, cuyo cuaderno de recogida de datos se puede consultar en el Anexo 5, en el cual consta la siguiente información:

- Datos sociodemográficos: edad, sexo, nacionalidad, situación laboral.
- Hábitos tabáquicos.
- Antecedentes patológicos.
- Salud general.

En la exploración física se registrarán las medidas antropométricas con una báscula con tallímetro (Seca 700, Hamburgo, Alemania), con el sujeto descalzo, la cabeza erguida, peso repartido sobre ambos talones y con los bolsillos vacíos. En ese momento se realizarán también el resto de las mediciones de esta investigación: cuestionarios de tolerancia al ejercicio y calidad de vida, 6MWT, espirometrías y medición de la fuerza de agarre; y los pacientes serán instruidos para cubrir correctamente su diario del paciente (Anexo 2), información que será reforzada durante la clase del plan educacional.

4.8.1.2 Tolerancia al ejercicio

Test de 6 minutos marcha

Se realizará el 6MWT, según las indicaciones de la guía para la realización del test de la ATS/ERS (76). Se llevará a cabo en un pasillo interior de 30 metros de largo delimitados por dos conos situados a una distancia de 29 metros (quedando un espacio de 0,5 m a cada lado para los giros). Los participantes serán incentivados a "caminar lo más rápido posible" sin llegar a correr. No deberán hablar ni distraerse durante el recorrido, y en caso de necesitarlo, se les indicará que pueden detenerse o aminorar el paso, pero que en ningún momento el cronómetro dejará de contar.

Se tomarán los signos vitales (FC, saturación de oxígeno y frecuencia respiratoria), así como la sensación de fatiga y disnea según la escala de Borg, antes y después de la realización de la prueba. Se registrarán número de paradas, tiempo total de las mismas y sus motivos. También se anotarán los metros recorridos. Al final de la prueba, se monitorizarán los signos vitales durante los primeros 5 minutos, para comprobar que el paciente se recupera correctamente. Se realizarán dos pruebas con un intervalo intermedio de 30 minutos de descanso. Todos estos datos son recogidos en la hoja de registro (Anexo 6).

4.8.1.3 Nivel de actividad física

IPAQ

Usaremos el cuestionario IPAQ en su versión corta (Anexo 7), que será administrado mediante entrevista personal. Los resultados se analizarán según la guía del comité de investigación del IPAQ (98).

APP (CONTADOR DE PASOS)

Se utilizará una aplicación móvil encargada de contar los pasos en el móvil del paciente, para contabilizar los pasos que dan en su día a día. Los pacientes serán instruidos en su uso y deberán escribir en su diario los pasos realizados cada día (Anexo 2).

4.8.1.4 Calidad de vida

Los cuestionarios de calidad de vida SF-36 (Anexo 8), AQLQ (Anexo 9) serán administrados a los sujetos mediante entrevista personal.

4.8.1.5 Control del asma y síntomas

Se usará la escala ACT (Anexo 10) para valorar el control de síntomas que presenta el paciente. Se usará el Test de Adhesión a los Inhaladores para evaluar la toma de los mismos (Anexo 11).

Además, en el diario del paciente (Anexo 2), deberá anotar información relativa a la disnea (según escala de Borg), expectoración, tos, sibilancias (sensación de pitidos en el pecho) toma de medicamentos y visitas a urgencias, como instrumento de valoración para el control de lo síntomas.

4.8.1.5 Disnea

Para valorar la disnea se aplicará la escala mMRC durante la entrevista personal, escala que aparece en el Anexo 12. Además, antes y después del ejercicio se preguntará acerca de la sensación de disnea según la escala de Borg modificada. Durante el ejercicio, se pedirá del mismo modo que se gradúe la disnea según la misma escala, cada 5 minutos transcurridos. Dicha escala aparece como parte del Anexo 13.

4.8.1.6 Función pulmonar

Para la realización de las espirometrías se seguirán las recomendaciones internacionales de la ATS/ERS y se realizarán con un espirómetro Datospir® 120C (Sibel Group, Barcelona, España), y se registrarán los datos de FEV_1, FVC y FEV_1/FVC obtenidos tras una espirometría forzada. Se usará una boquilla desechable que debe estar colocada y bien sellada alrededor de los labios del paciente. Desde la capacidad residual funcional (CRF) se solicitará al paciente una maniobra inspiratoria máxima, rápida, pero no forzada. A continuación, y tras una apnea inferior a 1 segundo en capacidad pulmonar total, se pedirá espiración máxima, rápida y forzada hasta completar el vaciado de los pulmones (99).

El paciente deberá, por su cuenta, realizar una medición del PEF de forma diaria, con un medidor *Peak Flow* (asmaPLAN+, Vitalograph®) acoplado a una boquilla en tubo. Se indicará a los pacientes que para esta medición deberán exhalar todo el aire fuera del dispositivo hasta el volumen de reserva espiratorio, inspirar profundamente después hasta TLC, introducir la boquilla sellando bien los labios alrededor de la misma y realizar una espiración lo más fuerte posible a través del dispositivo. Dicha medición deberá ser realizada todos los días a la misma hora (al levantarse por las mañanas) y en las mismas circunstancias, un total de 3 veces, anotándose en el diario del paciente (Anexo 3), solo el más alto de esos valores (100).

4.8.1.7 Fuerza de la musculatura respiratoria.

Se medirán la PIM y la PEM siguiendo las recomendaciones nacionales de la SEPAR (101) y empleando el dispositivo Micromedical RPM Carefusion conectado al software PUMA®. La medición de las presiones respiratorias máximas es una de las pocas evaluaciones de función pulmonar que presenta desacuerdo entre las sociedades científicas nacionales e internacionales sobre su protocolo (76,101). Nos hemos decantado por la aplicación del protocolo propuesto por SEPAR dado que ha sido demostrado que con éste se obtienen

valores más altos de PIM y PEM (102). Los/as participantes se situarán en la misma posición que en el caso de las espirometrías. Como material adicional se empleará una boquilla de tipo submarinista de material rígido, con una arandela para situar entre la cara interna de los labios y las encías, impidiendo las fugas de aire, así como pinzas nasales. Antes de realizar las pruebas el/la paciente deberá estar 5 minutos relajado/a y sentado/a cómodamente, momento que aprovecharemos para darle las explicaciones oportunas. Se comenzará midiendo la PEM por ser más fácil de entender y ejecutar. Es importante tener en cuenta que entre la valoración de ambas presiones se descansará 5 minutos, y entre la realización de maniobras del mismo tipo se dará un minuto de descanso. Se efectuarán hasta un máximo de 10 intentos para obtener 6 maniobras técnicamente aceptables, lo cual significa que hayan durado entre 3 y 5 segundos, que no se evidencien fugas de aire y que haya tendencia a la meseta en la curva de ambas presiones. Además, se buscarán 3 maniobras reproducibles entre las 6 aceptables (es decir, con una variabilidad inferior al 5%). Entre las 3 maniobras aceptables y reproducibles se seleccionará la de mayor valor, medida en cmH2O (101). Los resultados obtenidos serán comparados con los valores de referencia para población española adulta sana propuestos por Morales et al. (103). Página 31 De acuerdo con los límites inferiores de normalidad recogidos en la literatura (104) consideraremos la existencia de debilidad muscular cuando la PIM y la PEM se encuentren por debajo del 80% al 65% del valor de referencia.

4.8.1.8 Fuerza de agarre

Se realizará una medición de la fuerza de agarre con ambas manos, a través del uso de un dinamómetro (Jamar Dynamometer). Para la medición se colocará el puño del dinamómetro en la segunda posición más pequeña, y las segundas falanges deberán descansar alrededor del mismo. De no ser así, se reajustará la posición. Primero, el paciente verá una demostración de la medición realizada por el fisioterapeuta encargado de la misma, que apretará una pequeña pelota. Después, el paciente probará el mismo a apretar dicha pelota (95). Posteriormente, el paciente realizará él mismo movimiento en el dinamómetro, en posición sentada, con el brazo pegado al tórax, rotación neutra, codo flexionado en 90 grados y antebrazo y muñeca en posición neutra (105). Se anotará el mejor resultado de tres mediciones realizadas con cada mano, medidas en kilos. El fisioterapeuta debe estar seguro de que el esfuerzo del paciente sea el máximo, fijándose para ello en su expresión facial, contracción de los músculos del brazo y antebrazo, coloración de las falanges, que estarán blancas, y una concordancia entre las tres mediciones (86).

4.8.1.9 Toma de medicamentos

El paciente deberá anotar en su diario del paciente (Anexo 2) cuándo, qué y en qué cantidad necesita administrar medicamentos para aliviar los síntomas.

4.8.1.10 Visitas a urgencias

El paciente deberá anotar también en el diario del paciente (Anexo 2) las visitas a urgencias que realice.

4.8.1.11 Adherencia

La adherencia al entrenamiento se valorará tras analizar los datos que el paciente haya recogido en su diario de síntomas (número de pasos dados al día) y los resultados del cuestionario IPAQ.

4.8.1.12 Datos cualitativos

Con el objetivo de analizar la experiencia y la satisfacción de los participantes con la intervención de marcha nórdica, el grupo que realiza marcha nórdica, será citada para realizar una entrevista en formato grupos focales, junto a los integrantes de su grupo de entrenamiento, una vez terminado este. Por tanto, cada grupo focal estará formado por 5 personas (las 5 integrantes de cada grupo de marcha nórdica), y durante las mismas, se realizarán preguntas semiestructuradas de final abierto (se pueden consultar en el Anexo 15). Los participantes podrán responder libremente e interactuando entre ellos. La entrevista de grupos focales será dirigida por una persona experta en este tipo de metodología cualitativa y tendrá lugar en la Facultad de Fisioterapia de la Universidade da Coruña, con una duración aproximada de una hora (se considerará finalizado cuando se sature la información). Otra persona asistente participará en las sesiones de forma pasiva encargándose de la toma de notas. Estas dos personas iniciarán la reunión presentándose y dando información clara sobre el objetivo de la reunión. Los participantes, que habrán firmado el consentimiento informado para ello, serán gravados en vídeo y audio, para la posterior transcripción y análisis de los datos recabados durante estas entrevistas.

4.8.2 Intervención

Se citará a los pacientes de forma inicial para realizar la entrevista clínica y la realización de todas las mediciones y pruebas.

Todos los participantes, grupo de estudio como grupo control, serán instruidos en dos clases de dos horas cada una, en un plan educacional orientado a conocer y manejar su patología, así como a proporcionar educación a los pacientes, relativa a hábitos de vida saludables. El esquema del plan educacional es el de la tabla 5, apareciendo desglosado en el anexo 14. A

todos los participantes se le entregará una guía explicativa sobre su enfermedad y con indicaciones y consejos para el manejo de ésta (Anexo 16).

Tabla 5. Plan educacional para los participantes.

Información general sobre el asma	¿Qué es el asma? Definición y descripción<br>Prevalencia<br>Fenotipos del asma: “tipos de asmáticos”<br>Control del asma
Medicación	¿Qué? Tipos de medicamentos<br>¿Cómo? Correcta administración de los inhaladores
Control del ambiente y estilo de vida	Ambiente óptimo<br>Nutrición<br>Ejercicio físico y vida activa
Técnicas de cuidado del asma	
Guía asma	

Únicamente el grupo de estudio será sometido a un programa de entrenamiento, detallado en la tabla 5. Este programa está basado en las recomendaciones de la ATS/ERS para rehabilitación pulmonar (106), así como en el estudio de Breyer et al (4)., en el que se analizaron los efectos de un entrenamiento basado en marcha nórdica en pacientes con EPOC, ya que no existe literatura relativa a la marcha nórdica y asma, y en la guía de Garber et al.(107), para la prescripción de ejercicio.

El material necesario para el desarrollo de la sesión consistirá en un par de bastones de marcha nórdica Power Poles (Leki; Hamburg, Germany) (4), y reloj medidor de frecuencia cardíaca Polar® H7 beat, para controlar la intensidad del ejercicio, que se establecerá entre el 50 y el 75% de la frecuencia cardíaca máxima teórica (FCmáx) y entre 4 y 6 en una Escala de Borg modificada para fatiga y disnea, intensidad a la que se produce la adaptación fisiológica al ejercicio, para la que también es necesario un mínimo de 20 sesiones, por lo que dado que la frecuencia recomendada son 3 sesiones/semana, necesitaremos un mínimo de 7 semanas . Estableceremos un total de 8 semanas, o 2 meses (106).

El plan de entrenamiento aparece desglosado y detallado en el anexo 17 y resumido en la tabla 6.

Tabla 6. Plan de entrenamiento.

	Tiempo	Tipo de ejercicio
Calentamiento	5 minutos	Movilidad articular/ estiramientos dinámicos.
	10 minutos	Caminar a ritmo ligero.
Parte central	30 minutos	Inicialmente 50% de la FCmáx teórica<br>Se incrementa 5% de Intensidad por semana hasta alcanzar 75% de la FCmáx teórica.
Vuelta a la calma	10 minutos	Estiramientos
	5 minutos	Relajación
3 sesiones/semana		
Duración total: 8 semanas		

El grupo de estudio será instruido de forma grupal en una sesión previa de marcha nórdica. Las sesiones restantes serán también grupales. Los grupos estarán formados por 5 pacientes cada uno.

Las sesiones tendrán lugar en el paseo marítimo de A Coruña y por la zona vieja y la Marina, un terreno asfaltado y sin demasiados cambios de nivel, y serán realizadas por una fisioterapeuta, entrenada en Finlandia para la puesta en práctica de marcha nórdica.

En caso de necesitarlo el paciente usará la medicación de rescate habitual, para exacerbaciones del asma.

Las mediciones serán realizadas por una persona distinta a la persona que lleva a cabo la intervención y a la que indexa y analiza los datos, antes de la intervención y al finalizar la misma. Además, se realizarán las mismas mediciones de seguimiento a los 3 y 6 meses de la última sesión.

4.9 Análisis estadístico

Se realizará un análisis descriptivo de las variables incluidas en el estudio. Las variables cualitativas se expresarán con sus valores absolutos y porcentajes. De las variables cuantitativas se calcularán su media, desviación estándar, mediana, cuartiles y valores máximos y mínimos.

Para el análisis de las variables resultado se realizará un ANOVA de medidas repetidas o un test de un test de Kruskal- Wallis según corresponda en función de la normalidad de la distribución de los datos, que será comprobada a través del estadístico Shapiro-Wilks.

Los datos cualitativos se analizarán mediante análisis temático. Dos investigadores, realizarán una lectura profunda de las transcripciones y tomarán notas a lo largo del texto, para generar códigos iniciales. Después, el texto será releído, para, en base a estos códigos, buscar y revisar los temas relacionados, para poder contextualizarlos y conceptuarlos. Después, se podrá generar un informe. Otro miembro del equipo cotejará los datos, para asegurar la veracidad de los hallazgos, y mediante la técnica "*peer debriefing technique*" (108), hará conscientes a sus compañeros de sus interpretaciones, con el fin último de limpiar el proceso de asunciones preconcebidas y asunciones erróneas. Se analizarán las frecuencias absolutas y relativas de la información de cada temática. Se evaluará el acuerdo entre evaluadores mediante porcentajes de acuerdo, calculados como el número de unidades temáticas en las que coinciden, dividido entre el total de unidades medidas. Se usará también el índice de Cohen, considerando un valor >0.81 como un nivel de acuerdo aceptable (109).

El análisis de los datos se llevará a cabo con el programa SPSS, versión 26.00.

5. Cronograma y etapas del desarrollo

Tabla 7. Cronograma y plan de trabajo

	**Fechas**
**Diseño del Proyecto**	Diciembre de 2018 - julio de 2019
**Redacción del manuscrito**	Diciembre de 2018 – julio de 2019
**Comité de ética**	Diciembre de 2019 – julio de 2020
**Selección de la muestra**	Agosto de 2021 – septiembre de 2023
**Trabajo de campo**	Junio de 2022 – noviembre de 2023
**Grupos focales**	Octubre de 2022 - diciembre de 2023
**Análisis de los resultados**	Diciembre de 2023 – marzo de 2024
**Difusión de los resultados**	Marzo de 2024 – noviembre de 2024

Debido a la dificultad de reclutar personas con asma, el reclutamiento y la intervención se realizarán de forma paralela. De cada 5 personas reclutadas, se constituirá un grupo que comenzará con la intervención, independientemente de no haber reclutado a toda la muestra.

5.1. Diseño del proyecto

El diseño del proyecto, así como la redacción del manuscrito fueron realizados por Dña. María Vilanova Pereira, con la colaboración de Ana Lista Paz, en el periodo de documentación previa: contexto del estudio e información existente sobre el tema.

5.2 Comité de ética.

Se envío el manuscrito al Comité Ético de Investigación Clínica de Galicia el 4 de noviembre de 2019, y fue aprobado el 20 de julio de 2020. Tras la aceptación, se reenvía para el seguimiento del proyecto y la validación de las adendas en el mismo.

5.3 Selección de la muestra

Una vez obtenida la aprobación para el proyecto, se procederá a la selección de los sujetos que participarán en el proyecto (68 en total; 34 para el grupo de estudio y 34 para el grupo control)

5.4 Desarrollo de la investigación

Una vez seleccionada la muestra, se iniciarán de la mano de Ana Lista Paz, la realización de las mediciones previas, y María Vilanova Pereira, la cual realizará el plan educacional y entrenamiento, la cual ha sido entrenada en marcha nórdica.

5.5 Análisis de los resultados

Concluidas las intervenciones y las evaluaciones, tanto cuantitativas como cualitativas, se procederá al análisis de los resultados, buscando la comprensión conjunta de ambos tipos de datos, mediante un abordaje de método mixto.

5.6 Difusión de los resultados.

Las conclusiones obtenidas serán difundidas a través de los medios descritos posteriormente y de la mano de tres integrantes del equipo investigador: la doctora Ana Lista Paz, el profesor y las fisioterapeutas María Vilanova Pereira y Margarita Barral Fernández.

6. Aspectos éticos-legales

Finalizada la redacción del presente proyecto, éste será enviado al Comité Ético de Investigación Clínica (CEIC) de Galicia, para la aprobación que permita el desarrollo y difusión de los resultados del mismo. Tras su aprobación se reenvía protocolo, con información de seguimiento y validación de una adenda, para un abordaje cualitativo del estudio.

Este ensayo clínico se acoge y cumplirá las normas de Buena Práctica Clínica (ICH) y la Declaración de Helsinki (Brasil 2013)

Se proporcionará información veraz y comprensible a los participantes de este proyecto, acerca de los objetivos del presente estudio, de las pruebas que le vamos a realizar, así como de cuáles son sus contraindicaciones, y las posibles complicaciones que pueden surgir durante las mismas. Esta información se facilita verbalmente y por escrito, junto con el correspondiente consentimiento informado (Anexo 4). Dicho consentimiento informado se elaboró de acuerdo con lo establecido en el artículo 8 de Ley 41/2002, de 14 de noviembre, básica reguladora de la autonomía del paciente y de derechos y obligaciones en materia de información y documentación clínica.

De acuerdo con lo establecido en el artículo 7 de la Ley 41/2002, así como en el Reglamento europeo 2016/679 y en la Ley Orgánica 3/2018 de Protección de Datos Personales y garantía de los derechos digitales, se respetará rigurosamente la confidencialidad de los datos de carácter personal y de salud de los participantes.

En relación con la protección de datos de carácter personal, tal y como se puede ver en cuaderno de recogida de datos (Anexo 5) no se recogerán datos identificativos de los participantes (no se tomarán nombre, apellidos, dirección, teléfono de contacto, DNI, etc). Los sujetos participantes serán pseudonimizados con un número de identificación asignado por la investigadora principal (IP) del estudio (NUMID).

Las grabaciones de audio e imagen obtenidas durante las entrevistas grupales serán guardadas por la investigadora principal en un archivo encriptado en un ordenador de la Facultad de Fisioterapia a la que solo ella y las personas que realicen las transcripciones tendrán acceso. Las transcripciones se realizarán pseudonimizando los datos personales que puedan mencionarse durante las sesiones.

Una vez finalizado el estudio, la información será guardada exclusivamente en formato digital, y custodiada por la delegada de protección de datos da Universidade da Coruña, Luz María Puente Aba, siendo almacenada en una carpeta encriptada, de un ordenador con clave personal a la que solo ella tendrá acceso. Para contactar con la misma: correo electrónico dpg@udc.gal o en teléfono 881 01 16 05 y 881 011 61.

La IP del proyecto y las investigadoras secundarias, María Vilanova Pereira (Col. 3524), Ana Lista Paz (Col. 2122) y Margarita Barral Fernández (Col. 3547), cuentan con un seguro de responsabilidad civil proporcionado por el Colegio de Fisioterapeutas de Galicia (COFIGA). El seguro cuenta con una póliza a raíz de responsabilidad civil (con un tope de 601.012,10€ por siniestro, año y colegiado), que cubre los daños ocasionados por la actividad laboral a terceras personas. Además, la Asociación Española de Fisioterapeutas, de la que el COFIGA es a su vez miembro, define a los fisioterapeutas en sus estatutos como "aquella persona, que estando en posesión del Título Oficial de Fisioterapeuta, puede desarrollar cualquier faceta de su profesión en los ámbitos docente, asistencial, investigador y de gestión, utilizando, para ello, los conocimientos adquiridos en su currículum." Además, en la ORDEN CIN/2135/2008, la cual regula la formación en el grado en Fisioterapia, en el apartado 3, punto 15, la competencia investigadora aparece mencionada como una de las competencias alcanzadas en el grado.

7. Aplicabilidad del estudio

Los resultados de este estudio permitirán, en primer lugar, conocer la efectividad de una terapia alternativa a las convencionales y perfectamente adaptable a la vida de las personas, basada en marcha nórdica, para sujetos con asma. Al tratarse de una actividad desconocida y poco realizada en nuestro contexto geográfico y cultural, nos servirá también para valorar la

aceptación de este ejercicio en la sociedad, pudiendo abrir en un futuro nuevos métodos de entrenamiento y de ejercicio terapéutico. Ejercicio con, presumiblemente, más efectividad, que la marcha convencional, que si está perfectamente aceptada en la sociedad.

Por último, nos permite abrir una nueva línea de investigación en nuestra comunidad: marcha nórdica. De demostrarse los beneficios de la marcha nórdica se podría abordar de una nueva forma a los pacientes con asma. Se trataría de un tratamiento de bajo coste económico para desarrollarlo y permitiría al sistema sanitario ahorrar costes, si disminuyen las exacerbaciones, los medicamentos, las visitas al centro de salud, etc. Se podría analizar por tanto la costo-efectividad de la marcha nórdica en enfermedades respiratorias.

8. Plan de difusión de los resultados

8.1 Congresos

Cada año, el Sindicato de Enfermería (SATSE), realiza un encuentro científico gratuito, destinado a conocer las investigaciones científicas más recientes desarrolladas por profesionales de la Enfermería y Fisioterapia, así como recién graduados. Se intentará presentar como trabajo científico, en el año 2020, se tratará por tanto del "VIII Encuentro científico gallego de Enfermería y Fisioterapia".

Además, se planea presentar los mismos en el Congreso Nacional de la SEPAR en el año 2021. Este congreso se define como un punto de encuentro en el que se muestran los logros de la actividad clínica e investigación de cada año, además de tener un importante papel social, difundiendo entre la ciudadanía los aspectos más importantes de la neumología y cirugía torácica.

Dentro de nuestro interés entraría, del mismo modo, el Congreso de la Asociación Española de Fisioterapeutas, congreso que representa y visibiliza el valor de la Fisioterapia nacional y que sirve de punto de encuentro para profesionales y organizaciones de todo el territorio.

Así mismo, en el congreso internacional de la European Respiratory Society, que se define a sí mismo como un escaparate de excelencia en el campo de la medicina respiratoria.

8.2 Revistas

Se intentará que los resultados se publiquen como artículo de investigación científica en revistas relacionadas con la Fisioterapia y el ámbito sanitario: Archivos de Bronconeumología, órgano de expresión científica de la SEPAR, (factor de impacto 2,979; cuartil 2); en la European Respiratory Journal (factor de impacto 12,242; cuartil 1), Physiotherapy (factor de impacto 1,085; cuartil 1), Chest (factor de impacto 2,591, cuartil 1). Además, para dar a conocer esta modalidad, se publicarán parte de los resultados en la revista órgano de expresión de la AEF, Fisioterapia (SCImago Journal Rank-SJR:0,127).

9. Memoria económica

9.1 Recursos necesarios

En la tabla 8 aparece detallado el material necesario para la realización del estudio, así como el coste del mismo.

Tabla 8. Material necesario.

MATERIAL NECESARIO	UNIDADES	PRECIO (con IVA)
Material inventariable		
Espirómetro Datospir ® 120c	1	1.839,33€
Jeringa de Calibración S3000-3L	1	417,56€
Estación metereologica digital Oregon Scientific ®	1	45,13€
Pinzas nasales	1	1,71€
Báscula y tallímetro	1	140€
Dinamómetro Jamar Dynamometer	1	526,63€
Fonendoscopio electrónico Littman 3200	1	502,73€
Tensiómetro de brazo HYLOGY®	1	26,99€
Pulsioxímetro de dedo Onyx® 9500	1	399,30€
Medidor de flujo pico Asmaplan Vitalograph	68	1.394€
Pulsómetro Polar® H7 beat	5	575,63€
Palos de marcha nórdica Decathlon	12	95,88€
Material fungible		
1 caja de 100 filtros antibacterianos	1	165,77€
Papel térmico impresora espirómetro (10 unidades)	1	21,78€
Otros gastos		
Asistencia a congreso SEPAR	1	784€
Desplazamiento congresos SEPAR	1	250€
Noche de hotel – Congreso SEPAR	3	201€
Dietas de manutención – Congreso SEPAR	3	111€
Asistencia congreso de la AEF	1	190€
Desplazamiento congreso AEF	1	250€
Noche hotel -Congreso AEF	3	201€
Dietas de manutención – Congreso AEF	3	111€
Asistencia congreso ERS	1	625€
Desplazamiento congreso ERS	1	400€
Noche de hotel – Congreso ERS	3	201€
Dietas de manutención – Congreso ERS	3	111€
Proofreading	2	200€
Gastos de imprenta®	1	300€
		Gasto total
		13.048,76€

9.2 Posibles fuentes de financiación

Para la realización y posterior difusión de los resultados obtenidos con este estudio, se buscará financiación a través de diferentes instituciones y organizaciones.

- "Ayudas Respira". La SEPAR destina cada año 20.000€ en ayudas, para profesionales enfermeros o fisioterapeutas, para la presentación de comunicaciones en el Congreso Nacional SEPAR. Estas ayudas cubren los gastos de desplazamiento y alojamiento de los interesados, para facilitar su asistencia a dichos congresos.
- Ayudas para la investigación del *COFIGA. El COFIGA* destina 5.000€ en ayudas, a repartir entre un total de 5 investigadores. Concedida.
- Becas de ayuda a la investigación de SEPAR. Cada año SEPAR abre una convocatoria de becas dotas de 18.000 euros en el caso de Fisioterapia, a la que podremos optar con este proyecto. Concedida.
- Becas becario de SEPAR. Van dirigidas fundamentalmente a socios de SEPAR que hayan formalizado recientemente su formación clínica como residentes en Neumología o Cirugía Torácica, o para Diplomados Universitarios y Graduados en Enfermería y Fisioterapia, que deseen formarse en el campo de la investigación respiratoria, desarrollando un proyecto de investigación científica.

10. Medios disponibles y equipo investigador para la realización de la propuesta

Para la realización de este proyecto contamos con el apoyo de la Facultad de Fisioterapia de la Universidade da Coruña, la cual pondrá a disposición del equipo investigador un espacio adecuado para la realización de las mediciones de campo, en el cual se garantiza la intimidad de los/as participantes. Para la realización del entrenamiento con marcha nórdica se aprovecharán los espacios verdes y azules de la ciudad de A Coruña.

10.1 Experiencia del equipo investigador

En cuanto a los recursos humanos disponibles, la IP del proyecto, la fisioterapeuta María Vilanova Pereira, quien realizará el entrenamiento con marcha nórdica. Esta profesional ha realizado una estancia en la Universidad de Ciencias Aplicadas de Savonia (Kuopio), sita en Finlandia, cuna de la marcha nórdica. Durante la misma ha realizado un entrenamiento como instructora de marcha nórdica en la Suomen Latu. María Vilanova Pereira será también la encargada de realizar el plan de educación para la salud de los participantes, para lo cual será previamente entrenada por la investigadora colaboradora del estudio, Ana Lista Paz.

Además, la profesora Ana Lista Paz, tiene una dilatada experiencia en la realización de estudios de investigación que implican medición de variables de función pulmonar, fuerza y resistencia de los músculos respiratorios y tolerancia al ejercicio. Ejemplos de ello son los estudios: *"Análisis comparativo de la función pulmonar en sujetos con y sin*

hemiplejía/hemiparesia crónica", el cual ya ha dado producción científica, y el estudio multicéntrico titulado *"Determinación de los valores de referencia de las presiones respiratorias máximas y de la presión nasal en inhalación máxima en población española adulta sana",* actualmente en desarrollo.

El reclutamiento de los sujetos de estudio será facilitado por los jefes de servicio de Neumología y Alergología del CHUAC, y los pacientes de la consulta de Neumología del HM Modelo. La aleatorización a los grupos será realizada por la Fisioterapeuta Margarita Barral Fernández.

Los sujetos deberán leer la hoja de información al paciente (Anexo 3) y firmar un consentimiento informado, disponible en el Anexo 4, después de ser informados por su médico acerca del objetivo y de la intervención del estudio, así como de las mediciones que se llevarán a cabo para la realización del mismo.

Por último, en el análisis estadístico de los datos contaremos con la participación del profesor Alejandro Quintela del Río, experto en estadística y con amplia experiencia en análisis de datos biosanitarios.

10.2 Recursos materiales disponibles

La Facultad de Fisioterapia de la UDC pone a disposición de este proyecto el siguiente material:

- Espirómetro Datospir®120C,
- Jeringa de calibración S3000-3L,
- Báscula y tallímetro.
- Dinamómetro Jamar Dynamometer.
- Fonendoscopio electrónico Littman 3200.
- Pulsioxímetro
- Tensiómetro de brazo
- Relojes Polar®
- Material fungible.

No será necesario que el equipo investigador afronte dichos gastos, por tanto, se reduce el presupuesto de material (sin tener en cuenta los gastos de difusión) de 8.415,63€ a 2.119,38€.

Por último, la Facultad de Fisioterapia de la Universidade da Coruña, tiene capacidad de acceso a la información científica a través de las diferentes bases de datos de Ciencias de la

Salud, y tiene acceso al paquete estadístico necesario para realizar el análisis estadístico de los datos.

11. Bibliografía

1. What is Nordic Walking? [Internet]. International Nordic Walking Federation. 2019. Disponible en: http://www.inwa-nordicwalking.com/what-is-nordic-walking/

2. Skórkowska-Telichowska K, Kropielnicka K, Bulińska K, Pilch U, Woźniewski M, Szuba A, et al. Nordic walking in the second half of life. Aging Clinical and Experimental Research. 2016;28(6):1035-46.

3. Bombieri F, Schena F, Pellegrini B, Barone P, Tinazzi M, Erro R. Walking on four limbs: A systematic review of Nordic Walking in Parkinson disease. Parkinsonism & Related Disorders. 2017;38:8-12.

4. Breyer M-K, Breyer-Kohansal R, Funk G-C, Dornhofer N, Spruit MA, Wouters EF, et al. Nordic Walking improves daily physical activities in COPD: a randomised controlled trial. Respiratory Research [Internet]. 2010 [citado 1 de diciembre de 2018];11(1). Disponible en: http://respiratory-research.biomedcentral.com/articles/10.1186/1465-9921-11-112

5. World Record-Nordic Walking. Nordic Walking history. [Internet]. World Record-Nordic Walking. 2018. Disponible en: https://www.wr-nw.com/nordic-walking-history.html

6. International Nordic Walking Asociation. History of Nordic Walking [Internet]. International Nordic Walking Association. 2018. Disponible en: http://www.inwa-nordicwalking.com/inwa-history/

7. International Nordic Walking Asociation Spain. INWA SPAIN [Internet]. International Nordic Walking Association (INWA) Spain. [citado 12 de diciembre de 2018]. Disponible en: http://www.inwaspain.com/

8. Original Nordic Walking Federation. Original pole length recommendation [Internet]. Original Nordic Walking Federation. 2018. Disponible en: https://onwf.org/original-pole-length-recommendation-for-nordic-walking/

9. World Record-Nordic Walking. ONW AND INWA TECHNIQUES [Internet]. World Record-Nordic Walking. 2018. Disponible en: https://www.wr-nw.com/onw-inwa-techniques.html

10. World Record-Nordic Walking. FITTREK TECHNIQUE [Internet]. World Record-Nordic Walking. 2018. Disponible en: https://www.wr-nw.com/fittrek-technique.html

11. Pellegrini B, Peyré-Tartaruga LA, Zoppirolli C, Bortolan L, Savoldelli A, Minetti AE, et al. Mechanical energy patterns in nordic walking: comparisons with conventional walking. Gait & Posture. 2017;51:234-8.

12. Pellegrini B, Peyré-Tartaruga LA, Zoppirolli C, Bortolan L, Bacchi E, Figard-Fabre H, et al. Exploring Muscle Activation during Nordic Walking: A Comparison between Conventional and Uphill Walking. Carrier D, editor. PLOS ONE. 2015;10(9):e0138906.

13. Shim J, Kwon H, Kim H, Kim B, Jung J. Comparison of the Effects of Walking with and without Nordic Pole on Upper Extremity and Lower Extremity Muscle Activation. Journal of Physical Therapy Science. 2013;25(12):1553-6.

14. Saulicz M, Saulicz E, Myśliwiec A, Wolny T, Linek P, Knapik A, et al. Effect of a 4-week Nordic walking training on the physical fitness and self-assessment of the quality of health of women of the perimenopausal age. Menopausal Review. 2015;2:105-11.

15. Park H-S, Lee S-N, Sung D-H, Choi H-S, Kwon TD, Park GD. The Effect of Power Nordic Walking on Spine Deformation and Visual Analog Pain Scale in Elderly Women with Low Back Pain. Journal of Physical Therapy Science. 2014;26(11):1809-12.

16. Park SD, Yu SH. The effects of Nordic and general walking on depression disorder patients' depression, sleep, and body composition. Journal of Physical Therapy Science. 2015;27(8):2481-5.

17. Vílchez Barrera ME, Calvo-Arencibia A. Evidencia científica de la marcha nórdica en Fisioterapia: revisión bibliográfica. Fisioterapia. 2016;38(5):251-64.

18. Fritz T, Caidahl K, Krook A, Lundström P, Mashili F, Osler M, et al. Effects of Nordic walking on cardiovascular risk factors in overweight individuals with type 2 diabetes, impaired or normal glucose tolerance: Randomized Control Nordic Walking Study. Diabetes/Metabolism Research and Reviews. 2013;29(1):25-32.

19. Venojärvi M, Korkmaz A, Wasenius N, Manderoos S, Heinonen OJ, Lindholm H, et al. 12 Weeks' aerobic and resistance training without dietary intervention did not influence oxidative stress but aerobic training decreased atherogenic index in middle-aged men with impaired glucose regulation. Food and Chemical Toxicology. 2013;61:127-35.

20. Gram B, Christensen R, Christiansen C, Gram J. Effects of Nordic Walking and Exercise in Type 2 Diabetes Mellitus: A Randomized Controlled Trial. Clin J Sport Med. 2010;20(5):7.

21. Fritz T, Caidahl K, Osler M, Östenson CG, Zierath JR, Wändell P. Effects of Nordic walking on health-related quality of life in overweight individuals with Type 2 diabetes mellitus, impaired or normal glucose tolerance: Nordic walking-effects on quality of life in overweight individuals. Diabetic Medicine. 2011;28(11):1362-72.

22. Wiklund P, Alen M, Munukka E, Cheng SM, Yu B, Pekkala S, et al. Metabolic response to 6-week aerobic exercise training and dieting in previously sedentary overweight and obese pre-menopausal women: A randomized trial. Journal of Sport and Health Science. 2014;3(3):217-24.

23. Sentinelli F, La Cava V, Serpe R, Boi A, Incani M, Manconi E, et al. Positive effects of Nordic Walking on anthropometric and metabolic variables in women with type 2 diabetes mellitus. Science & Sports. 2015;30(1):25-32.

24. Hartvigsen J, Morsø L, Bendix T, Manniche C. Supervised and non-supervised Nordic walking in the treatment of chronic low back pain: a single blind randomized clinical trial. BMC Musculoskeletal Disorders [Internet]. 2010 [citado 28 de abril de 2019];11(1). Disponible en: https://bmcmusculoskeletdisord.biomedcentral.com/articles/10.1186/1471-2474-11-30

25. Gerhard B, Manuela P, Helga T, Erwin G. Work-site health promotion of frequent computer users: Comparing selected interventions. Work. 2013;(3):233-41.

26. Mannerkorpi K, Nordeman L, Cider Å, Jonsson G. Does moderate-to-high intensity Nordic walking improve functional capacity and pain in fibromyalgia? A prospective randomized controlled trial. Arthritis Research & Therapy. 2010;12(5):R189.

27. Spafford C, Oakley C, Beard JD. Randomized clinical trial comparing Nordic pole walking and a standard home exercise programme in patients with intermittent claudication: Nordic pole walking *versus* standard home exercise programme in patients with intermittent claudication. British Journal of Surgery. 2014;101(7):760-7.

28. Collins EG, O'Connell S, McBurney C, Jelinek C, Butler J, Reda D, et al. Comparison of Walking With Poles and Traditional Walking for Peripheral Arterial Disease Rehabilitation: Journal of Cardiopulmonary Rehabilitation and Prevention. 2012;32(4):210-8.

29. Keast M-L, Slovinec D'Angelo ME, Nelson CRM, Turcotte SE, McDonnell LA, Nadler RE, et al. Randomized Trial of Nordic Walking in Patients With Moderate to Severe Heart Failure. Canadian Journal of Cardiology. 2013;29(11):1470-6.

30. Homma D, Jigami H, Sato N. Effects of Nordic walking on pelvis motion and muscle activities around the hip joints of adults with hip osteoarthritis. Journal of Physical Therapy Science. 2016;28(4):1213-8.

31. Müllerova H, Agusti A, Erqou S, Mapel DW. Cardiovascular Comorbidity in COPD. Chest. 2013;144(4):1163-78.

32. Cugusi L, Manca A, Yeo TJ, Bassareo PP, Mercuro G, Kaski JC. Nordic walking for individuals with cardiovascular disease: A systematic review and meta-analysis of randomized controlled trials. European Journal of Preventive Cardiology. 2017;24(18):1938-55.

33. Wilk et al. Assessment of the selected physiological effects of Nordic Walking performed as a part of a physical exercise program during the second phase of rehabilitation after a myocardial infarction. Rehabilitacja Medyczna. 2005;(9):20-5.

34. Kocur P, Deskur-Śmielecka E, Wilk M, Dylewicz P. Effects of Nordic Walking training on exercise capacity and fitness in men participating in early, short-term inpatient cardiac rehabilitation after an acute coronary syndrome — a controlled trial. Clinical Rehabilitation. 2009;23(11):995-1004.

35. Shin J-H, Kim C-B, Choi J-D. Effects of trunk rotation induced treadmill gait training on gait of stroke patients: a randomized controlled trial. Journal of Physical Therapy Science. 2015;27(4):1215-7.

36. Kang T-W, Lee J-H, Cynn H-S. Six-Week Nordic Treadmill Training Compared with Treadmill Training on Balance, Gait, and Activities of Daily Living for Stroke Patients: A Randomized Controlled Trial. Journal of Stroke and Cerebrovascular Diseases. 2016;25(4):848-56.

37. Latosik E, Zubrzycki IZ, Ossowski Z, Bojke O, Clarke A, Wiacek M, et al. Physiological Responses Associated with Nordic-Walking Training in Systolic Hypertensive Postmenopausal Women. Journal of Human Kinetics. 2014;43(1):185-90.

38. Lejczak A, Josiak K, Węgrzynowska - Teodorczyk K, Rudzińska E, Jankowska E, Banasiak W, et al. Nordic Walking May Safely Increase the Intensity of Exercise Training in Healthy Subjects and in Patients with Chronic Heart Failure. Advances in Clinical and Experimental Medicine. 2016;25(1):145-9.

39. Beyer SE, Sanghvi MM, Aung N, Hosking A, Cooper JA, Paiva JM, et al. Prospective association between handgrip strength and cardiac structure and function in UK adults. Abete P, editor. PLOS ONE. 2018;13(3):e0193124.

40. Piotrowicz E, Zieliński T, Bodalski R, Rywik T, Dobraszkiewicz-Wasilewska B, Sobieszczańska-Małek M, et al. Home-based telemonitored Nordic walking training is well accepted, safe, effective and has high adherence among heart failure patients, including those with cardiovascular implantable electronic devices: a randomised controlled study. European Journal of Preventive Cardiology. 2015;22(11):1368-77.

41. Bulińska K, Kropielnicka K, Jasiński T, Wojcieszczyk-Latos J, Pilch U, Dąbrowska G, et al. Nordic pole walking improves walking capacity in patients with intermittent claudication: a randomized controlled trial. Disability and Rehabilitation. 2016;38(13):1318-24.

42. Langbein WE, Collins EG, Orebaugh C, Maloney C, Williams KJ, Littooy FN, et al. Increasing exercise tolerance of persons limited by claudication pain using polestriding. Journal of Vascular Surgery. 2002;35(5):887-93.

43. Collins EG, Edwin Langbein W, Orebaugh C, Bammert C, Hanson K, Reda D, et al. PoleStriding Exercise and Vitamin E for Management of Peripheral Vascular Disease: Medicine & Science in Sports & Exercise. 2003;35(3):384-93.

44. Collins EG, Langbein WE, Orebaugh C, Bammert C, Hanson K, Reda D, et al. Cardiovascular Training Effect Associated With Polestriding Exercise in Patients With Peripheral Arterial Disease: The Journal of Cardiovascular Nursing. 2005;20(3):177-85.

45. Collins EG, McBurney C, Butler J, Jelinek C, O'Connell S, Fritschi C, et al. The Effects of Walking or Walking-with-Poles Training on Tissue Oxygenation in Patients with Peripheral Arterial Disease. International Journal of Vascular Medicine. 2012;2012:1-8.

46. Oakley C, Spafford C, Beard JD. A Three Month Home Exercise Programme Augmented with Nordic Poles for Patients with Intermittent Claudication Enhances Quality of Life and Continues to Improve Walking Distance and Compliance After One Year. European Journal of Vascular and Endovascular Surgery. 2017;53(5):704-9.

47. Kropielnicka K, Dziubek W, Bulińska K, Stefańska M, Wojcieszczyk-Latos J, Jasiński R, et al. Influence of the Physical Training on Muscle Function and Walking Distance in Symptomatic Peripheral Arterial Disease in Elderly. BioMed Research International. 2018;2018:1-16.

48. Girold S, Rousseau J, Le Gal M, Coudeyre E, Le Henaff J. Nordic walking versus walking without poles for rehabilitation with cardiovascular disease: Randomized controlled trial. Annals of Physical and Rehabilitation Medicine. 2017;60(4):223-9.

49. Rinaldo N, Bacchi E, Coratella G, Vitali F, Milanese C, Rossi A, et al. Effects of Combined Aerobic-Strength Training vs Fitness Education Program in COPD Patients. International Journal of Sports Medicine. 2017;38(13):1001-8.

50. Casaburi R, ZuWallack R. Pulmonary Rehabilitation for Management of Chronic Obstructive Pulmonary Disease. New England Journal of Medicine. 2009;360(13):1329-35.

51. Ochman M, Maruszewski M, Latos M, Jastrzębski D, Wojarski J, Karolak W, et al. Nordic Walking in Pulmonary Rehabilitation of Patients Referred for Lung Transplantation. Transplantation Proceedings. 2018;50(7):2059-63.

52. Jastrzebski D, Ochman M, Ziora D, Labus L, Kowalski K, Wyrwol J, et al. Pulmonary Rehabilitation in Patients Referred for Lung Transplantation. En: Pokorski M, editor. Respiratory Regulation - Clinical Advances [Internet]. Dordrecht: Springer Netherlands; 2013 [citado 27 de abril de 2019]. p. 19-25. Disponible en: http://www.springerlink.com/index/10.1007/978-94-007-4546-9_3

53. Sociedad Española de Medicos Generales y de familia. GEMA 4.4: Guía española para el manejo del asma.

54. Gastaldi AC, Paredi P, Talwar A, Meah S, Barnes PJ, Usmani OS. Oscillating Positive Expiratory Pressure on Respiratory Resistance in Chronic Obstructive Pulmonary Disease With a Small Amount of Secretion: A Randomized Clinical Trial. Medicine. 2015;94(42):e1845.

55. França-Pinto A, Mendes FAR, de Carvalho-Pinto RM, Agondi RC, Cukier A, Stelmach R, et al. Aerobic training decreases bronchial hyperresponsiveness and systemic inflammation in patients with moderate or severe asthma: a randomised controlled trial. Thorax. 2015;70(8):732-9.

56. Sodhi C, Singh S, Bery A. Assessment of the Quality of Life in Patients with Bronchial Asthma, Before and After Yoga: a Randomised Trial. 2014;13(1):6.

57. Lorenc AB, Wang Y, Madge SL, Hu X, Mian AM, Robinson N. Meditative Movement for Respiratory Function: A Systematic Review. Respiratory Care. 2014;59(3):427-40.

58. Cramer H, Posadzki P, Dobos G, Langhorst J. Yoga for asthma: a systematic review and meta-analysis. Annals of Allergy, Asthma & Immunology. 2014;112(6):503-510.e5.

59. Raghavendra P, Shetty P, Shetty S, Manjunath NK, Saoji AA. Effect of high-frequency yoga breathing on pulmonary functions in patients with asthma. Annals of Allergy, Asthma & Immunology. 2016;117(5):550-1.

60. Meyer A, Günther S, Volmer T, Taube K, Baumann HJ. A 12-month, moderate-intensity exercise training program improves fitness and quality of life in adults with asthma: a controlled trial. BMC Pulmonary Medicine [Internet]. diciembre de 2015 [citado 28 de diciembre de 2018];15(1). Disponible en: http://bmcpulmmed.biomedcentral.com/articles/10.1186/s12890-015-0053-8

61. Bruurs MLJ, van der Giessen LJ, Moed H. The effectiveness of physiotherapy in patients with asthma: A systematic review of the literature. Respiratory Medicine. 2013;107(4):483-94.

62. Eichenberger PA, Diener SN, Kofmehl R, Spengler CM. Effects of Exercise Training on Airway Hyperreactivity in Asthma: A Systematic Review and Meta-Analysis. Sports Medicine. 2013;43(11):1157-70.

63. Wanrooij VH, Willeboordse M, Dompeling E, van de Kant KD. Exercise training in children with asthma: a systematic review. British Journal of Sports Medicine. 2014;48(13):1024-31.

64. Mendes FAR, Gonçalves RC, Nunes MPT, Saraiva-Romanholo BM, Cukier A, Stelmach R, et al. Effects of Aerobic Training on Psychosocial Morbidity and Symptoms in Patients With Asthma. Chest. 2010;138(2):331-7.

65. Basaran S, Guler-Uysal F, Ergen N, Seydaoglu G, Bingol-Karakoç G, Ufuk Altintas D. EFFECTS OF PHYSICAL EXERCISE ON QUALITY OF LIFE, EXERCISE CAPACITY AND PULMONARY FUNCTION IN CHILDREN WITH ASTHMA. Journal of Rehabilitation Medicine. 2006;38(2):130-5.

66. Turner S, Eastwood P, Cook A, Jenkins S. Improvements in Symptoms and Quality of Life following Exercise Training in Older Adults with Moderate/Severe Persistent Asthma. Respiration. 2011;81(4):302-10.

67. Flapper BCT, Duiverman EJ, Gerritsen J, Postema K, van der Schans CP. Happiness to be gained in paediatric asthma care. European Respiratory Journal. 2008;32(6):1555-62.

68. Fanelli A, Cabral ALB, Neder JA, Martins MA, Carvalho CRF. Exercise Training on Disease Control and Quality of Life in Asthmatic Children: Medicine & Science in Sports & Exercise. 2007;39(9):1474-80.

69. Ram, FSF, Robinson SM, Black PN, Picot J. Physical training for asthma. 2005;19;(4):CD001116.

70. Mendes FAR, Almeida FM, Cukier A, Stelmach R, Jacob-Filho W, Martins MA, et al. Effects of Aerobic Training on Airway Inflammation in Asthmatic Patients: Medicine & Science in Sports & Exercise. 2011;43(2):197-203.

71. Shaw BS, Shaw I. Pulmonary Function and Abdominal and Thoracic Kinematic Changes Following Aerobic and Inspiratory Resistive Diaphragmatic Breathing Training in Asthmatics. Lung. 2011;189(2):131-9.

72. Wang J-S, Hung W-P. The effects of a swimming intervention for children with asthma. Respirology. 2009;14(6):838-42.

73. Arbillaga-Etxarri A, Gimeno-Santos E, Barberan-Garcia A, Balcells E, Benet M, Borrell E, et al. Long-term efficacy and effectiveness of a behavioural and community-based exercise intervention (Urban Training) to increase physical activity in patients with COPD: a randomised controlled trial. European Respiratory Journal. 2018;52(4):1800063.

74. Guía de Práctica Clínica para el Diagnóstico y Tratamiento de Pacientes con Enfermedad Pulmonar Obstructiva Crónica (EPOC) - Guía Española de la EPOC (GesEPOC). Archivos de Bronconeumología. 2012;48:2-58.

75. Fletcher GF, Ades PA, Kligfield P, Arena R, Balady GJ, Bittner VA, et al. Exercise Standards for Testing and Training: A Scientific Statement From the American Heart Association. Circulation. 2013;128(8):873-934.

76. ATS Statement: Guidelines for the Six-Minute Walk Test. Am J Respir Crit Care Med. 2002;Vol 166:pp 111-117.

77. García-Río F, Calle M, Burgos F, Casan P, del Campo F, Galdiz JB, et al. Espirometría. Archivos de Bronconeumología. 2013;49(9):388-401.

78. Bohannon RW, Crouch R. Minimal clinically important difference for change in 6-minute walk test distance of adults with pathology: a systematic review: Systematic review of MCID in 6MWT. Journal of Evaluation in Clinical Practice. 2017;23(2):377-81.

79. Coelho CM, Reboredo MM, Valle FM, Malaguti C, Campos LA, Nascimento LM, et al. Effects of an unsupervised pedometer-based physical activity program on daily steps of adults with moderate to severe asthma: a randomized controlled trial. Journal of Sports Sciences. 2018;36(10):1186-93.

80. Kim Y, Park I, Kang M. Convergent validity of the International Physical Activity Questionnaire (IPAQ): meta-analysis. Public Health Nutrition. 2013;16(03):440-52.

81. Brazier JE, Harper R, Jones NM, O'Cathain A, Thomas KJ, Usherwood T, et al. Validating the SF-36 health survey questionnaire: new outcome measure for primary care. BMJ. 1992;305(6846):160-4.

82. Vilagut G, Ferrer M, Rajmil L, Rebollo P, Permanyer-Miralda G, Quintana JM, et al. El Cuestionario de Salud SF-36 español: una década de experiencia y nuevos desarrollos. Gaceta Sanitaria. 2005;19(2):135-50.

83. Wyrwich KW, Tierney WM, Babu AN, Kroenke K, Wolinsky FD. A Comparison of Clinically Important Differences in Health-Related Quality of Life for Patients with Chronic Lung Disease, Asthma, or Heart Disease: Clinically Important Differences on the SF-36. Health Services Research. 2005;40(2):577-92.

84. Juniper EF, Buist AS, Cox FM, Ferrie PJ, King DR. Validation of a Standardized Version of the Asthma Quality of Life Questionnaire. Chest. 1999;115(5):1265-70.

85. Perpiñá M, de Diego A, Compte L, Belloch A, Pascual LM. Calidad de vida en el asma: validación del cuestionario AQLQ para su utilización en población española. Archivos de Bronconeumología. 1995;31(5):211-8.

86. Jones PW. Interpreting thresholds for a clinically significant change in health status in asthma and COPD. European Respiratory Journal. 2002;19(3):398-404.

87. Schatz M, Sorkness CA, Li JT, Marcus P, Murray JJ, Nathan RA, et al. Asthma Control Test: Reliability, validity, and responsiveness in patients not previously followed by asthma specialists. Journal of Allergy and Clinical Immunology. 2006;117(3):549-56.

88. Schatz M, Kosinski M, Yarlas AS, Hanlon J, Watson ME, Jhingran P. The minimally important difference of the Asthma Control Test. Journal of Allergy and Clinical Immunology. 2009;124(4):719-723.e1.

89. Vega JM, Badia X, Badiola C, López-Viña A, Olaguíbel JM, Picado C, et al. Validation of the Spanish Version of the Asthma Control Test (ACT). Journal of Asthma. 2007;44(10):867-72.

90. Plaza V, Fernández-Rodríguez C, Melero C, Cosío BG, Entrenas LM, de Llano LP, et al. Validation of the 'Test of the Adherence to Inhalers' (TAI) for Asthma and COPD Patients. Journal of Aerosol Medicine and Pulmonary Drug Delivery. 2016;29(2):142-52.

91. Kendrick KR, Baxi SC, Smith RM. Usefulness of the modified 0-10 Borg scale in assessing the degree of dyspnea in patients with COPD and asthma. Journal of Emergency Nursing. 2000;26(3):0216-22.

92. Hajiro T, Nishimura K, Tsukino M, Ikeda A, Koyama H, Izumi T. Analysis of Clinical Methods Used to Evaluate Dyspnea in Patients with Chronic Obstructive Pulmonary Disease. 1998;158:5.

93. Mulholland A, Ainsworth A, Pillarisetti N. Tools in Asthma Evaluation and Management: When and How to Use Them? The Indian Journal of Pediatrics. 2018;85(8):651-7.

94. National Institutes of Health, National Heart, Lung and Blood Institute. Global Strategy for Asthma management and prevention. [Internet]. Disponible en: www.ginasthma.org

95. Cuesta-Vargas A, Hilgenkamp T. Reference Values of Grip Strength Measured with a Jamar Dynamometer in 1526 Adults with Intellectual Disabilities and Compared to Adults without Intellectual Disability. Gallup AC, editor. PLOS ONE. 2015;10(6):e0129585.

96. Stark T, Walker B, Phillips JK, Fejer R, Beck R. Hand-held Dynamometry Correlation With the Gold Standard Isokinetic Dynamometry: A Systematic Review. PM&R. 2011;3(5):472-9.

97. Norman K, Stobäus N, Gonzalez MC, Schulzke J-D, Pirlich M. Hand grip strength: Outcome predictor and marker of nutritional status. Clinical Nutrition. 2011;30(2):135-42.

98. IPAQ. Guidelines for Data Processing and Analysis of the International Physical Activity Questionnaire (IPAQ) – Short and Long Forms [Internet]. 2005. Disponible en: http://www.academia.edu/5346814/Guidelines_for_Data_Processing_and_Analysis_of_t he_International_Physical_Activity_Questionnaire_IPAQ_Short_and_Long_Forms_Co nte nts.

99. Miller MR. Standardisation of spirometry. European Respiratory Journal. 2005;26(2):319-38.

100. Kimura Y, Takahashi M, Wada F, Hachisuka K. Differences in the Peak Cough Flow among Stroke Patients With and Without Dysphagia. Journal of UOEH. 2013;35(1):9-16.

101. Calaf N. Medición de las presiones respiratorias máximas. En: Comité científico SEPAR. Manual SEPAR de procedimientos: procedimientos de evaluación de la función pulmonar II [Internet]. Sociedad Española de Neumología y Cirugía Torácica. 2004;122-44.

102. Sergio Sancho Marín. Análisis comparativo de la medición de las presiones respiratorias máximas según dos protocolos distintos. 2018;

103. Morales P, Sanchis J, Cordero PJ, Díez JL. Presiones respiratorias estáticas máximas en adultos. Valores de referencia de la población caucasica mediterránea. Archivos de Bronconeumología. 1997;(33):213-9.

104. Barreiro E, Bustamante V, Cejudo P, Gáldiz JB, de Lucas P, et al. Normativa SEPAR sobre disfunción muscular de los pacientes con Enfermedad Pulmonar Obstructiva Crónica. Archivos de Bronconeumología. 51(8).

105. Mathiowetz V et al. Grip and pinch strength: normative data for adults. Arch Phys Med Rehabil. 1985;66(2):69-74.

106. Nici L, Donner C, Wouters E, Zuwallack R, Ambrosino N, Bourbeau J, et al. American Thoracic Society/European Respiratory Society Statement on Pulmonary Rehabilitation. American Journal of Respiratory and Critical Care Medicine. 2006;173(12):1390-413.

107. Garber CE, Blissmer B, Deschenes MR, Franklin BA, Lamonte MJ, Lee I-M, et al. Quantity and Quality of Exercise for Developing and Maintaining Cardiorespiratory, Musculoskeletal, and Neuromotor Fitness in Apparently Healthy Adults: Guidance for Prescribing Exercise. Medicine & Science in Sports & Exercise. 2011;43(7):1334-59.

108. Lincoln Y, Guba E. Naturalistic inquiry. Beverly Hills, CA:Sega.

109. McHugh. Interrater reliability: the kappa statistic. Biochem Med. 2012;22(3):276-82.

12. Anexos

Anexo 1: Contraindicaciones para la participación en el estudio

12.2.1 Contraindicaciones para la realización de la prueba de 6MWT

Según la guía ATS/ERS (2).

Absolutas:

- Angina inestable en el último mes.
- Infarto agudo de miocardio en el último mes.

Relativas:

- Frecuencia cardíaca en reposo mayor de 120.
- Presión sanguínea sistólica mayor de 180mmHg.
- Presión sanguínea diastólica mayor de 100mmHg.

12.2.2 Contraindicaciones para la realización de una espirometría

Según la SEPAR (3).

Absolutas:

- Inestabilidad hemodinámica.
- Embolismo pulmonar (hasta anticoagulación adecuada).
- Neumotórax reciente (2 semanas tras la reexpansión).
- Hemoptisis aguda.
- Infecciones respiratorias activas (tuberculosis, norovirus, influenza).
- Infarto de miocardio reciente (7 días).
- Angina inestable.
- Aneurisma de la aorta torácica que ha crecido o de gran tamaño (>6 cm).
- Hipertensión intracraneal.
- Desprendimiento agudo de retina.

Relativa:

- Niños menores de 5-6 años.
- Pacientes confusos o demenciados.
- Cirugía abdominal o torácica reciente.
- Cirugía cerebral, ocular u otorrinolaringológica reciente.

- Diarrea o vómitos agudos, estados nauseosos.
- Crisis hipertensiva.
- Problemas bucodentales o faciales que impidan o dificulten la colocación y sujeción de la boquilla.

12.2.3 Clasificación de las exacerbaciones del asma

	Crisis leve	Crisis moderada-grave	Parada respiratoria
Disnea	Leve	Moderada-intensa	Muy intensa
Habla	Párrafos	Frases-palabras	
Frecuencia respiratoria (x')	Aumentada	>20-30	
Frecuencia cardiaca (x)	<100	>100-120	Bradicardia
Uso musculatura accesoria	Ausente	Presente	Movimiento paradójico toracoabdominal
Sibilancias	Presentes	Presentes	Silencio auscultatorio
Nivel de conciencia	Normal	Normal	Disminuido
Pulso paradójico	Ausente	>12-25 mmHg	Ausencia (fatiga muscular)
FEV1 o PEF (valores de referencia)	>70%	<70%	
SaO2 (%)	>95%	90-95%	<90%
PaO2 mmHg	Normal	80-60	<60
PaCO2	<40	>40	>40
FEV: volumen espiratorio forzado en el primer segundo; PEF: flujo espiratorio máximo; x': por minuto; SaO2: saturación de oxihemoglobina; PaO2: presión arterial de oxígeno; PaCO2: presión arterial de anhídrino carbónico.			

(4)

1. ATS Statement: Guidelines for the Six-Minute Walk Test. Am J Respir Crit Care Med. 2002;Vol 166:pp 111–117.

2. García-Río F, Calle M, Burgos F, Casan P, del Campo F, Galdiz JB, et al. Espirometría. Archivos de Bronconeumología. 2013;49(9):388-401.

3. Sociedad Española de Médicos Generales y de familia. GEMA 4.4: Guía española para el manejo del asma.

Anexo 2. Diario del paciente

**Día: __/__/____**			
**Nº de pasos**			
		**Sí**	**No**
**Síntomas**	Disnea (Sensación de ahogo respiratorio)		
	Del 0 al 10 (Escala de Borg):		
	Expectoración		
	Tos		
	Sibilancias (pitido)		
		**Sí**	**No**
PEF (L/min)			
Toma de medicamentos			
¿Cuáles?			
Visita a urgencia			

Diría que hoy me siento:

☹ 😐 ☺

La palabra que mejor define mi estado de ánimo hoy es:

____

Actividades que he realizado hoy:

Observaciones:

Si tienes cualquier duda puedes contactar con el equipo investigador escribiendonos un SMS o Whatsapp, llamando o enviandonos un correo a través de los siguientes medios:

Teléfono: +34 628 10 10 24 *Correo electrónico: mn.udc2019@gmail.com*

Anexo 3. Hoja de información del paciente (castellano y gallego)

TÍTULO DEL ESTUDIO: COMPARACIÓN DE UN PROGRAMA DE MARCHA NÓRDICA FRENTE AL USO EXCLUSIVO DE UN PLAN EDUCACIONAL EN PACIENTES CON ASMA.

INVESTIGADOR: MARÍA VILANOVA PEREIRA

CENTRO: UNIVERSIDADE DA CORUÑA

Este documento tiene por objeto ofrecerle información sobre un **estudio de investigación** en el que se le invita a participar. Este estudio fue aprobado por el Comité Ético de Investigación Clínica de Galicia

Si decide participar en el mismo, debe recibir información personalizada del investigador, **leer antes este documento** y hacer todas las preguntas que precise para comprender los detalles sobre el mismo. Si así lo desea puede llevar el documento, consultarlo con otras personas y tomar el tiempo necesario para decidir si participa o no.

La participación en este estudio es completamente **voluntaria**. Ud. puede decidir no participar o, se acepta hacerlo, cambiar de parecer retirando el consentimiento en cualquier momento sin dar explicaciones. Le aseguramos que esta decisión no afectará a la relación con con los profesionales sanitarios que le atienden ni a la asistencia sanitaria a la que Ud. tiene derecho.

¿Cuál es la finalidad del estudio?

Comprobar si un entrenamiento basado en marcha nórdica presenta beneficios en pacientes con asma, con respecto a un plan educacional.

¿Por qué me ofrecen participar a mí?

Ud. es invitado a participar porque aparece en las alergología del Complejo Hospitalario Universitario de A Coruña y del Hospital HM Modelo de A Coruña, y además cumple los criterios de inclusión de este estudio: usted tiene más de 18 años y está diagnosticado de asma. Además, desea participar en este estudio y tiene la capacidad de firmar el consentimiento informado. Es por esto por lo que su médico especialista le propone en consulta formar parte de este proyecto.

¿En qué consiste mi participación?

En función del grupo al que pertenezca, tendrá que realizar un entrenamiento de marcha nórdica y/o seguir una serie de recomendaciones, y cumplimentar un diario que sirve como hoja de registro, así como someterse a una serie de pruebas sencillas y no invasivas

En primer lugar, será sometido a una serie de mediciones, que volveremos a realizar al final del periodo de tratamiento y a los 3 y 6 meses después de finalizar. Estas mediciones se realizarán, en la Facultad de Fisioterapia de la Universidade da Coruña y tendrán una duración aproximada de 2 horas y media. Incluyen:

- Test de 6 minutos marcha.
- Cuestionario sobre nivel de actividad física diaria: *International Physical Activity Questionnaire (IPAQ).*
- Cuestionario general de calidad de vida *SF-36*.
- Cuestionario de calidad de vida en personas diagnosticadas con enfermedades respiratorias: *Saint George's Respiratory Questionnaire*.
- Cuestionario de calidad de vida para personas con asma: *Asthma Quality of Life Questionnaire*.
- Cuestionario sobre el control de asma y síntomas: *Asthma Control Test*.
- Medición de la disnea según la Escala de Borg y la escala modificada del *Medical Research Council.*
- Espirometría forzada.
- Medición de la fuerza resistencia de la musculatura, realizada con dinamómetro, valorando la fuerza de agarre.
- Además de esto, le pediremos que diariamente anote un diario que le será proporcionado los siguientes datos:
    - Número de pasos dados cada día. Esta información la obtendrá de una aplicación en el móvil en la que será instruido.
    - Flujo espiratorio pico. Realizará una medición diaria con un medidor portátil que le será proporcionado.
- Toma de medicamentos.
  Visitas a urgencias.
- Adherencia

El programa incluye un plan educacional, que consiste en 2 clases de 2 horas cada una en el que se le aportará información acerca de su enfermedad y recomendaciones y consejos sobre sus cuidados. Estas clases tendrán lugar en el mismo sitio de las pruebas, la Facultad de Fisioterapia de la Universidade da Coruña.

En caso de pertenecer al grupo que realizará un entrenamiento de marcha nórdica, será sometido durante 8 semanas a un entrenamiento de 60 minutos al día, 3 días a la semana. El lugar de entrenamiento será urbano, es decir, en entornos de la ciudad de A Coruña. Este

incluirá ejercicios de movilidad articular, estiramientos y relajación, además de una caminata de 30 minutos realizando marcha nórdica.

Por lo tanto, su participación tendrá una duración total estimada de 10 semanas, y 3 visitas posteriores de seguimiento.

Al grupo de marcha nórdica, una vez finalizada la intervención, se le citará también para una entrevista en grupo, de tipo grupos focales, en la que conversará con sus compañero/as de grupo sobre diferentes aspectos planteados por el moderador de la reunión, en relación a su experiencia y satisfacción con la actividad propuesta de marcha nórdica. Durante esta entrevista grupal, tendrá total libertad a la hora de dar sus respuestas, y será necesaria su interactuación con sus compañero/as. Será gravado/a en vídeo y audio, y sus respuestas serán transcritas para su posterior análisis. A esta grabación y transcripción tendrán acceso solo las investigadoras de este proyecto, y no serán difundidos en ningún caso. Serán guardados en una carpeta encriptada, en un ordenador con contraseña que permanecerá en la Facultad de Fisioterapia de la Universidad de la Coruña, hasta finalizar con el análisis de los datos. En este mismo documento, en el apartado “Información referente a sus datos”, tiene información sobre como contactar con la delegada de protección de datos de la Universidad de la Coruña, en caso de querer realizar alguna consulta, reclamación o ejercicio de derechos al respecto de este material.

Para la realización de este estudio, dadas las mediciones que se realizarán, deberá contar con un dispositivo móvil con conexión a internet, en la que le pediremos que descargue una aplicación encargada de contar los pasos que da en su día a día. Usted será instruído en como descargar la aplicación, su funcionamiento básico y que datos nos interesan de ella.

¿Qué molestias o inconvenientes tiene mi participación?

Su participación no implica molestias adicionales a las de realizar ejercicio físico moderado.

Remarcar que vamos a utilizar pruebas de esfuerzo de tipo submáximo (test de 6 minutos marcha) como herramienta evaluadora, lo que no supone ningún riesgo para usted, ya que son pruebas en las que no se alcanzan el máximo de sus capacidades (se calcula un margen amplio hasta el límite de las mismas), están limitadas por sus propios síntomas y sensaciones, y sus signos vitales (frecuencia cardiaca y saturación de oxígeno) estarán continuamente monitorizados. No son más peligrosas que la práctica deportiva general, y además su duración no superará los 10-15 minutos. Bien es cierto, que como esfuerzo y actividad física que son, usted se puede notar cansado después de la realización de las mismas.

Tanto la investigadora principal del proyecto, Dña. María Vilanova Pereira (Col. 3524) como la investigadora secundaria, la Dra. Ana Lista Paz (Col. 2122), y la persona que realiza las evaluaciones, junto con la Dra. Lista, la investigadora colaboradora Margarita Barral Fernández (Col. 3547), están formadas en las técnicas de recogida de información y de entrenamiento utilizadas, y además cuentan con un seguro de responsabilidad civil proporcionado por el Colegio Oficial de Fisioterapeutas de Galicia, que aunque como hemos dicho, el riesgo que se presenta es el mismo que el de cualquier actividad física o deportiva, cubre los daños de cualquier incidente, inconveniente o accidente que pueda surgir, también a terceros, por el desarrollo de su profesión en el ámbito investigador.

En caso de necesitarlo, puede tomar su medicación de rescate ante exacerbaciones del asma como de costumbre.

¿Obtendré algún beneficio por participar?

No se espera que Ud. obtenga beneficio directo por participar en el estudio. La investigación pretende descubrir aspectos desconocidos o poco claros sobre.la marcha nórdica en pacientes con asma.

Esta podrá ser de utilidad en un futuro para otras personas.

¿Recibiré la información que se obtenga del estudio?

Si Ud. lo desea, se le facilitará un resumen de los resultados del estudio.

¿Se publicarán los resultados de este estudio?

Los resultados de este estudio serán remitidos a publicaciones científicas para su difusión, pero no se transmitirá ningún dato que permita la identificación de los participantes.

Información referente a sus datos:

La obtención, tratamiento, conservación, comunicación y cesión de sus datos se hará conforme a lo dispuesto en el Reglamento General de Protección de Datos (Reglamento UE 2016-679 del Parlamento Europeo y del Consejo, de 27 de abril de 2016) y la normativa española sobre protección de datos de carácter personal vigente.

Los datos necesarios para llevar a cabo este estudio serán recogidos y conservados de modo*:*

- **Seudonimizados (Codificados),** la seudonimización es el tratamiento de datos personales de manera tal que no pueden atribuirse a un/a interesado/a sin que se use información adicional. En este estudo solamente el equipo investigador conocerá el código que permitirá saber su identidad.

La normativa que regula el tratamiento de datos de personas le otorga el derecho a acceder a sus datos, oponerse, corregirlos, cancelarlos, limitar su tratamiento, restringir o solicitar la supresión de los mismos. También puede solicitar una copia de éstos o que ésta sea remitida a un tercero (derecho de portabilidad).

Para ejercer estos derechos, se puede dirigir al Delegado de Protección de Datos del centro, a través del correo electrónico dpd@udc.gal. La persoa encargada de la protección de datos del centro es Luz María Puente Aba, dirección postal Rúa da Maestranza,9, 15001, A Coruña. El número de teléfono de contacto es el 881 011 605, o el 88 101 161.

Así mismo, Ud. tiene derecho a interponer una reclamación ante la Agencia Española de Protección de datos cuando considere que alguno de sus derechos no haya sido respetado.

Únicamente el equipo investigador y las autoridades sanitarias, que tienen el deber de guardar la confidencialidad, tendrán acceso a todos los datos recogidos por el estudio. Se podrá transmitir a terceros información que no pueda ser identificada.

10 años después de la finalización del estudio, los datos recogidos serán eliminados o guardados anónimos para su uso en futuras investigaciones según lo que Ud. escoja en la hoja de firma del consentimiento.

¿Existen intereses económicos en este estudio?

El investigador no recibirá retribución específica por la dedicación al estudio.

Ud. no será retribuido por participar. Es posible que de los resultados del estudio se deriven productos comerciales o patentes; en este caso, Ud. no participará de los beneficios económicos originados.

¿Cómo contactar con el equipo investigador de este estudio?

Ud. puede contactar con María Vilanova Pereira, en el teléfono +34 628101024 o en el correo electrónico marchanordicafisio@gmail.com, o con su médico de referencia, investigador colaborador de este estudio, en los números detallados anteriormente en este documento (aparatado de información referente a sus datos)

Muchas gracias por su colaboración

TÍTULO DO ESTUDO: COMPARACIÓN DUN PROGRAMA DE MARCHA NÓRDICA FRONTE AO USO EXCLUSIVO DUN PLAN EDUCACIONAL EN PACIENTES CON ASMA.

INVESTIGADORA: MARÍA VILANOVA PEREIRA.

CENTRO: UNIVERSIDADE DA CORUÑA

Este documento ten por obxecto ofrecerlle información sobre un estudo de investigación no que se lle invita a participar. Este estudo foi aprobado polo Comité Ético de Investigación Clínica de Galicia.

Se decide participar no mesmo, debe recibir información persoalizada do investigador, ler este documento e facer todas as preguntas que precise para comprender os detalles sobre o mesmo. Se así o desexa, pode levarse o documento, consultalo con outras persoas e tomar o tempo necesario para decidir se participa ou non.

A participación neste estudo é completamente voluntaria. Vostede pode decidir non participar ou, se acepta facelo, cambiar de parecer retirando o consentimento en calquera momento sen dar explicacións. Asegurámoslle que esta decisión non afectará á relación cos profesionais sanitarios que o atenden nin á asistencia sanitaria á que vostede ten dereito.

Cal é a finalidade do estudo?

Comprobar se un adestramento baseado en marcha nórdica presenta beneficios en pacientes con asma, respecto dun plan educacional.

Por qué me ofrecen participar a min?

Vostede é invitado a participar porque aparece nas listas de Alergoloxía do Complexo Hospitalario Universitario de A Coruña, e do Hospital HM Modelo de A Coruña, e ademáis cumpre os criterios de inclusión deste estudio: vostede ten máis de 18 anos e esta diagnosticado de asma. Ademais desexa participar neste estudo e ten a capacidade de firmar o consentimento informado. É por isto que o seu médico especialista lle propón en consulta a posibilidade de participar neste proxecto.

En que consiste a miña participación?

En función do grupo ao que pertenza, terá que realizar un adestramento de marcha nórdica e/ou seguir unha serie de recomendacións, e cumprimentar un diario que serve como folla de rexistro, así como someterse a unha serie de probas sinxelas e non invasivas.

En primeiro lugar, será sometido a unha serie de medicións, que volveremos a realizar ao final do período de tratamento e aos 3 e 6 meses despois de finalizar. Estas mediciones realizaránse na Facultade de Fisioterapia da Universidade da Coruña e terán unha duración aproximada de 2 horas e media. Incluén:

- Test de 6 minutos marcha.
- Cuestionario sobre nivel de actividade física diaria: *International Physical Activity Questionnaire (IPAQ).*
- Cuestionario xeral de calidade de vida *SF-36*.
- Cuestionario de calidade de vida para persoas con asma: *Asthma Quality of Life Questionnaire*.
- Cuestionario sobre o control da asma e síntomas: *Asthma Control Test*.
- Medición da disnea segundo a Escala de Borg e a escala modificada del *Medical Research Council.*
- Espirometría forzada.
- Medición de la PIM y la PEM (se usa para avaliar la forza de la musculatura respiratoria)
- Medición da forza resistencia de la musculatura, realizada con dinamómetro, valorando a forza de agarre.
- Además de isto, le pediremos que diariamente anote un diario que le será proporcionado os seguintes datos:
    - Número de pasos dados cada día. Esta información obteraa dunha aplicación do móbil na que será instruído.
    - Fluxo espiratorio pico. Realizará una medición diaria con un medidor portátil que lle será proporcionado.
- Toma de medicamentos.
  Visitas a urxencias.
- Adherencia

O programa inclúe un plan educacional, que consiste en 2 clases de 2 horas cada unha na que se lle aportará información acerca da súa enfermidade e recomendacións e consellos sobre os seus coidados. Estas clases terán lugar no mesmo sitio das probas, a Facultade de Fisioterapia da Universidade da Coruña.

En caso de pertencer ao grupo que realizará un adestramento de marcha nórdica, será sometido durante 8 semanas a un adestramento de 60 minutos ao día, 3 días á semana. O lugar de adestramentos será urbano, é dicir, en contornas da cidade da Coruña. Este incluirá

exercicios de mobilidade articular, estiramentos, relaxación, ademais de unha camiñata de 30 minutos realizando marcha nórdica.

Polo tanto, a súa participación terá unha duración total estimada de 10 semanas e 3 visitas posteriores de seguimento.

Ao grupo de marcha nórdica, unha vez finalizada a intervención, citarase tamén para unha entrevista en grupo, de tipo grupos focais, na que conversará cos seus compañeiro/as de grupo sobre diferentes aspectos prantexados polo moderador da reunión, en relación á súa experiencia e satisfacción coa actividade proposta de marcha nórdica. Durante esta entrevista grupal, terá total liberdade á hora de dar as súas respostas, e será necesario a interactuación cos seus compañeiro/as. Será gravado/a en vídeo e audio, e as súas respostas serán transcritas para a súa análise posterior. A esta gravación e transcrición terán acceso só as investigadoras deste proxecto, e non serán difundidos en ningún caso. Serán gardados nunha carpeta encriptada, nun ordenador con contrasinal, que permanecerá na Facultade de Fisioterapia da Universidade da Coruña, ata finalizar coa análise de datos. Neste mismo documento, no apartado "información referente aos seus datos", ten información sobre como contactar coa delegada de protección de datos da Universidade da Coruña, en caso de querer realizar algunha consulta, reclamación ou exercicio de dereitos ao respecto deste material.

Para a realización deste estudo, dadas as medicións que se realizarán, deberá contar cun dispositivo móbil con conexión a internet, na que lle pediremos que descargue unha aplicación encargada de contar os pasos que da no seu día a día. Vostede será instruído en como descargar a aplicación, o seu funcionamento básico e que datos nos interesan dela.

¿Que molestias e inconvintes ten a miña participación?

A súa participación non implica molestias adicionais ás de practicar exercicio físico moderado.

Remarcar que vamos a utilizar probas de esforzo de tipo submáximo (test de 6 minutos marcha) como ferramenta evaluadora, o que non supón ningún risco para vostede, xa que son probas nas que non se alcanza o máximo das súas capacidades (calculase unha marxe ampla ata o límite das mesmas), e están limitadas polos seus propios síntomas e sensacións, e os seus signos vitais (frecuencia cardíaca e saturación de osíxeno) estarán continuamente monitorizados. Non son máis perigosas que a práctica deportiva xeral, e ademais, a súa duración non superará os 10-15 minutos. Ben e certo, que como esforzo e actividade física que son, vostede pódese notar cansado despois da realización das mesmas.

Tanto a investigadora principal do proxecto, a María Vilanova Pereira (Col. 3524), como a investigadora secundaria, Dra. Ana Lista Paz (Col. 2122) e a persoa que realiza as avaliacións, xunto a Dra. Lista, a investigadora colaboradora Margarita Barral Fernández (Col. 3547) contan cun seguro de responsabilidade civil proporcionado polo Colexio Oficial de Fisioterapeutas de Galicia, que aínda que o risco que se presenta é o mesmo que o de calquera actividade física ou deportiva, cubre os danos de calquera incidente, inconvinte ou accidente que poida xurdir, tamén a terceiros, polo desenrolo da súa profesión no ámbito investigador.

Obterei algún beneficio por participar?

Non se espera que vostede obteña beneficio directo por participar no estudo. A investigación pretende descubrir aspectos descoñecidos ou pouco claros sobre a marcha nórdica en pacientes con asma.

Esta información poderá ser de utilidade en un futuro para outras persoas.

Publicaranse os resultados deste estudo?

Os resultados deste estudo serán remitidos a publicacións científicas para a súa difusión, pero non se transmitirá ningún dato que permita a identificación dos participantes.

Información referente aos seus datos:

A obtención, tratamento, conservación, comunicación e cesión dos seus datos farase conforme ao disposto no Reglamento General de Protección de Datos (Reglamento UE 2016-679 do Parlamento Europeo e do Consejo, de 27 de abril de 2016) e a normativa española sobre a protección de datos de carácter persoal vixente.

Os datos necesarios para levar a cabo este estudo serán recollidos e conservados de modo:

- Seudonomizados (Codificados), a seudonimización é o tratamento de datos persoais de maneira tal que non poidan atribuírse a unha persoa interesada sen que se use información adicional. Neste estudo soamente o equipo investigador coñecerá o código que permitirá saber a súa identidade.

A normativa que regula o tratamento de datos persoais outórgalle o dereito a acceder aos seus datos, opoñerse, correxilos, cancelalos, limitar o seu tratamento, restrinxir ou solicitar a supresión dos mesmos. Tamén pode solicitar unha copia destes ou que esta sexa remitida a un terceiro (dereito de portabilidade).

Para exercer estes dereitos, vostede pode dirixirse ao Delegado de Protección de Datos do centro a través do correo electrónico dpd@udc.gal. A persoa encargada da protección de datos do centro é Luz María Puente Aba, enderezo postal Rúa da Maestranza,9, 15001, A Coruña. O número de teléfono de contacto é o 881 011 605, ou o 88 101 161.

Así mesmo, vostede ten dereito a interpor unha reclamación ante a Axencia Española de Protección de datos cando considere que algún dos seus dereitos non foi respectado.

Unicamente o equipo investigador e as autoridades sanitarias, que teñen o deber de gardar a confidencialidade, terán acceso a todos os datos recollidos polo estudo. Poderase transmitir a terceiros información que non poida ser identificada.

10 anos despois da finalización do estudo, os datos recollidos serán eliminados ou gardados anónimos para o seu uso en futuras investigacións segundo o que vostede escolla na súa folla de firma do consentimento.

Existen intereses económicos neste estudo?

O investigador non recibirá retribución específica pola dedicación ao estudo.

Vostede non será retribuído por participar. É posible que dos resultados do estudo se deriven productos comerciais ou patentes: neste caso, vostede non participará dos beneficios económicos orixinados.

Como contactar co equipo investigador deste estudo?

Vostede pode contactar con María Vilanova Pereira, no correo electrónico marchanordicafisio@gmail.com ou co seu médico de referencia, investigador colaborador deste estudo, da forma detallada anteriormente neste documento (apartado información referente aos seus datos)

Moitas grazas pola súa colaboración.

Anexo 4. Consentimiento informado (castellano y gallego)

DOCUMENTO DE CONSENTIMIENTO INFORMADO PARA LA PARTICIPACIÓN EN UN ESTUDIO DE INVESTIGACIÓN

Título: **Comparación de un programa de marcha nórdica frente al uso exclusivo de un plan de educación en pacientes con asma.**

Don/doña ____,
mayor de edad, con DNI __________ y domicilio en ___________________________________
_______________________________,

DECLARO que

He sido informado/a de las características del estudio	Sí ☐	No ☐
He leído la hoja de información que me han entregado	Sí ☐	No ☐
He podido realizar las observaciones o preguntas y me fueron aclaradas las dudas	Sí ☐	No ☐
He entendido las explicaciones que se me han facilitado y en que cosiste mi participación en el estudio	Sí ☐	No ☐
Sé cómo y a quién me tengo que dirigir para realizar preguntas sobre el estudio en el presente y en el futuro	Sí ☐	No ☐
He sido informado de los riesgos asociados a mi participación	Sí ☐	No ☐
No cumplo ninguno de los criterios de exclusión como participante y sé que si esto cambia en algún momento debo hacérselo saber al equipo de investigación	Sí ☐	No ☐
Confirmo que mi participación es voluntaria	Sí ☐	No ☐
Entiendo que puedo revocar el consentimiento en cualquier momento sin tener que dar explicaciones y sin que esto repercuta negativamente en mi persona	Sí ☐	No ☐

CONSIENTO

Participar en estudio	Sí ☐	No ☐
Que se utilicen los datos facilitados para la investigación	Sí ☐	No ☐
Que se utilicen los datos facilitados en publicaciones científicas	Sí ☐	No ☐
Que se utilicen los datos facilitados en reuniones y congresos	Sí ☐	No ☐
Que se utilicen los datos facilitados para la docencia	Sí ☐	No ☐
Que se grabe en audio para la obtención de los datos	Sí ☐	No ☐
Que se grabe en vídeo para la obtención de los datos	Sí ☐	No ☐
Que se utilicen citas textuales de mis intervenciones, sin identificar, en publicaciones	Sí ☐	No ☐
Que se conserven los datos de forma anónima al finalizar el estudio para su uso en futuras investigaciones	Sí ☐	No ☐
Que se conserven los datos codificados al finalizar el estudio para su uso en futuras investigaciones siempre que garanticen el tratamiento de los datos conforme a este consentimiento	Sí ☐	No ☐
Que contacten conmigo para obtener nuevos datos	Sí ☐	No ☐

SOLICITO

Acceder a los resultados generales del estudio	Sí ☐	No ☐
Acceder a la información sobre mi derivada del estudio	Sí ☐	No ☐
Acceder a los artículos científicos una vez fuesen publicados	Sí ☐	No ☐
La destrucción de mis datos una vez finalizado el estudio	Sí ☐	No ☐
Incluir las siguientes restricciones al uso de mis datos:		

Y en prueba de conformidad, firmo el presente documento en el lugar y fecha indicados a continuación.

____________, _______ de _____________________ de ___ ___.

Nombre y apellidos del/de la participante:<br><br>Firma:	*Nombre y apellidos del/de la investigador/a principal*:<br><br>Firma:

DOCUMENTO DE CONSENTIMENTO INFORMADO PARA A PARTICIPACIÓN NUN ESTUDIO DE INVESTIGACIÓN

Título: **Comparación de un programa de marcha nórdica fronte ao uso exclusivo dun plan de educación en pacientes con asma.**

Don/dona ____,
maior de idade, con DNI __________ e domicilio en ____________________________________ ________________________________,

DECLARO que

Fun informado/a de as características do estudo	Si ☐	Non ☐
Lin a folla de información que me entregaron	Si ☐	Non ☐
Puiden realizar observacións ou preguntas e fóronme aclaradas as dúbidas	Si ☐	Non ☐
Comprendín as explicacións que se me facilitaron e en que consiste a miña participación no estudo	Si ☐	Non ☐
Sei como e a quen me dirixir para realizar preguntas sobre o estudo no presente ou no futuro	Si ☐	Non ☐
Fun informado/a dos riscos asociados á miña participación	Si ☐	Non ☐
Son coñecedor/a de que non cumpro ningún dos criterios de exclusión como participante e que se isto cambiase ao longo do estudo débollo facer saber ao equipo de investigación	Si ☐	Non ☐
Confirmo que a miña participación é voluntaria	Si ☐	Non ☐
Comprendo que podo revogar o consentimento en calquera momento sen ter que dar explicacións e sen que repercuta negativamente na miña persoa	Si ☐	Non ☐

CONSINTO

Participar no estudo	Si ☐	Non ☐
Que se utilicen os datos facilitados para a investigación	Si ☐	Non ☐
Que se utilicen os datos facilitados en publicacións científicas	Si ☐	Non ☐
Que se utilicen os datos facilitados en reunións e congresos	Si ☐	Non ☐
Que se utilicen os datos facilitados para a docencia	Si ☐	Non ☐
Que se grave en audio para a obtención dos datos	Si ☐	Non ☐
Que se grave en vídeo para a obtención dos datos	Si ☐	Non ☐
Que se utilicen citas textuais das miñas intervencións, sen identificar, en publicacións	Si ☐	Non ☐
Que se conserven os datos de forma anónima ao finalizar o estudo para o seu uso en futuras investigacións	Si ☐	Non ☐
Que se conserven os datos codificados ao finalizar o estudo para o seu uso en futuras investigacións sempre que garantan o tratamento dos datos conforme a este consentimento	Si ☐	Non ☐
Que contacten comigo para obter novos datos	Si ☐	Non ☐

SOLICITO

Acceder aos resultados xerais do estudo	Si ☐	Non ☐
Acceder á información sobre min derivada do estudo	Si ☐	Non ☐
Acceder aos artigos científicos unha vez fosen publicados	Si ☐	Non ☐
A destrución dos meus datos unha vez finalizado o estudo	Si ☐	Non ☐
Incluír as seguintes restricións ao uso dos meus datos:		

E en proba de conformidade, asino o presente documento no lugar e na data que se indican a continuación.

____________, _______ de _____________________ de ___ ___.

Nome e apelidos do/da participante:<br><br><br>Sinatura:	*Nome e apelidos do/a investigador/a principal*:<br><br><br>Sinatura:

Anexo 5. Cuaderno de recogida de datos del paciente

Datos sociodemográficos

Sexo: Hombre Mujer

Mes y año de nacimiento: ____/________

Edad (años): ___

Nacionalidad: ____

Situación laboral actual:

- □ Activo/a
- □ Inactivo/a o en desempleo
- □ De baja
- □ Jubilado
- □ Otros (especificar): ____

En caso de estar inactivo/desempleado/de baja o jubilado, ¿desde hace cuánto tiempo?:

Años: ___ Meses:____

Profesión: ____

Hábitos tóxicos (tabaquismo)

- ¿Ha fumado alguna vez?
  - □ Sí, diariamente *(exclusión)*
  - □ Sí, de forma ocasional *(exclusión).*
  - □ No fumo actualmente, pero he fumado antes.
  - □ No, nunca he fumado.

Para las personas que no fuman actualmente, pero sí lo han hecho en el pasado (exfumadores)

- ¿A qué edad comenzó usted a fumar? ____ años.
- ¿Cúanto tiempo hace que dejo usted de fumar? ____años y ____meses.
  - □ Por tanto, el tiempo total que ha sido fumador han sido: ____años y ____meses.
- ¿Qué tipo y cantidad de tabaco fumaba por término medio al día?
  - □ Número de cigarrillos/día: ____

- □ Número de pipas/día: ___ (1 pipa/semana= 0.14 pipa/día).
- □ Número de puros/día: ___
- □ Número de puritos/día: ____

- Actualmente, ¿está expuesto al humo? (fumador pasivo):
  - □ Sí ¿Durante cuánto tiempo? (horas/día): ____
  - □ No
- Cálculo del índice tabáquico (años fumados x cigarrillos fumados/20)

Antecedentes respiratorios

- ¿Padece o ha padecido alguna de las siguientes enfermedades respiratorias?

	SÍ	NO
EPOC		
Alergia crónica		
Tipo:		
Fibrosis quística		
Fibrosis pulmonar		
Bronquiectasias		
Tuberculosis		
Derrame pleural		
¿Cuándo?		
Neumotórax		
¿Cuándo?:		
Otras:		

- ¿Ha sufrido algún trasplante de pulmón o resección pulmonar?
  - □ Sí.
  - □ No.
- ¿Sufre usted neumonías/infecciones respiratorias con frecuencia?
  - □ Sí.
    - ¿Con qué frecuencia? (nº veces/año):_____
    - ¿Cuándo fue la última? (en meses): _____
  - □ No.

Antecedentes patológicos

- ¿Padece o ha padecido alguna de las siguientes enfermedades?

	SÍ	NO
**HTA**		
¿Está controlada?		
**Infarto agudo de miocardio**		
¿Cuándo? ______ años ______ meses		
**Angina de pecho**		
**Trasplante de corazón**		
¿Cuándo? ______ años ______ meses		
**Otras enfermedades cardiovasculares:**		
**Diabetes**		
**Varices en las piernas**		
**Artrosis, artritis o reumatismos**		
**Incontinencia urinaria/fecal**		
**Tumores malignos**		
**Cirugía torácica/abdominal**		
¿Cuándo?		
¿Motivo?		
**Enfermedades neuromusculares**		
**Dolor de espalda crónico (lumbar)**		
**Dolor de espalda crónico (cervical)**		
**Úlcera de estómago o duodeno**		
**Colesterol alto**		
**Cataratas**		
**Problemas crónicos de la piel**		
**Estreñimiento crónico**		
**Cirrosis, disfunción hepática**		
**Depresión**		
**Ansiedad crónica**		
**Otros problemas mentales**		
**Ictus (embolia, infarto cerebral, hemorragia cerebral)**		
**Migraña o dolor de cabeza frecuente**		
**Hemorroides**		
**Osteoporosis**		
**Problemas de tiroides**		
**Problemas de riñón**		
**Problemas de próstata (solo hombres)**		
**Problemas de periodo menopáusicos (solo mujeres)**		
**Lesiones o disfunciones permanentes causadas por algún accidente**		
**Ninguna de las anteriores**		
¿Alguna enfermerdad o alteración musculoesquelética que le impida o dificulte la marcha?		
Otras enfermedades de base.		

CONSUMO FARMACOLÓGICO

¿Qué medicamentos toma actualmente?

- □ Medicinas para el catarro, gripe, garganta
- □ Medicinas para los bronquios (inhaladores)

¿Cuáles? ____

- □ Medicinas para el dolor
- □ Antibióticos
- □ Tranquilizantes, relajantes (Exclusión si son relajantes musculares y los toma de manera habitual), pastillas para dormir
- □ Antidepresivos (Exclusión si son barbitúricos)
- □ Medicamentos para la alergia
- □ Medicinas para el reuma
- □ Medicinas para el corazón
- □ Medicinas para la tensión arterial
- □ Medicinas para el estómago y/o las alteraciones digestivas
- □ Píldoras para no quedar embarazada (solo para mujeres)
- □ Hormonas para la menopausia (solo para mujeres)
- □ Medicamentos para adelgazar
- □ Medicamentos para bajar el colesterol
- □ Medicamentos para la diabetes
- □ Medicamentos para el tiroides
- □ Ninguna de las anteriores
- □ Otros:____

EXPLORACIÓN FÍSICA

**MEDIDAS ANTROPOMÉTRICAS**		
Masa (kg)	Talla (cm)	IMC (kg/m^2)
**SIGNOS VITALES**		
FC (lpm):		
$SatO_2$(%):		

Lo medimos antes del 6MWT

DINAMOMETRÍA

MANO IZQUIERDA		MANO DERECHA	
1º		1º	
2º		2º	
3º		3º	

ESPIROMETRÍA

Parámetro	Valor obtenido	Valor de referencia
FVC		
FEV1		
FVC/FEV1		
FEF25-75%		

FUERZA DE LA MUSCULATURA RESPIRATORIA

Parámetro	Valor obtenido (mmHg)	Valor de referencia
PIM		
PEM		

OBSERVACIONES

____

____

____

DISCONTINUACIÓN EN EL ESTUDIO

Completar si durante la entrevista, la exploración física o durante las pruebas, se detecta que el sujeto no cumple alguno de los criterios de exclusión, o si el paciente decide que no quiere continuar con el programa en el transcurso del mismo.

Razón de la exlcusión:

____

____

____

Anexo 6. Registro del test de 6 minutos marcha

Sujeto:

PRUEBA:			
Tramo	Distancia recorrida (m)	Tiempo (s)	FC (lpm)
1	30		
2	60		
3	90		
4	120		
5	150		
6	180		
7	210		
8	240		
9	270		
10	300		
11	330		
12	360		
13	390		
14	420		
15	450		
16	480		
17	510		
18	540		
19	570		
20	600		
21	630		
22	660		
23	690		
24	720		
Distancia adicional			m
**Distancia total**			m
Número de paradas			-
Tiempo total de las paradas			s

Paradas	Tiempo (s)	Motivo

1’	Lo está haciendo muy bien, faltan 5 minutos
2’	Perfecto, continúe así, faltan 4 minutos
3’	Está en la mitad de la prueba, lo está haciendo muy bien
4’	Perfecto, continue así, faltan 2 minutos
5’	Lo está haciendo muy bien, falta 1 minuto
5’45”	Deberá detenerse con la indicación: PARE
6’	PARE

Fecha y hora:

**VALORES BASALES**		
FC		Lpm
$SatO_2$		%
FR		rpm
Disnea		Borg
Fatiga MMII		Borg
**VALORES FINALES**		
FC		lpm
$SatO_2$		%
FR		Rpm
Disnea		Borg
Fatiga MMII		Borg
**VALORES DE RECUPERACIÓN**		
Tiempo	FC (lpm)	SatO2 (%)
1’		
2’		
3’		
4’		
5’		

**MOTIVOS PARA SUSPENDER EL 6MWT**	
	Dolor torácico
	Disnea intolerable
	Calambres en MMII
	Diaforesis inexplicable
	Palidez, sensación de mareo
	$SatO_2$%<85%
	Querer parar
	Dolor torácico
CONTRAINDICACIONES PARA EL 6MWT	
	Ángor intestable (<1 mes)
	IAM (<1 mes)
	FC en reposo >120 lpm
	FC en reposo <40 lpm + TAS <110mmHg
	HTA no controlada o TAS >180mmHg en reposo
	TAD > 100mmHg e reposo (cuando hay síntomas de HTA)

Anexo 7. Cuestionario Internacional de Actividad Física. Versión corta.

Estamos interesados en saber acerca de la clase de actividad física que la gente hace como parte de su vida diaria. Las preguntas se referirán acerca del tiempo que usted utilizó siendo físicamente activo(a) **en los últimos 7 días**. Por favor responda a cada pregunta aún si usted no se considera una persona activa.

Por favor, piense en aquellas actividades que usted hace como parte del trabajo, en el jardín y en la casa, para ir de un sitio a otro, y en su tiempo libre de descanso, ejercicio o deporte.

*Piense acerca de todas aquellas **actividades vigorosas** que usted realizó en **los últimos 7 días**. Actividades vigorosas son las que requieren un esfuerzo físico fuerte y le hacen respirar mucho más fuerte de lo normal. Piense solamente en esas actividades que usted hizo **por lo menos 10 minutos continuos.***

1. Durante los **últimos 7 días**, ¿Cuántos días realizó usted **actividades físicas vigorosas** como levantar objetos pesados, excavar, aeróbicos, o pedalear rápido en bicicleta?

_____ días por semana

□ Ninguna actividad física vigorosa → **Pase a la pregunta 3**

2. ¿Cuánto tiempo en total dedicó a realizar actividades físicas vigorosas en uno de esos días que las realizó?

_____ horas por día

_____ minutos por día

□ No sabe/No está seguro(a)

*Piense acerca de todas aquellas **actividades moderadas** que usted realizó en los **últimos 7 días.** Actividades moderadas son aquellas que requieren un esfuerzo físico moderado y le hace respirar algo más fuerte de lo normal. Piense solamente en esas actividades que usted hizo **por lo menos 10 minutos continuos.***

3. Durante los últimos 7 días, ¿Cuántos días hizo usted actividades físicas moderadas tal como cargar objetos livianos, pedalear en bicicleta a paso regular, o jugar dobles de tenis? No incluya caminatas.

_____ días por semana

- □ Ninguna actividad física moderada → . **Pase a la pregunta 5**

4. Usualmente, ¿Cuánto tiempo dedica usted en uno de esos días a hacer actividades físicas **moderadas**?

_____ **horas por día**
_____ **minutos por día**

- □ No sabe/No está seguro(a)

*Piense acerca del tiempo que usted dedicó a caminar en los **últimos 7 días**. Esto incluye trabajo en la casa, caminatas para ir de un sitio a otro, o cualquier otra caminata que usted hizo únicamente por recreación, deporte, ejercicio, o placer.*

5. Durante los **últimos 7 días**, ¿Cuántos días caminó usted por al menos 10 minutos continuos?

_____ **días por semana**

- □ No caminó → **Pase a la pregunta 7**

6. Usualmente, ¿cuánto tiempo gastó usted en uno de esos días **caminando**?

_____ **horas por día**
_____ **minutos por día**

- □ No sabe/No está seguro(a)

*La última pregunta se refiere al tiempo que usted permaneció **sentado(a)** en la semana en los **últimos 7 días**. Incluya el tiempo sentado(a) en el trabajo, la casa, estudiando, y en su tiempo libre. Esto puede incluir tiempo sentado(a) en un escritorio, visitando amigos(as), leyendo o permanecer sentado(a) o acostado(a) mirando televisión.*

7. Durante los **últimos 7 días**, ¿Cuánto tiempo permaneció **sentado(a)** en un **día entre semana?**

_____ **horas por día**
_____ **minutos por día**

- □ No sabe/No está seguro(a)

Anexo 8. Cuestionario de calidad de vida *Short Form- Health Survey* 36

1. ¿En general, usted diría que su salud es?

☐ Excelente$_1$ ☐Muy buena$_2$ ☐ Buena$_3$ ☐ Regular$_4$ ☐ Mala$_5$

2. ¿Cómo diría usted que es su salud actual, comparada con la de hace un año?

☐ Excelente$_1$ ☐ Muy buena$_2$ ☐ Buena$_3$ ☐ Regular$_4$ ☐ Mala$_5$

3. Las siguientes preguntas se refieren a actividades o cosas que usted podría hacer en un **día normal**. Su salud actual, ¿le limita para hacer estas actividades o cosas? Si es así, ¿cuánto?

	Sí, me limita mucho	Sí, me limita un poco	No, no me limita nada
a. Esfuerzos intensos, tales como correr, levantar objetos pesados, o participar en deportes agotadores	1	2	3
b. Esfuerzos moderados, como mover una mesa, pasar la aspiradora, jugar a los bolos o caminar más de 1 hora	1	2	3
c. Coger o llevar la bolsa de la compra	1	2	3
d. Subir varios pisos por la escalera	1	2	3
e. Subir un solo piso por la escalera	1	2	3
f. Agacharse o arrodillarse	1	2	3
g. Caminar un kilómetro o más	1	2	3
h. Caminar varios centenares de metros	1	2	3
i. Caminar unos 100 metros	1	2	3
j. Bañarse o vestirse por si mismo	1	2	3

4. Durante las últimas 4 semanas, ¿con qué frecuencia ha tenido alguno de los siguientes problemas en su trabajo o en sus actividades cotidianas, a causa de su salud física?

	Siempre	Casi siempre	Algunas veces	Sólo alguna vez	Nunca
¿Tuvo que reducir el tiempo dedicado al trabajo o a sus actividades cotidianas?	1	2	3	4	5
¿Hizo menos de lo que hubiera querido hacer?	1	2	3	4	5
¿Tuvo que dejar de hacer algunas tareas en su trabajo o en sus actividades cotidianas?	1	2	3	4	5
¿Tuvo dificultad para hacer hacer su trabajo o actividades cotidianas (por ejemplo, le costó más de lo normal)?	1	2	3	4	5

5. Durante las últimas 4 semanas, ¿con qué frecuencia ha tenido alguno de los siguientes problemas en su trabajo o en sus actividades cotidianas, a causa de algún problema emocional (como estar triste, deprimido o nervioso)?

	Siempre	Casi siempre	Algunas veces	Sólo alguna vez	Nunca
¿Tuvo que reducir el tiempo dedicado al trabajo o a sus actividades cotidianas por algún problema emocional?	1	2	3	4	5
¿Hizo menos de lo que hubiera querido hacer por algún problema emocional?	1	2	3	4	5
¿Hizo su trabajo o actividades cotidianas menos cuidadosamente que de costumbre, por algún problema emocional?	1	2	3	4	5

6. Durante las últimas 4 semanas, ¿hasta qué punto su salud física o los problemas emocionales han dificultado sus actividades sociales habituales con la familia, los amigos, los vecinos u otras personas?

☐ Nada$_1$ ☐ Un poco$_2$ ☐ Regular$_3$ ☐ Bastante$_4$ ☐ Mucho$_5$

7. ¿Tuvo dolor en alguna parte del cuerpo durante las últimas 4 semanas?

☐ No, ninguno$_1$ ☐ Sí,muy poco$_2$ ☐ Sí, un poco$_3$ ☐ Sí,moderado$_4$ ☐ Sí, mucho$_5$ ☐ Sí,muchísimo$_6$

8. Durante las 4 últimas semanas, ¿hasta qué punto el dolor le ha dificultado su trabajo habitual (incluido el trabajo fuera de casa y las tareas domésticas)?

☐ Nada$_1$ ☐ Un poco$_2$ ☐ Regular$_3$ ☐ Bastante$_4$ ☐ Mucho$_5$

9. Las preguntas que siguen se refieren a cómo se ha sentido y cómo le han ido las cosas durante las 4 últimas semanas. En cada pregunta responda lo que se parezca más a cómo se ha sentido usted. Durante las últimas 4 semanas ¿con qué frecuencia...

	Siempre	Casi siempre	Algunas veces	Sólo alguna vez	Nunca
se sintió lleno de vitalidad?	1	2	3	4	5
estuvo muy nervioso?	1	2	3	4	5
se sintió tan bajo de moral que nada podía animarle?	1	2	3	4	5
se sintió calmado y tranquilo?	1	2	3	4	5
tuvo mucha energía?	1	2	3	4	5
se sintió desanimado y deprimido?	1	2	3	4	5
se sintió agotado?	1	2	3	4	5
se sintió feliz?	1	2	3	4	5
se sintió cansado?	1	2	3	4	5

10. Durante las 4 últimas semanas, ¿con qué frecuencia la salud física o los problemas emocionales le han dificultado sus actividades sociales (como visitar a los amigos o familiares)?

☐ Siempre ☐ Casi siempre ☐ Algunas veces ☐ Sólo alguna vez ☐ Nunca

11. Por favor diga si le parece CIERTA o FALSA cada una de las siguientes frases

	Totalmente cierta	Bastante cierta	No lo sé	Bastante falsa	Totalmente falsa
Creo que me pongo enfermo más facilmente que otras personas	1	2	3	4	5
Estoy tan sano como cualquiera	1	2	3	4	5
Creo que mi salud va a empeorar	1	2	3	4	5
Mi salud es excelente	1	2	3	4	5

Anexo 9. *Asthma Quality of Life Questionnaire*

Le rogamos responda a todas las preguntas señalando con un círculo la respuesta que mejor describa cómo se ha encontrado durante las dos últimas semanas debido al asma.

En general, ¿con qué frecuencia durante las 2 últimas semanas?:

	Siempre	Casi siempre	Gran parte del	Parte del tiempo	Poco tiempo	Nunca
1…notó que le faltaba el aire debido al asma?						
2...sintió que le molestaba el polvo, o tuvo que evitar un lugar debido al polvo?						
3...se sintió frustrado o irritado debido al asma?						
4…sintió molestias debido a la tos?						
5…tuvo miedo de no tener a mano su medicación para el asma?						
6…notó una sensación de ahogo u opresión en el pecho?						
7…sintió que le molestaba el humo del tabaco, o tuvo que evitar un lugar debido al humo del tabaco?						
8…tuvo dificultades para dormir bien por la noche debido al asma?						
9…tuvo síntomas de asma por haber estado expuesto al humo de tabaco?						
10…sintió silbidos o pitos en el pecho?						
11…sintió que le molestaba o tuvo que evitar salir de casa debido al tiempo o a la contaminación atmosférica?						
12…esfuerzos intensos (como darse prisa, hacer ejercicio, subir escaleras corriendo, hacer deporte)						
13…esfuerzos moderados (como caminar, hacer las tareas del hogar, trabajar en el jardín o en el huerto, hacer la compra, subir escaleras sin correr)						

¿hasta que punto el asma le ha limitado para hacer estas actividades durante las últimas 2 semanas?

*Lim: limitado	Totalmente lim	Extremadamente lim	Muy lim	Moderadamente lim	Algo lim	Pocol lim	Nada lim
14…actividades sociales (como hablar, jugar con niños/animales domésticos, visitar a amigos/familiares)							
15…actividades relacionadas con su trabajo (tareas que tiene que hacer en su trabajo*)							

* si no está trabajando, responda a esta pregunta pensando en las tareas que tiene que hacer la mayoría de los días.

Clave de las dimensiones:

- Síntomas: 1, 4, 6, 8, 10
- Limitación de actividades: 12, 13, 14, 15
- Función emocional: 3, 5, 9
- Estímulos ambientales: 2, 7, 11

Anexo 10. *Asthma Control Test*

Este cuestionario consta de 5 preguntas, cuyas respuestas se puntuan de 0 a 6. Dichas puntuaciones se suman y el resultado se divide entre 5. Si el resultado es:

- ≤0.75: control adecuado del asma.
- 0.75-1.5 Asma parcialmente controlada.
- >1.50: control inadecuado del asma.

1. En las últimas 4 semanas, ¿durante cuánto tiempo le ha impedido el asma completar sus actividades habituales en el trabajo, la escuela o el hogar?
   - ◻ Siempre (1 punto)
   - ◻ Casi siempre (2 puntos)
   - ◻ A veces (3 puntos)
   - ◻ Pocas veces (4 puntos)
   - ◻ Nunca (5 puntos)

2. Durante las últimas 4 semanas, ¿con que frecuencia ha notado que le faltaba el aire?
   - ◻ Más de una vez al día (1 punto)
   - ◻ Una vez al día (2 puntos)
   - ◻ De 3 a 6 veces por semana (3 puntos)
   - ◻ Una o 2 veces por semana (4 punto)
   - ◻ Nunca (5 puntos)

3. Durante las últimas 4 semanas ¿con qué frecuencia le han despertado por la noche o más temprano de lo habitual por la maána sus síntomas de asma (sibilancias/ìtidos, tos, falta de aire, opresión en el pecho o dolor)?
   - ◻ 4 o más noches por semana (1 punto)
   - ◻ De 2 a 3 noches por semana (2 puntos)
   - ◻ Una vez por semana (3 puntos)
   - ◻ Una o dos veces por semana (4 puntos)
   - ◻ Nunca (5 puntos)

4. Durante las últimas 4 semanas, ¿con qué frecuencia ha utilizado su inhalador o nebulizador de medicación de rescate (por ej. Salbutamol)?
   - ◻ 3 o más veces al día (1 punto)
   - ◻ 1 o 2 veces al día (2 puntos)
   - ◻ 2 o 3 veces por semana (3 puntos)
   - ◻ Una vez por semana o menos (4 puntos)
   - ◻ Nunca (5 puntos)

5. ¿En qué medida diría que su asma ha estado controlada durante las últimas 4 semanas?
   - □ Nada contorlada (1 punto)
   - □ Mal controlada (2 puntos)
   - □ Algo controlada (3 puntos)
   - □ Bien controlada (4 puntos)
   - □ Totalmente controlada (5 puntos)

Anexo 11. Test de Adhesión a los Inhaladores (TAI)

						Puntuación
**1.**	**En los últimos 7 días ¿cuántas veces olvidó tomar sus inhaladores habituales?**					
	☐1. Todas	☐2. Más de la mitad	☐3. Aprox. la mitad	☐4. Menos de la mitad	☐5. Ninguna	
**2.**	**Se olvida de tomar los inhaladores:**					
	☐1.Siempre	☐2. Casi siempre	☐3. A veces	☐4. Casi nunca	☐5. Nunca	
**3.**	**Cuando se encuentra bien de su enfermedad, deja de tomar sus inhaladores:**					
	☐1.Siempre	☐2. Casi siempre	☐3. A veces	☐4. Casi nunca	☐5. Nunca	
**4.**	**Cuando está de vacaciones o de fin de semana, deja de tomar sus inhaladores:**					
	☐1.Siempre	☐2. Casi siempre	☐3. A veces	☐4. Casi nunca	☐5. Nunca	
**5.**	**Cuando está nervioso/a o triste, deja de tomar sus inhaladores:**					
	☐1.Siempre	☐2. Casi siempre	☐3. A veces	☐4. Casi nunca	☐5. Nunca	
**6.**	**Deja de tomar sus inhaladores por miedo a posibles efectos secundarios:**					
	☐1.Siempre	☐2. Casi siempre	☐3. A veces	☐4. Casi nunca	☐5. Nunca	
**7.**	**Deja de tomar sus inhaladores por considerar que son de poca ayuda para tratar su enfermedad:**					
	☐1.Siempre	☐2. Casi siempre	☐3. A veces	☐4. Casi nunca	☐5. Nunca	
**8.**	**Toma menos inhalaciones de las que su médico le prescribió:**					
	☐1.Siempre	☐2. Casi siempre	☐3. A veces	☐4. Casi nunca	☐5. Nunca	
**9.**	**Deja de tomar sus inhaladores porque considera que interfieren con su vida cotidiana o laboral:**					
	☐1.Siempre	☐2. Casi siempre	☐3. A veces	☐4. Casi nunca	☐5. Nunca	
**10.**	**Deja de tomar sus inhaladores porque tiene dificultad para pagarlos:**					
	☐1.Siempre	☐2. Casi siempre	☐3. A veces	☐4. Casi nunca	☐5. Nunca	

Las dos siguientes preguntas las deberá responder el profesional sanitario responsable del paciente según los datos que figuran en su historial clínico (pregunta 11) y tras comprobar su técnica de inhalación (pregunta 12)

**11.**	**¿Conoce o recuerda el paciente la pauta (dosis y frecuencia) que se le prescribió?**		
	☐1. No	☐2. Si	
**12.**	**La técnica de inhalación del dispositivo del paciente es:**		
	☐1. Con errores críticos	☐2. Sin errores críticos o correcta	

PUNTUACIÓN TOTAL

Anexo 12. Escala modificada de la *Medical Research Council*

GRADO	DISNEA
0	Disnea sólo ante actividad física muy intensa.
1	Disnea al andar muy rápido o al subir una cuesta poco pronunciada.
2	Incapacidad de andar al mismo paso que otras personas de la misma edad.
3	Disnea que obliga a parar antes de los 100 metros a pesar de caminar a su paso y en terrenos llanos.
4	Disnea al realizar mínimos esfuerzo de la actividad física diaria como vestirse o que impiden al paciente salir de su domicilio.

Anexo 13. Escala de Borg modificada

10	Máximo
9	Muy, muy fuerte
8	- - - - - - - - - - - - - -
7	Muy fuerte
6	- - - - - - - - - - - - - -
5	Fuerte
4	Un poco fuerte
3	Regular
2	Poco
1	Muy poco
0	Nada

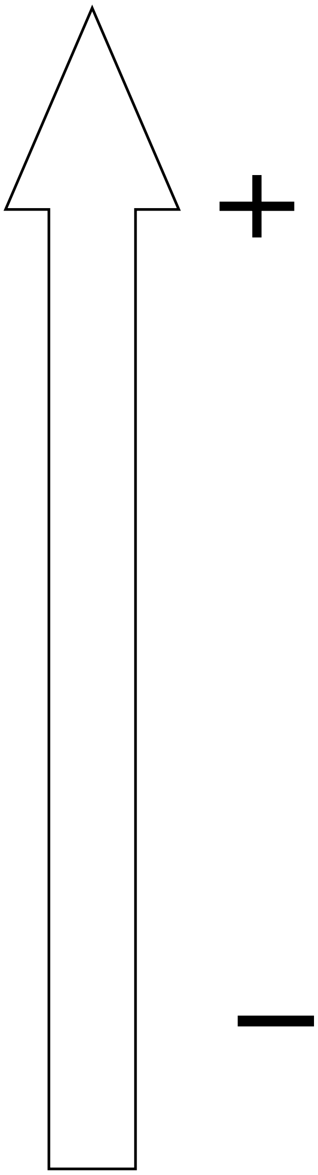

Anexo 14. Plan educacional para los pacientes

1. Presentación del programa y de los pacientes

Las personas al cargo del estudio explicarán a los pacientes como se llevará a cabo el mismo, en materia de intervención, fechas y horarios. Además, los pacientes podrán conocerse entre ellos, ya que una parte del total, el grupo de estudio, realizarán las sesiones juntos.

Es importante que los participantes sean conscientes de lo que supone su patología y de la importancia de un tratamiento continuo a pesar de que no tengan molestias. Es igual de importante que sepan reconocer los síntomas de la enfermedad y los signos y síntomas de agravamiento de la misma, para poder reaccionar ante ellos y prevenir una crisis o exacerbación y que realicen un correcto uso de los inhaladores, monitorización de los síntomas y del flujo espiratorio máximo (PEF) (1).

2. Información sobre el asma

2.1 ¿Qué es el asma? Definición y descripción

Asma: Enfermedad heterogénea caracterizada normalmente por inflamación de la vía aérea de tipo crónico (2), lo que produce estrechez de los bronquios. Además, se acompaña de hiperreactividad o irritabilidad de estos. Los bronquios son los tubos que llevan el oxígeno desde el aire a nuestros pulmones. Esta situación de inflamación e hiperreactividad produce que estos se estrechen o tapen de forma brusca en diferentes situaciones. Si la inflamación se perpetua en el tiempo, se cronifica la enfermedad (3).

Históricamente se ha definido con los siguientes síntomas respiratorios: sibilancias, falta de aire, opresión torácica, tos que varía en el tiempo e intensidad, junto con limitación del flujo aéreo espiratorio variable.

Todos estos síntomas varían en el tiempo y en la intensidad, muchas veces, dependiendo de factores como: ejercicio, exposición a alérgenos o irritantes, cambios en el tiempo o infecciones respiratorias virales.

Los síntomas y la limitación del flujo aéreo se pueden resolver de forma espontánea o mediante tratamiento farmacológico, sin embargo, en otras ocasiones los pacientes pueden sufrir exacerbaciones episódicas que les obliguen a seguir un tratamiento de por vida (2).

2.2 Prevalencia del asma

Afecta a 300 millones de personas en el mundo (4). En adultos esta prevalencia oscila entre el 3 y el 9%, a nivel mundial, y en España ente el 3-4%, siendo en la población del 8%. Es más frecuente en países desarrollados y en los últimos años se ha incrementado su frecuencia, existiendo teóricas que achacan este incremento a la insuficiente exposición a bacterias e infecciones de los niños de estos países, lo que produce un trastorno de la maduración del sistema inmunológico. Es más frecuente en mujeres que en hombres, pero menos frecuente en niñas que en niños.

La frecuencia de la enfermedad provoca que su coste sociosanitario para la sociedad sea muy elevado (3).

2.3 Fenotipos del asma: "tipos de asmáticos"

- Asma alérgica. El más común. Comienza en la infancia. Se asocia historia familiar alérgica como: eccema, rinitis alérgica, alergia alimentaria o a medicamentos. El esputo pretratamiento de estos pacientes muestra muchas veces inflamación de la vía aérea. Normalmente responden vía a los corticoesteroides inhalados.
- Asma no alérgica. No se asocia a ninguna alergia y en el esputo puede haber o no, evidencia de inflamación de la vía aérea. Responden peor a los corticoesteroides inhalados.
- Asma de aparición tardía. Normalmente en mujeres, que sufren el primer episodio en la edad adulta. No se asocia a alergias y necesitan dosis elevadas de corticoesteroides inhalados o son relativamente resistentes a los mismos.
- Asma con limitación del flujo aéreo permanente. Pacientes con asma de larga duración desarrollan una limitación permanente, consecuencia de la remodelación de la vía aérea.
- Asma y obesidad. Pacientes obesos que tienen sintomatología respiratoria importante y pequeña inflamación de la vía aérea (2).

2.4 Exacerbación asmática

Se produce por el estrechamiento de la vía aérea que produce obstrucción al flujo aéreo, que es reversible, principal característica diferenciadora del asma. Este estrechamiento se produce por la contracción del músculo bronquial, edema e hipersecreción mucosa.

Se puede producir por causas directas, como infección viral respiratoria, tabaco, frío, humedad, alérgenos y contaminantes atmosféricos; por causas indirectas, como ejercicio

físico, alérgenos y adictivos alimentarios, embarazo, tormentas o inversión térmica, fármaco, sinusitis, menstruación o reflujo gastroesofágico.

La variabilidad de los síntomas se puede valorar con la medición diaria del PEF.

Son exacerbaciones los episodios agudos o subagudos caracterizados por un aumento progresivo de la disnea, tos, sibilancias o opresión torácica (es decir, alguno de los síntomas típicos), que se acompaña de una disminución del flujo espiratorio (PEF).

Las exacerbaciones pueden ser de instauración lenta, que tardan días o semanas, y son las más frecuentes (80% de las que acuden a urgencias) y las de instauración rápida, que tardan menos de tres horas y tienen causas, patogenia y pronóstico diferentes.

Inicialmente se realiza una evaluación estática, que permite identificar a los pacientes con factores de riesgo, identificar signos y síntomas y medir de forma objetiva el grado de obstrucción. Posteriormente al tratamiento, se realiza una segunda evaluación, que valora los cambios obtenidos y la necesidad de realizar más pruebas diagnósticas.

La exacerbación se clasifica como leve, moderada o grave en función del valor obtenido con el PEF o FEV_1.

	PEF O FEV_1
Leve	>70%
Moderada	50-70%
Grave	<50%

PEF: Pico espiratorio forzado. FEV_1: fracción de aire expulsada al primer segundo.

El tratamiento consiste en revertir la obstrucción al flujo aéreo y la hipoxemia, de estar presente. Lo principal es preservar la vida del paciente, por lo que, superado este problema, será cuando se pase a revisar el plan terapéutico para prevenir nuevas crisis.

Las crisis catalogadas como leves pueden ser tratas por el paciente de forma independiente o bien en un servicio de urgencias hospitalario. El tratamiento consiste en la aplicación de broncodilatadores agonistas B2 adrenérgicos de acción rápida, glucocorticoides orales y oxígeno, de ser necesario. En cuanto a los broncodilatadores agonistas B2 adrenérgicos se emplea salbutamol o terbutalina, a dosis que oscilan entre los 200 y 400ug, con cámara de inhalación. Se realizarán de 2 a 4 inhalaciones cada 20 minutos durante la primera hora. Si la situación no mejora, el paciente deberá ser derivado a un servicio de urgencias hospitalario,

en caso contrario, se continua la inhalación de salbutamol a dosis de una a dos inhalaciones cada 3-4 horas, hasta que la crisis remita totalmente.

La evolución se considera favorable si desaparecen los síntomas y el PEF es superior al 80% del valor de referencia o del mejor valor del paciente.

En caso de que no se consiga remisión de la obstrucción con este tratamiento, el paciente los haya estado tomando ya, haya tratado ya su crisis con otras opciones terapéuticas y haya fracasado o en caso de que existan antecedentes de exacerbaciones previas que lo requirieran, el paciente debe tomar glucocorticoides orales.

En caso de exacerbaciones moderadas y graves, el paciente debe acudir siempre a un servicio médico (1).

2.5 Control del asma

El control del asma tiene dos partes: control de los síntomas (tiempo sin síntomas, reducción o eliminación de los mismos con tratamiento farmacológico) y riesgo de exacerbaciones. Es importante destacar que el control del asma no se trata de lo rápido que se resuelven los síntomas cuando se toman la medicación (2).

2.5.1 Asma de difícil control

Afecta a un 5% de los pacientes asmáticos. El asma de difícil control hace referencia a aquellos pacientes cuya enfermedad asmática se caracteriza por ser muy agresiva y por estar mal controlada o de forma insuficiente (1).

3 Medicación

3.1 Tipos de medicamentos

Medicamentos de control.

Son usados para reducir la inflamación de la vía aérea, controlar los síntomas y reducir riesgo futuro de exacerbaciones y disminución de la función pulmonar.

Medicamentos de rescate

Son recomendados para prevenir a corto plazo la broncoconstricción inducida por el ejercicio. Un objetivo debe ser reducir o incluso eliminar la necesidad de recurrir a este tipo de medicamentos. Este logro es una medida de éxito en el tratamiento del asma (4).

3.2 Correcta administración de los inhaladores

Existen diferentes tipos de inhaladores, en función de la forma de presentación de la dosis: inhalador presurizado, inhalador presurizado con solución de partículas extrafinas, dispositivos de polvo, y nebulizadores.

Inhalador presurizado	Con cámara espaciadora
	Sin cámara espaciadora
Inhalador presurizado con solución de partículas extrafinas	
Dispositivos de polvo seco	
Nebulizadores	

Además, los inhaladores presurizados pueden variar su forma de aplicación, si se añade cámara espaciadora o no.

Se decide por uno u otro en función de la edad y de la destreza del paciente en el uso de cada uno de ellos. Los cartuchos presurizados requieren coordinación entre la inhalación del mismo y el movimiento del pulmón, y los cartuchos de polvo dependen del flujo, que debe ser alto (más de 60 l/minutos), ya que de él depende el depósito de partículas.

Para solventar el problema de falta de coordinación entre inhalación y movimiento del pulmón, durante el uso de los cartuchos presurizados, se puede utilizar una cámara espaciadora. Esta además mejora la distancia y la cantidad de medicamento que llega al área bronquial. Disminuye el depósito en la región orofaríngea, la tos, la candidiasis y el riesgo de efectos deletéreos sistémicos.

Hay que elegir el que mejor se adapte, explicar la pauta de inhalación, entrenar la misma, corregirla en caso de ser necesario y revisarla en cada visita.

Los nebulizadores se usan solo en situaciones especiales (4).

3.2.1 Inhalador de cartucho presurizado

Mantenimiento de los dispositivos:

- No exponer a temperaturas superiores a 50ºC.
- Conservarlos protegidos de la luz solar directa.
- Conservación a temperaturas menores de 30ºC.

- Alejar del fuego. Aunque este vacío.
- Realizar 2-4 pulsaciones al aire al usarlo la primera vez, al igual que si han pasado varios días desde el último uso.

Limpieza de los dispositivos:

- Se extrae el cartucho y se lava la carcasa con agua y jabón neutro.
- Aclarar con abundante agua y secar bien (insistir en zona donde se asienta la válvula).
- Volver a insertar el cartucho.
- En sistemas compactos, en los que el cartucho no se extrae, es preferible limpiar el envase con un pañuelo húmedo o seco, pero no se aconseja desmontarlos ni mojarlos. (5)

Uso correcto del inhalador de cartucho presurizado:

1. Para pMDI agitar antes de cada dosis, para asegurar la homogeneidad de la dosis.
2. Retirar la tapa del pMDI.
3. Pulsación al aire si es la primera vez que se usa o han pasado 1-2 semanas desde su último uso.
4. Colocar el dispositivo entre los dientes y sellar los labios alrededor de la pieza bucal.
5. Exhalar hasta cerca del volumen residual.
6. Activar el cartucho una sola vez. Comenzar a espirar después (no más de 3 segundos), lentamente hasta la capacidad pulmonar total. Inspiración lenta y profunda: menos de 30litros/minuto.
7. Apnea de 10 segundos posterior a la inhalación total. Aumenta la permanencia de las partículas en los pulmones, por lo que favorece el depósito por sedimentación y difusión.
8. Si se necesitan dos dosis se repite el proceso. Realizar dos pulsaciones al mismo tiempo reduce la cantidad de fármaco que alcanza los pulmones. (6)

Mantenimiento y limpieza de la cámara

Son cámaras personales, de uso individual. Requiere una limpieza de al menos una vez por semana. Se debe desmontar por completo y lavar con agua templada y detergente suave. Se deja secar sin frotar, para de esta forma evitar la carga electroestática que podría atraer las partículas de aerosol a las paredes de la cámara, lo que disminuiría la cantidad de aerosol que llega a los pulmones.

Es necesario revisar las válvulas antes de utilizar el inhalador, y si no están en buen estado, o si tienen fisuras, se debe reemplazar la cámara.

3.2.2 Inhaladores de polvo seco

(Dry power inhaler-DPI)

Mantenimiento

- Guardar el inhalador en lugar seco, preservado de la humedad.

Limpieza

- Limpiar el dispositivo con un paño sin pelusa o con un papel seco, alrededor de la boquilla después de uso.
- Cerrar posteriormente el inhalador y guardarlo (5).

Uso correcto del inhalador de polvo seco

1. Quitar la tapa de la pieza bucal.
2. Abrir el DPI, insertar la capsula inmediatamente, y cerrarlo.
3. Presionar el botón situado en la base del DPI para perforar la cápsula.
4. Vaciar los pulmones evitando exhalar dentro de la pieza bucal después de perforar la cápsula.
5. Colocar la pieza bucal en la boca. Labios cerrados alrededor y evitar que la lengua obstruya.
6. Inhalar desde el principio con el máximo esfuerzo inspiratorio, tan fuerte y profundo como pueda, aguantándolo el mayor tiempo posible.
7. Dejar de inhalar solo después de haber llenado los pulmones totalmente.
8. Retirar el DPI de la boca sin exhalar dentro.
9. Aguantar la respiración de 8-10 segundos cuando se complete la inhalación. (6)

3.2.3 Nebulizadores

Existen varios tipos de nebulizadores: neumáticos o jet, ultrasónicos y de malla vibrante. Se diferencian entre sí por la forma de conseguir el aerosol: atomización, cristal piezoeléctrico que convierte la energía eléctrica en ondas ultrasónicas que genera el aerosol al atravesar la solución o energía eléctrica, respectivamente.

- Se pueden utilizar mediante piezas bucales o mascarillas.
  - Evitar la respiración nasal.
  - Evitar la fuga del aerosol.
  - Evitar la impactación del aerosol en la cara.
- Se recomienda un patrón respiratorio lento, a volumen corriente, intercalando inspiraciones profundas y evitando la hiperventilación.

- El uso de reservorio, de válvulas que aumenten la superficie de salida durante la fase inspiratoria, o el hecho de que la nebulización se proporcione solo durante la fase inspiratoria, disminuye la pérdida del aerosol, dependiendo todo esto del diseño de este. (5)

4. ¿Qué puedo hacer yo?

4.1 Control respiratorio

Lo que se debe modificar, principalmente, es:

- Número de respiraciónes por minuto.
- Profundidad de la respiración (volumen de aire inspirado de cada vez).
- Velocidad del flujo.
- Timing (inspiración/espiración)
- Ritmo.
- Región principal en la que se produce el movimiento.

No existe evidencia de que los movimientos del pecho afecten a la distribución regional de la respiración, pero se ha propuesto que los aferentes anormales propioceptivos que se asocian con una respiración costal superior, pueden directamente, aumentar la percepción de los síntomas respiratorios (7).

Se propone inicialmente controlar el ritmo de la respiración, antes que el volumen de la misma. Para ello se sugiere alargar los tiempos espiratorios y las pausas al fial de la espiración.

En pacientes con asma, la respiración nasal se ha visto asociada con la disminución de los síntomas nocturnos (8). Además, la respiración bucal, aparece asociada a momentos de crisis (9). Además, es siempre preferible respirar por la nariz, ya que es un filtro, calentador y humidificador del aire inspirado, lo que potencialmente puede reducir el impacto del asma (10).

En cuanto a las pausas, se han asociado con multiples beneficios teoricos. Por ejemplo, las pausas de 3 segundos al final de la inspiracion (pausas teleinspiratorias) se asocian con una mejor distribución de la ventilación, y permite al aire superar la barrera de las secreciones (11). Tambien desensibiliza a los sujetos con elevadas concentraciones de dioxido de carbono y reduce el ratio respiratorio (12). Hay diferentes formas de realizar pausas: inspiratorias/espiratorias, diferentes volumenes (inspiracion maxima o VR), aguantar con nariz o no, en reposo o durante la actividad. Lo más usado: pausas al final de la espiración, a traves

de la nariz, hasta la sensación de disconfort, pero no tanto como para tener que realizar una respiración profunda posteriormente.

Por tanto

Respiraciónes nasales, 1:3 inspiracion:espiración, pausa al final de la espiración hasta la incomodidad pero no para que necesite una inspiración profunda posterior, y de forma diafragmatica, no usando la parte costal superior

4.2 Estilo de vida

Recomendaciones generales:

- Tener todas las vacunas obligatorias al día.
- Vacunarse de la gripe. La gripe puede contribuir a las exacerbaciones del asma, aunque hay que saber que vacunarse no va a reducir las mismas. Solo evitar que aparezcan en mayor medida.
- Dejar de fumar y evitar los ambientes con humo de tabaco (1,2).
- Realizar actividad física. Importante para la salud general. Mejora la salud cardiopulmonar (2). En cuanto a la broncoconstricción inducida por el ejercicio:
  - Se recomienda inhalación de un beta-agonista de corta acción (SABA) 15 minutos antes de la realización del ejercicio (13).
- Dieta saludable, rica en fruta y vegetales.
- Reducir el peso en caso de sobrepeso u obesidad.

4.3 Control del ambiente

- Evitar el uso de fuentes caloríficas y de cocina contaminantes, en caso de ser inevitable, al aire libre en la medida de lo posible.
- Evitar la contaminación ambiental.
- Afrontar el estrés emocional.
- Evitar realizar ejercicio y permanecer largos periodos de tiempo al aire libre, bajo condiciones climáticas desfavorecedoras: temperaturas muy frias, baja humedad o contaminación aérea importante (2).
- En caso de asma alérgica, se recomienda también disminuir la exposición al alérgeno.

Todas las acciones que podamos realizar suman para conseguir el control ambiental y con ello las crisis, las exacerbaciones y los síntomas asmáticos.

En caso de necesitar un analgésico se recomienda el paracetamol, en dosis menores de 650mg/toma. Los antiinflamatorios no esteroides no están recomendados, ya que pueden generar broncoconstricción (1)

5. Material

Se proporcionará a cada paciente una guía con la información arriba explicada. Anexo 16.

Se proporcionará en esta reunión también el diario en el que tienen que anotar todo lo relativo a su actividad física diaria, tos, expectoración, disnea, toma de medicamentos, visitas a urgencias y adherencia (Anexo 2).

1. Sociedad Española de Neumología y Cirugía Torácica. GEMA 2009: Guía Española para el manejo del asma. Madrid: Luzán 5; 2009.

2. Global Initiative for Asthma. Global Strategy for Asthma Management and Prevention, 2018 [Internet]. [citado 28 de diciembre de 2018]. Disponible en: www.ginasthma.org

3. Calvo Corbella E, Grupo de Respiratorio de Atención Primaria, Sociedad Española de Medicina Rural y Generalista, Sociedad Española de Neumología y Cirugía Torácica, Sociedad Española de Neumología Pediátrica, Associació Asmatològica Catalana. Guía Española para el Manejo del Asma (GEMA) para pacientes, padres y amigos. Barcelona; 2005.

4. Global Initiative for Asthma. Global Strategy for Asthma Management and Prevention. Online Appendix, 2018. [Internet]. Disponible en: www.ginasthma.com

5. Consenso SEPAR-ALAT sobre terapia inhalada. Archivos de Bronconeumología. 2013;49:2-14.

6. Sociedad Española de Neumología y Cirugía Torácica (SEPAR). Terapia inhalada. Teoría y práctica. [Internet]. [citado 28 de diciembre de 2018]. Disponible en: https://issuu.com/separ/docs/terapia_inhalada._teoria_y_practica

7. Howell JB. The hyperventilation syndrome: a syndrome under threat? Thorax. 1997;52. Suppl. 3:S30-4.

8. Petruson B, Theman K. Reduced nocturnal asthma by improved nasal breathing. Acta Otolaryngol. 116.ª ed. 1996;490-2.

9. Kairaitis K, Garlick SR, Wheatley JR, et al. Route of breathing in patients with asthma. Chest. 1999;116:1646-52.

10. D. Price Q. Zhang V. S. Kocevar D. D. Yin M. Thomas. Effect of a concomitant diagnosis of allergic rhinitis on asthma-related health care use by adults. Clin Exp Allergy. 2005;35:282-7.

11. McIlwaine M. Physiotherapy and airway clearance techniques and devices. Paediatr Respir Rev. 7. Supl 1. 2006;S220-2.

12. Courtney R, Cohen M. Investigating the claims of Konstantin Buteyko MD PhD: the relationship of breath holding time to end tidal CO2 and other proposed measures of dysfunctional breathing. J Altern Complement Med. 14.ª ed. 2008;115-23.

13. Parsons JP, Hallstrand TS, Mastronarde JG, Kaminsky DA, Rundell KW, Hull JH, et al. An Official American Thoracic Society Clinical Practice Guideline: Exercise-induced Bronchoconstriction. American Journal of Respiratory and Critical Care Medicine. 2013;187(9):1016-27.

Anexo 15. Guion grupos focales.

- ¿Qué significa, en vuestra experiencia, tener asma?
- ¿Qué retos os presenta diariamente? ¿Qué miedos os provoca para afrontar vuestro día a día?
- ¿Qué aprendizajes os supuso y os supone?
- ¿Por qué recomendaríais a otra persona realizar marcha nórdica/participar en este tipo de estudios? ¿Qué aspectos positivos encontráis en la actividad?
- ¿Qué aspectos negativos encuentras en realizar estas intervenciones?
- ¿Qué ha cambiado en tu forma de convivir con el asma después de realizar está intervención?
- ¿Qué te gustaría poder cambiar, de tener la oportunidad de modificar en algo las sesiones/intervenciones?
- ¿Del diseño de las intervenciones, qué aspectos consideras que no se deben modificar?
- ¿Qué otros tratamientos habías probado para el asma a parte de la medicación convencional?

Anexo 16. Guía del paciente

Uso de un inhalador de cartucho presurizado

Mantenimiento	Limpieza
No exponer a temperaturas superiores a 50ºC.<br>Consérvalos protegidos de la luz solar directa.<br>Conservación a temperaturas menores de 30ºC.<br>Alejar del fuego. Aunque este vacío.<br>Realizar 2-4 pulsaciones al aire al usarlo la primera vez, al igual que si han pasado varios días desde el último uso.	Se extrae el cartucho y se lava la carcasa con agua y jabón neutro.<br>Aclarar con abundante agua y secar bien (insistir en zona donde se asienta la válvula).<br>Volver a insertar el cartucho.<br>En sistemas compactos, en los que el cartucho no se extrae, es preferible limpiar el envase con un pañuelo húmedo o seco, pero no se aconseja desmontarlos ni mojarlos.

Uso del inhalador
Para pMDI agitar antes de cada dosis, para asegurar la homogeneidad de la dosis.<br>Retirar la tapa del pMDI.<br>Pulsación al aire si es la primera vez que se usa o han pasado 1-2 semanas desde su último uso.<br>Colocar el dispositivo entre los dientes y sellar los labios alrededor de la pieza bucal.<br>Exhalar hasta cerca del volumen residual.<br>Activar el cartucho una sola vez. Comenzar a inspirar después (no más de 3 segundos), lentamente hasta la capacidad pulmonar total. Inspiración lenta y profunda: menos de 30litros/minuto.<br>Apnea de 10 segundos posterior a la inhalación total. Aumenta la permanencia de las partículas en los pulmones, por lo que favorece el depósito por sedimentación y difusión.<br>Si se necesitan dos dosis se repite el proceso. Realizar dos pulsaciones al mismo tiempo reduce la cantidad de fármaco que alcanza los pulmones.

Puede encontrar en la aplicación ASMACONTROL un video que le ayudará a recordar el protocolo correcto. Vídeo: MDI.

Uso de un inhalador de cartucho presurizado con cámara espaciadora

Cámara espaciadora
Evita la necesidad de coordinación entre la inhalación y el movimiento del pulmón.<br>Mejora la distancia y la cantidad de medicamento que llega al árbol bronquial.<br>Disminuye el depósito en la región orofaríngea, la tos, la candidiasis y el riesgo de efectos deletéreos secundarios.<br>Limpieza y mantenimiento:<br>Se requiere limpiar la cámara al menos una vez por semana. Para ello se debe desmontar por completo, lavar con agua templada y con detergente suave.<br>se deja secar sin frotar, para evitar la carga electrostática que provocaría que las partículas de aerosol se peguen a las paredes de la misma, disminuyendo la cantidad del mismo que llega a los pulmones.<br>Se deben revisar las válvulas antes de usar el inhalador y se reemplazara la cámara en caso de que no estén en buen estado o tengan fisuras.

Puede encontrar en la aplicación ASMACONTROL un video que le ayudará a recordar el protocolo correcto. Vídeo: MDI+CÁMA

Uso de un inhalador de polvo seco

Mantenimiento	Limpieza
Guardar el inhalador en lugar seco, preservado de la humedad.	Limpiar el dispositivo con un paño sin pelusa o con un papel seco, alrededor de la boquilla después de uso.<br>Cerrar posteriormente el inhalador y guardarlo.
Uso correcto del inhalador	
Quitar la tapa de la pieza bucal.<br>Abrir el DPI, insertar la cápsula inmediatamente, y cerrarlo.<br>Presionar el botón situado en la base del DPI para perforar la cápsula.<br>Vaciar los pulmones evitando exhalar dentro de la pieza bucal después de perforar la cápsula.<br>Colocar la pieza bucal en la boca. Labios cerrados alrededor y evitar que la lengua obstruya.<br>Inhalar desde el principio con el máximo esfuerzo inspiratorio, tan fuerte y profundo como pueda, aguantándolo el mayor tiempo posible.<br>Dejar de inhalar solo después de haber llenado los pulmones totalmente.<br>Retirar el DPI de la boca sin exhalar dentro.<br>Aguantar la respiración de 8-10 segundos cuando se complete la inhalación	

Puede ver un vídeo explicativo en ASMACONTROL buscando el nombre del que usa. Si no aparece, puede guiarse por los videos que hay, ya que son similares

Nebulización

Uso correcto de la nebulización
Se recomienda un patrón respiratorio lento<br>Volumen corriente.<br>Se intercalan inspiraciones profundas.<br>Evitar hipervetilación.

Consejos para una vida plenamente saludable

Recomendaciones generales:
Tener todas las vacunas obligatorias al día.<br>Vacunarse de la gripe. La gripe puede contribuir a las exacerbaciones del asma, aunque hay que saber que vacunarse no va a reducir las mismas. Solo evitar que aparezcan en mayor medida.<br>Dejar de fumar y evitar los ambientes con humo de tabaco.<br>Realizar actividad física. Importante para la salud general. Mejora la salud cardiopulmonar. En cuanto a la broncoconstricción inducida por el ejercicio: se recomienda inhalación de un beta-agonista de corta acción (SABA) 15 minutos antes de la realización del ejercicio<br>Dieta saludable, rica en fruta y vegetales.<br>Reducir el peso en caso de sobrepeso u obesidad.<br>Evitar el uso de fuentes caloríficas y de cocina contaminantes, en caso de ser inevitable, al aire libre en la medida de lo posible.<br>Evitar la contaminación ambiental.<br>Afrontar el estrés emocional.<br>Evitar realizar ejercicio y permanecer largos periodos de tiempo al aire libre, bajo condiciones climáticas desfavorecedoras: temperaturas muy frías, baja humedad o contaminación aérea importante.<br>Utilizar fundas antiácaros en el colchón (especialmente casos de asma alérgica).<br>Utilizar acaricidas (especialmente asma alérgica).<br>En caso de asma alérgica, se recomienda también disminuir la exposición al alérgeno.

Si tienes cualquier duda puedes contactar con el equipo investigador escribiendonos un SMS o Whatsapp, llamando o enviandonos un correo a través de los siguientes medios:

Teléfono: +34 628 10 10 24 *Correo electrónico: mn.udc2019@gmail.com*

Anexo 17. Programa de entrenamiento

Calentamiento

<table>
<tr><td colspan="2">Movilidad articular</td><td></td><td></td></tr>
<tr><td>Tobillos:
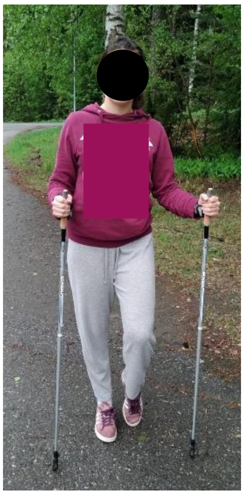</td><td colspan="2">Rodillas:
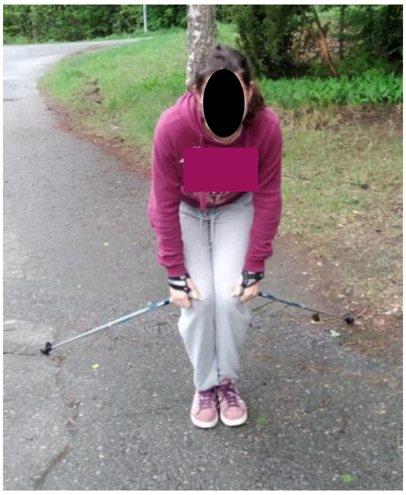</td><td rowspan="2">10 x pierna x sentido</td></tr>
<tr><td>Cadera (circunducción)
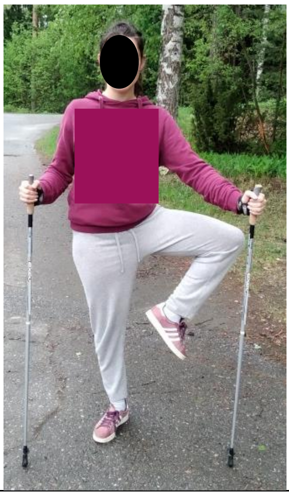</td><td colspan="2">Cadera (flexo.extensión)
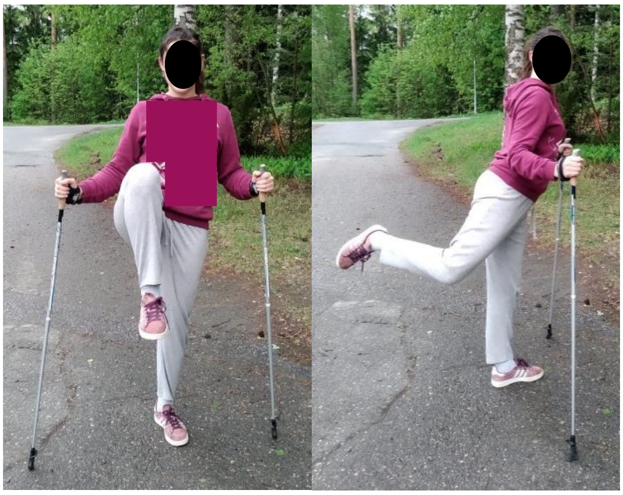</td></tr>
</table>

Movilidad articular de MMSS con los bastones	
Circunducción: 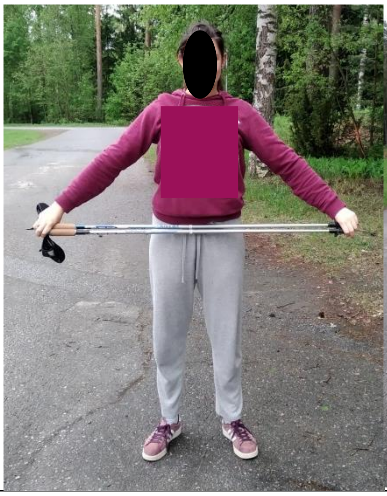 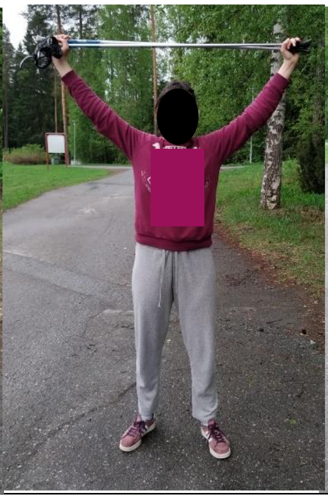 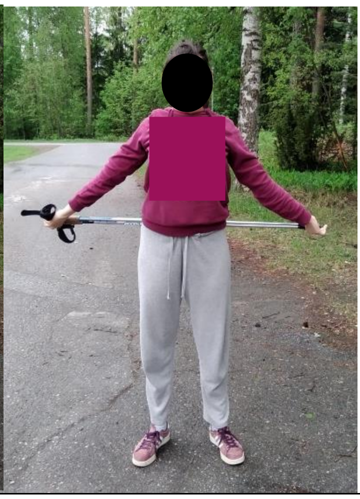	X 10
Push up: 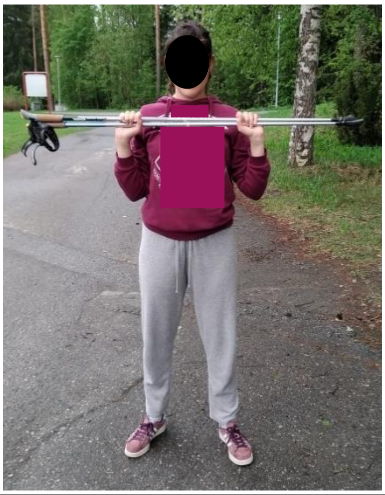 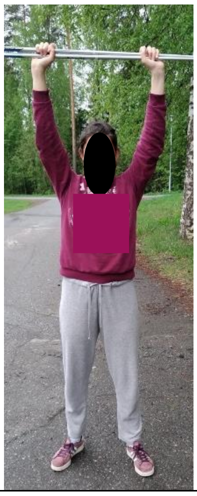	
Remo: 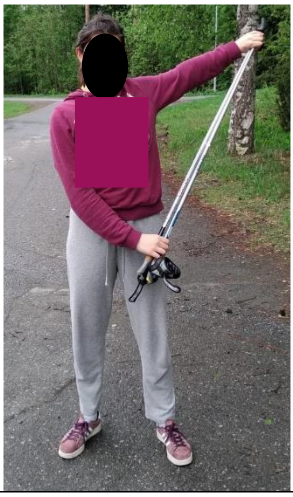	

Bastones en suelo por delante del cuerpo. Pies en posición más ancha que los bastones. Se realiza sentadilla al mismo tiempo que los MMSS realizan una ABD oblicua que termina con ambos brazos paralelos entre si. Es decir, al mismo tiempo que se realiza la sentadilla, los brazos realizan un circulo imaginario que se cierra por delante:<br>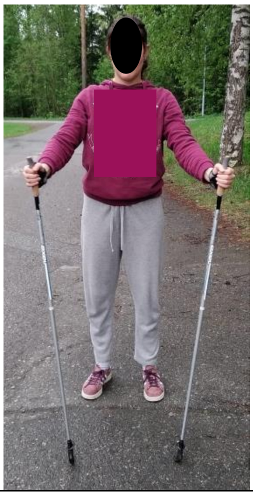 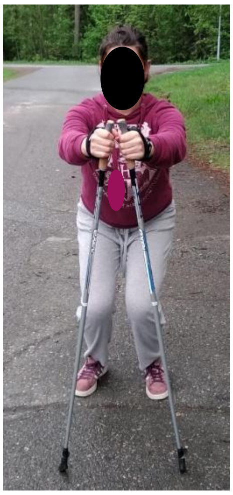	x 10

Bastones en el suelo por delante del cuerpo, más o menos a la misma anchura que los pies. Se realiza “semisentadilla” (recorrido más corto) al mismo tiempo que se realiza un movimiento de rotación externa glenohumeral, por lo que los brazos se abren:<br>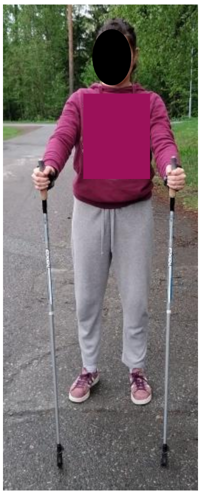 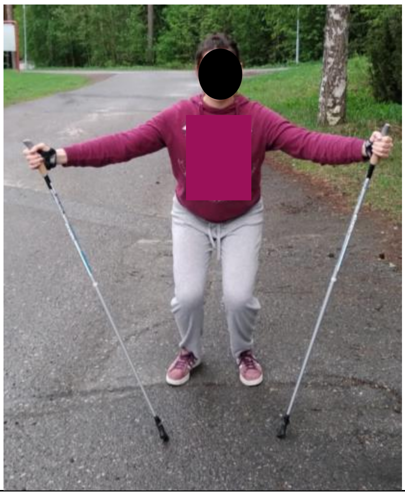	x 10

“Lucha con bastones”. Semi flexion de rodilla y sujetando los bastones por delante del cuerpo, se mueven los brazos de forma alterna, coordinada y rápida realizando movimiento de “dar un puñetazo” 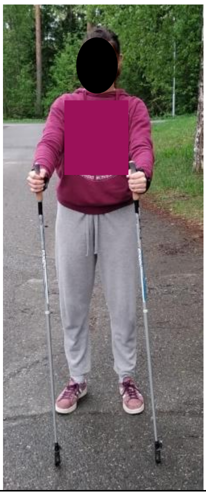	x 10

Con los bastones adelantados, realizamos zancadas en el sitio 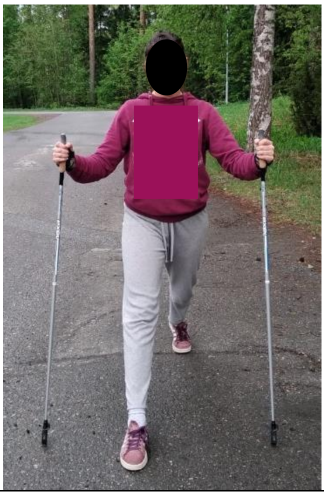 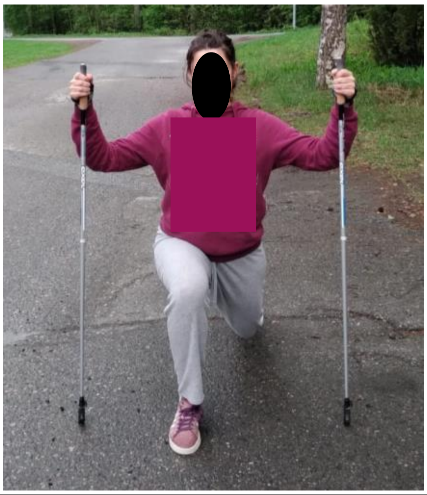	x 10

Saltos en el sitio con los bastones en el suelo	x 10

Marcha a ritmo normal durante 5 minutos y con los bastones	x 10

Parte principal

Los participantes realizarán 30 minutos de marcha nórdica continua.

Incialmente se trabajará al 50% de la FCmáx teórica, y se irá incrementando gradualmente, un 5% por semana, hasta alcanzar la FC máxma de trabajo, que sera un 75% de la FCmáx teórica.

Se indicará a los participantes deberán trabajar entre un 4 y 6 de disnea y fatiga de MMII en la Escala modificada de Borg.

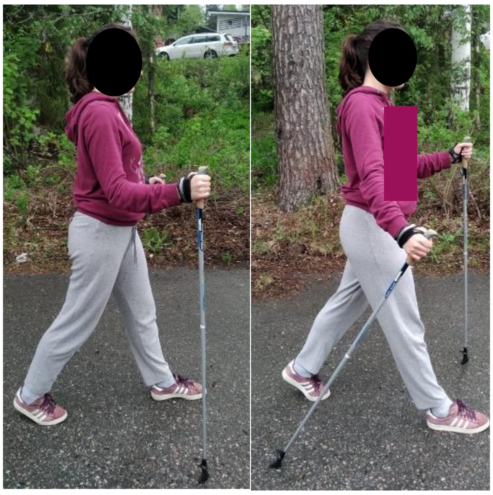

Vuelta a la calma

Estiramientos

Se realizarán estiramientos de los principales músculos implicados en la marcha. Se estirará tríceps sural, cuádriceps, isquiotibiales, glúteos, piramidal, dorsal ancho, pectoral, tríceps.

Tríceps sural
Cuadríceps
Isquiotibiales
Glúteos
Piramidal
Dorsal ancho
Pectoral
Tríceps

Relajación

Realizaremos 5 minutos de relajación para terminar la sesión. Los pacientes sentados (si hay bancos o muros de adecuada altura, sino sentados en el suelo), serán conscientes de su

respiración durante 5 minutos. Se centrarán en diferentes sensaciones secuencialmente. Desde la sensación de respiración en su nariz, como el aire entra y sale, hasta notar como los pulmones se inflan y desinflan al igual que su barriga. Instruir en que deben desviar su pensamiento hacia esas sensaciones siempre que este se disperse en otros temas o sentimientos.

Cuadro resumen

Calentamiento	Parte principal	Vuelta a la calma
Movilidad articular:<br>• Tobillos.<br>• Rodillas.<br>• Cadera.<br>• Hombros.<br>Sentadilla + circulo<br>Semisentadilla + RE<br>Golpes al aire<br>Zancada<br>Saltos<br>Caminar a ritmo lento	Caminar a la FC pautada	Estiramientos:<br>• Tríceps sural.<br>• Isquiotibiales.<br>• Cuádriceps.<br>• Glúteos.<br>• Piramidal.<br>• Dorsal ancho.<br>• Pectoral.<br>• Tríceps.<br>Relajación
