# Supplementary material for: Effectiveness of nordic walking in patients with asthma: A study protocol of a randomized controlled trial
Source: PLoS One. 2023 Mar 9;18(3):e0281007. doi: 10.1371/journal.pone.0281007 (PMC9997906; doi:10.1371/journal.pone.0281007)
Supplement: S6 Appendix — (PDF) [file pone.0281007.s007.pdf]

COMPARISON OF A NORDIC WALKING PROGRAM VERSUS EDUCATIONAL SESSIONS ALONE IN PATIENTS WITH ASTHMA

Main researcher

María Vilanova Pereira

Collaborator researchers

Dra. Ana Lista Paz

Margarita Barral Fernández

Dr. Alejandro Quintela del Río

Dra. Larissa Patricia Fontán García Boente

Dra. Marina Blanco Aparicio

Dra. Manuel Jorge Rial Prado

Tabla de contenido

Tabla de contenido II

Índice de acrónimos y abreviaturas .... **¡Error! Marcador no definido.**

1.Abstract 6

Introduction 6

Objective 6

Methodology 6

Keywords 6

2. Contextualization 7

2.1 Background 7

3.1.1 Nordic walking 7

2.1.2 Asthma y Physiotherapy 15

2.2 Justificación del trabajo 17

3. Hypotheses and objectives 18

3.1 Hypothesis: null and alternative 18

3.2 Research question 18

3.3 Objectives: general and specific 18

4. Methodology 19

4.1 Scope of study 19

4.2 Study period 19

4.3 Type of study 19

4.4 Selection criteria 20

Inclusion criteria: 20

Criterios de exclusión: 20

4.5 Sample size justification 21

4.6 Sample selection 21

4.7 Description of the variables 21

4.7.1 Exercise tolerance 22

4.7.2 Daily physical activity level 22

4.7.3 Quality of life 22
4.7.4 Asthma control and symptoms 23
4.7.5 Dyspnea 23
4.7.6 Pulmonary function 23
4.7.7 Respiratory muscle strength 23
4.7.8 Muscular strength-endurance 23
4.7.9 Medication taking 23
4.7.10 Emergency visits 23
4.7.11 Adherence 23
4.7.12. Qualitative data 24
4.8 Measurements and intervention 24
4.8.1 Measurements 24
4.8.2 Intervention 29
4.9 Statistic analysis 31
5. Timeline and stages of development 32
5.1. Project design 32
5.2 Ethics Committee. 32
5.3 Sample selection 32
5.4 Investigation Development 33
5.5 Analysis of the results. 33
5.6 Dissemination of results. 33
6. Ethical-legal aspects. 33
7. Applicability of the study 34
8. Results dissemination plan 36
8.1 Congresses 36
8.2 Journals 36
9. Economic memory 37
9.1 Necessary resources 37
9.2 Possible funding sources 37

10. Available means and research team to carry out the proposal. 38

10.1 Research team experience. 38

10.2 Material resources available 39

11. Bibliography 40

12. Appendix 49

Appendix 1: Contraindications for participation in the study. 49

12.2.1 Contraindicaciones para la realización de la prueba de 6MWT. 49

12.2.2 Contraindications for performing a spirometry. 49

12.2.3 Classification of asthma exacerbations 50

Appendix 2. Patient's Diary 51

Appendix 3. Patient Information Sheet 52

Appendix 4. Informed consent 57

Appendix 5. Patient data collection notebook 59

Appendix 6. Record of the 6-minute walking test 65

Appendix 7. International Physical Activity Questionnaire. Short version. 66

Anexo 8. Cuestionario de calidad de vida *Short Form- Health Survey* 36 68

.... 72

Appendix 9. *Asthma Quality of Life Questionnaire* 73

Anexo 10. *Asthma Control Test* 74

Anexo 11. Test de Adhesión a los Inhaladores (TAI) 76

Appendix 12. Modified *Medical Research Council* 77

Anexo 13. Borg modified scale 78

Appendix 14. Focus group script 79

Índice de tablas

Table 1. Length of the Nordic walking pole depending on the height of the person. 5
Table 2. Classification of asthma based on symptoms. 14
Table 3 . Minimum clinically significant distance of the SF-36 classified by sections. 21
Table 4. Study variables and measurement instrument. 23
Table 5. Educational plan for patients 28
Table 6. Training plan. 29
Table 7. Schedule and work plan 31
Table 8. Necessary material. 36

1.Abstract

Introduction

Nordic walking is a type of walk that differs from the traditional one mainly by the use of two sticks specially designed for it, that are used to propolse. It has shown multiple benefits with respect to the traditional walk, as well as benefits in multiple cardiovascular, musculoskeletal and respiratory pathologies. So far, it has not been studied in relation to asthma

Objective

The main objective in this study is to analyse if Nordic walking has, compared with conventional cares and recommendations, benefits in asthmatic patients.

Methodology

To answer the research question, a simple blind randomized clinical trial, with patients recruited by the doctors of pulmonology and allergology of the A Coruña University Hospitalary Complex (CHUAC) and pulmonology of the Hospital HM Modelo of A Coruña. This program will be combined with an educational plan in the study group, while the control group will only participate in the recommendations and advice on conventional care. The program will last 8 weeks and will consist of 3 weekly sessions in which the participants will perform 30 minutes of Nordic walking at an incremental intensity throughout the sessions, starting at 50% and increasing 5% per week, until reaching 75% of the theoretical maximum heart rate. Possible differences in exercise tolerance, physical activity level, quality of life, asthma control and symptoms, dyspnoea, pulmonary function, strength-endurance of the musculature, wheezing, taking medications, visits to the emergency system and adherence to treatment will be analysed. Qualitative data will be achieved through focus groups meetings, with semi-structured interviews guides, and qualitative data will be analysed through thematic qualitative analysis.

Keywords

Asthma; Exercise; Walking; Physical Therapy, Modalities; Exercise Tolerance

2. Contextualization

The objective of this epigraph is to explain to reader what is and how Nordic walking (NW) appears, talking about its multiple physiological and biomechanical benefits, and its benefits in different diseases and conditions, focusing specially in the cardiovascular an respiratory ones, being to which this project are oriented.

Asthma disease are treated as well, being the pathology selected to do this investigation, seen until the redaction of this manuscript, any scientific investigation has been developed mixing both: NW and asthma. Furthermore, last available evidence about asthma management from physiotherapy are explained before present the investigation project.

2.1 Background

3.1.1 Nordic walking

NW is a kind of exercise that use two special designed sticks that impulse the traditional walking, respecting biomechanics and natural posture of this traditional way of walk (1). Until this moment, it has been studied related with its multiple benefits in pathologies as rheumatic, neurological, as Parkinson, cardiovascular or respiratory diseases (2-4)

2.1.1.1 History

Is not clear what the origin of NW is. World Record – Nordic Walking dates the origin in 30s, born as summer training to cross-country skiers, seeing the activity can be done in dirt and asphalt roads (5).

However, the International Nordic Walking Association dates the birth of this new way of walking in year 1966, when Leena Jääskeläinem introduces the sticks as complement for walk in a physical education lesson. Aware of NW increases benefits respect traditional walk, she continues to recommend the sticks as a tool during walks in her lessons at University (6).

The first to write about the concept was Mauri Repo in the publication "Hiihdon Lajiosa" (TUL, 1979), translated: A part of cross-country skiing training methodic (5). A text that was used to promote Nordic walking (6), which is why it is considered the founder of the technique (5).

In the 90s it reached the general population as a recreational activity, thanks to Tuomo Jantunen, director of Suomen Latu (The Central Association for Recreational Sports and Outdoor Activities). In 1996, together with Matti Heikkilä, director of the Vierumäki Sports

Institute and Aki Karihtala laboratory, also vice president of Exel Oy, and the production director of this same company, Taisto Manninen, they developed special canes for this new way of walking (6).

At this time, the activity is officially named Sauvakävely, "walking with poles" in Finnish, later being replaced to "Nordic Walking" by Exel, a company that had developed the first specific pole that was registered under the name "Nordic Walker", in 1997 (6).

Aki Karihtala, founder of the International Nordic Walking Association, develops "How to introduce Nordic Walking Internationally", which mixes educational and informative data, with psychology, marketing and products, which reach the population through various means of communication, with the aim of promoting the new physical activity that was born. Finland is the first country where it is promoted, being expanded to other countries belatedly thanks to Exel. Finally, in 2000, the International Nordic Walking Association was founded, the first of the Nordic walking federations (6). In Spain, there is also a delegation from the International Nordic Walking Association (7).

In 2008 the World Record – Nordic Walking was born, in 2011 the Original Nordic Walking from Finland and in 2015 the European New Walking Organization (5).

3.1.1.2 Technique

Despite having emerged as a resource for summer times to replace cross-country skiing, the poles used for Nordic walking are shorter than ski poles, since the terrain conditions, without snow, are different. The recommendation about the length of the cane that each person should use is made based on the height, being as shown in Table 1 (8):

Table 1. Length of the Nordic walking pole depending on the person's height.

Height of person (cm)	Pole length (cm)
<150	105-110
150-160	115
161-170	120
171-180	125
181-190	130
191-200	135
>200	140-145

The poles must have an ergonomic grip that allows a correct technique to be performed, at a good pace and with quality of movement, a rigid and flexible stick, to absorb the impact, and light, and a tip specially designed to adapt to all types of of land (8).

There are different Nordic Walking techniques: ONW, INWA (both very similar and corresponding to the federation with which they share a name), FITTREK and KEENFIT, although they are all developed from the same basic technique. To make NW is needed to walk making the natural movement, alternating arms and legs, with the help of two NW poles. The right cane support is performed at the same time as the left foot support, and the left foot support is performed at the same time as the right cane support (9,10).

2.1.1.2 Benefits of Nordic walking

Carrying out a search in general terms in relation to Nordic walking, we find the following scientific evidence.

Nordic walking, as opposed to traditional walking without poles, has shown greater mechanical work and higher energy expenditure, however resulting in lower metabolic efficiency. This decrease in metabolic efficiency is a consequence of a greater involvement of the upper body musculature, which increases work production, as it is accompanied by isometric contractions and coactivations in the upper part of the body, resulting in the same displacement, hence the reduction of metabolic efficiency. The increase in mechanical work and energy expenditure is a consequence of the greater movement of the center of mass during walking (11).

Energy expenditure is comparable in walking without poles and Nordic walking, when both are performed uphill. Presumably this occurs because the effort is made by the legs in both activities. However, we can deduce that if we ask the subject to exert force with the sticks to propel themselves, energy expenditure will be increased, compared to the force exerted spontaneously (12).

It has also been shown to prevent joint damage in the lower extremities and reduce the load on the knee joints (13). This is due to the fact that the load is more evenly distributed throughout the body, transferring it to the upper part of the trunk (14).

It increases oxygen consumption compared to walking without poles, without increasing fatigue, therefore, it increases the benefits of exercise without putting more effort on the person (13). This is due to the involvement of the upper trunk muscles (12).

As for the gait, the step length is incresead and in relation to the support phase, the support time increases and the take-off time decreases. Since the support is accompanied by the support of the poles and the take-off is combined with the push of the poles against the ground.

Thanks to this, it can be beneficial for patients with pain in the lower extremities, arthritis or diabetes, reducing the problems resulting from plantar pressure (13).

Regarding muscular activity, an increase in the activity of the triceps and latissimus dorsi, involved in the support phase of the cane, has been seen. An increase in the activation of the biceps brachii and the anterior and middle deltoids is also observed, and throughout the gait cycle, since they are co-activated during the extension and support phase of the stick, with the triceps and the latissimus dorsi. While in the lower limbs no differences in activation are observed with respect to normal walking: rectus femoris, biceps femoris, tibialis anterior and gastrocnemius show similar activation (12,13). Greater activation of the anterior rectus has also been observed, to stabilize the entire gait cycle and decreased contraction of the erector spinae, the latter being remarkable, taking into account that overuse of it usually leads to lumbar pain, therefore NW is recommended in patients with lumbar or back pain in general (12).

A large part of the studies that exist to date on Nordic walking have the female population as the study sample. In these patients, a study by Park et al. (15). has shown that NW reduce low back pain, due to the coordination that occurs between the movement of the lower and upper extremities, which favours the neutral position of the spine. It develops simultaneously, as has already been commented, the upper and lower muscles. The paraspinal muscles are strengthened, which allows patients to maintain the spine in a correct position for a longer period of time, as the abdominal and pelvic muscles and the nervous system are working together.

Benefits have been shown in various pathologies and conditions that may be affecting the person's life, related to a training plan based on NW. For example, depression, which intervenes in the normal development of the life of those who suffer from it, increasing mortality and influencing the psychic, physical and social spheres. NW decreases the prevalence of depression and the depression score of the population studied, with a significant difference compared to walking without poles. It is therefore positioned as a psychologically stabilizing tool, an effective alternative for the treatment of depression and sleep disorders in the elderly (16).

Vílchez Barrera et al (17), perform a literature review on NW and its use in Physiotherapy. In it they analyze the results of studies on NW and: metabolic syndrome and obesity (18-23), musculoskeletal disorders (24-26), vascular disease (27,28), Parkinson's, chronic obstructive pulmonary disease (COPD) (4) and heart failure (29). The general conclusion of the review is

that NW should be incorporated as a form of safe physical activity in a physiotherapeutic context. Some of these articles will be analysed in detail below (17).

In the female population, Skórkowska-Telichowska et al. (2), also studied the effects on coronary artery disease, heart failure, arterial hypertension, hyperlipidemia, sarcopenia, peripheral arterial disease, metabolic syndrome and hip replacement. It is beneficial in sarcopenia, reducing the risk in women; for metabolic syndrome in early stages and non-diabetic patients. In post-hip prosthesis rehabilitation, it motivates patients to comply with it and helps maintain physical fitness and good musculoskeletal condition, important for improving quality of life.

To test the efficacy in hip osteoarthritis, the muscular activity around the joint and the movement of the pelvis have been analyzed after practicing NW. It is concluded that it can prevent hip pain and low back pain, in addition to preventing related disabilities (30). All this is achieved thanks to the decreased activation of the erector spinae, reducing its overuse, and of the abductor and adductor muscles of the hip, which reduces compression at the joint level (12,30).

The results obtained with Nordic walking on people with cardiovascular pathologies are analyzed in more detail below, since a high percentage of patients with chronic lung disease present some cardiovascular comorbidity (31).

The only meta-analysis on cardiovascular disease and Nordic walking analyzed articles on coronary artery disease, peripheral arterial disease, heart failure, and cerebrovascular accident (CVA) (32). In this case, Nordic walking alone is shown to be significantly more beneficial in combination with conventional cardiovascular rehabilitation in coronary artery disease (33,34). In the previously mentioned study by Skórkowska-Telichowska et al. (2), in coronary artery disease, Nordic walking shows significant improvements compared to normal walking in exercise capacity, measured in metabolic equivalents (METs), in dynamic balance and in fatigue. This improvement coincides with the improvement that occurs in exercise tolerance, strength and coordination of the upper musculature. It is therefore considered an effective and safe method.

In stroke, Nordic walking, performed on a treadmill like walking without poles, shows greater benefits than this and a significantly greater difference in the distance walked in the 6-minute walk test (6MWT) (35,36).

In the same way, Nordic walking has proven to be a useful tool for the non-pharmacological treatment of high blood pressure, with a significant drop in blood pressure after training of this

type, compared to the control group, studied in women of menopausal age and elderly with obesity. Its validity is concluded as an exercise that is intense enough, but not too vigorous, that induces favourable changes in blood pressure (2,37).

It has a beneficial effect in the prophylaxis of cardiovascular diseases, since it has shown greater efficacy in reducing body weight, blood glucose, total cholesterol, low-density lipoproteins, high-density lipoproteins, and triglycerides, in addition to greater adherence to the program , compared to a more conventional training performed with the Pilates method (2).

On the other hand, Nordic walking allows patients with heart failure to increase the intensity of exercise, and thereby safely increase cardiorespiratory benefits: it increases oxygen uptake, maximum heart rate, maximum systolic pressure and fatigue, without cardiac ischemia or significant arrhythmias. It is a safe, well-tolerated, and effective form of exercise that reduces the number of admissions to hospital for exacerbations (2). Also in heart failure, Nordic walking has been shown to improve oxygen consumption (VO2), respiratory exchange rate, expiratory volume, partial pressure of exhaled carbon dioxide, heart rate (HR), systolic blood pressure and the feeling of fatigue perceived according to the Borg scale, in relation to the group that walked without poles. This study also compared these results with the same protocol in two groups of healthy people, one for each training group, not obtaining changes in them (38).

Another study supports the use of Nordic walking in this type of patients, with heart failure. After 12 weeks of intervention, the 6MWT showed an 18% improvement in patients who performed Nordic walking, increased free time spent exercising, grip strength with the right hand, a predictor of heart health (39), and Hospital Anxiety and Depression Scale (HADS) data. However, there were no differences in VO2, left hand grip strength, body weight, and waist circumference (29).

It has been shown to be effective and safe, as no serious events such as exacerbations or hospitalizations has occurred, also performed independently in patients with heart failure, telemonitored by professionals. In this patients improves VO2, duration of workload in a cardiopulmonary exercise test , the distance walked in 6MWT and the quality of life according to the quality of life questionnaire (QoL-Q) (40).

In peripheral arterial disease (PAD) it manages to improve cardiovascular and functional health and quality of life (27,41–44). In addition, compared to traditional walking, improvements are achieved in exercise duration times and in peak oxygen consumption (VO2peak) (28).

Spaford et al. (27), carried out a study in which they compared Nordic walking with conventional walking in patients with PAD, showing no changes in claudication distance, which

was increased in both groups, as was the distance walked in the 6MWT; although it is true that these changes were greater in the Nordic walking group. Feelings of fatigue also improved more significantly in this group, which translated into these larger changes in distance walked (41). A significant improvement is also shown in the values obtained in the ankle-brachial index at the end of the initial tests with poles, compared to the same test without poles, as well as at the end of training, where both forms of training resulted in better marks. , but especially in the Nordic walking group. Calorie expenditure was also significantly increased compared to the control group. No differences were observed in pain, perceived fatigue or collaboration of the participants (27).

A year later, a complementary study to the latter was carried out, in which an attempt was made to analyze adherence in both groups one year after the sessions had ended: the Nordic walking group walked longer weekly and at a higher average speed, in addition, they exceeded adherence to exercise by 21% compared to the control group, which had stopped improving, reaching stability in the measurements. It is concluded in this study that a telemonitored program based on Nordic walking is more beneficial than one based on traditional walking (46).

A study - comparing a normal treadmill walking group with a NW group, and with a combined training group consisting of resistance training and NW - highlighted combined resistance training and Nordic walking as more beneficial , as it is the one that improves strength the most and also serves to increase the claudication distance. It is also highlighted that NW is attractive when it comes to ensuring compliance and motivation with the training schedule (47).

Another study confirms this greater increase in the distance walked in 6MWT after a Nordic walking protocol, in addition to a greater motivation of the patients, who walked further and faster (48). The latter is confirmed in the study by Collins et al. (28), in which the only parameter in which differences are observed is the duration of the walk, being greater in the Nordic walking group, and therefore being more beneficial for patients with PAD.

Regarding coronary syndrome, a clinical trial compared a control group with a traditional calisthenics training pattern and resistance exercise with a cycle ergometer, a Nordic walking group and a third group of walking without poles (both also combined with calisthenics). At the end of training, the energy expenditure measured with the accelerometer was significantly higher in the Nordic walking group, but not the energy expenditure measured with the frequency meter. Exercise capacity, measured in METs, was only increased in the two walking groups. In the rest of the tests carried out (Fullerton, 6MWT, and get up and go test) Nordic walking turned out to be the most beneficial training method (34).

On the other hand, in metabolic syndrome it has been shown that increases the ability to perform exercise measured according to VO2peak, body weight, body mass index (BMI) and decreases waist circumference, significantly in the group of study, respect to the controls, in those patients who showed a satisfaction with their training program equal to or greater than 80% (18). In addition, compared to resistance training, it has greater benefits when it comes to reducing the plasma atherogenic index and the score and prevalence of the population with metabolic syndrome, without making any changes in diet (19).

Regarding respiratory diseases, to our knowledge, COPD is the only one that has been studied in relation to walking training with poles, or Nordic walking.

Breyer et al. (4), in their study carried out in 2010, compared a training group with Nordic walking, performed at a heart rate of 75% of the initial maximum heart rate, 1 hour a day, three times a week, for 3 months , with a control group that did not receive any type of training. Nordic walking allows these patients to achieve the desired intensity of exercise, measured by heart rate, without difficulty and performing the technique correctly. Compared to the control group, daily physical activity was increased, as was the time spent walking and the time spent standing. The distance walked in the 6MWT was also greater, and the Borg scale used to measure dyspnea similarly showed benefits. Using the HADS scale, a significant improvement was observed in the patients who did Nordic walking at 3 months, and this improvement was maintained over time (9 months later), in the same way as quality of life, as opposed to the control group , whose data remained unchanged.

Lastly, in another study carried out with COPD patients, different training methods were compared: traditional supervised strength-resistance training versus combined and only supervised periodically training, consisting of Nordic walking, circuit training and aerobics classes (flexibility aerobic exercises and balance). The number of supervised sessions decreased every 5 weeks, starting at 3 sessions/week. From the 15th week to the 28th week, all the sessions were carried out without supervision. Each session consisted of 60 minutes in which the 3 types of exercise alternated. The Nordic walking sessions lasted between 10 and 20 minutes in which the participants walked at the speed chosen by them, taking into account that the perceived effort should be around 3-4 points on a Borg scale. The aerobic classes were dosed following the same intensity criteria, and consisted of exercises with free weight performed at 1 series of 8 repetitions. Finally, the training circuit, in which the participants performed different types of exercises, such as squats or lunges, performing between 10 and 15 repetitions per exercise, and doing between 2-4 laps of the circuit. Adherence was better

(100% vs. 87%) with alternative training compared to conventional training, although the difference was not significant. In all measurements, the results are comparable (49).

As for lung transplant candidate patients, Nordic walking, after a period of 12 weeks of training, shows a significant improvement in the 6MWT of 64 meters and in the Baseline Dyspnea Index (BDI). Nordic walking was performed in two cycles of 6 weeks each, of which the first 2 were performed in the hospital under the supervision of physiotherapists and the last 4 were performed by patients at home. During training, HR and oxygen saturation were measured. The recommendations followed to carry out this were those of the training guide for patients with COPD (50). The control group significantly worsened the values achieved on the Medical Research Council (MRC) scale compared to the study group. It is important to note that in this study the control group did not receive any type of training (51).

The same research group had previously carried out a study based on the same training, in which the distance covered in the 6MWT, the forced vital capacity and the forced expiratory volume in the first second (FEV1), quality of life and the degree of dyspnea as a variation in the MRC, BDI or in the oxygen cost diagram were analysed. The study group increased their walking distance by 6MWT, 51.6m on average. Of the spirometric values, only forced vital capacity (FVC) showed a significant improvement and the Short Form-Health Survey (SF-36) used to assess quality of life shows improvements in social and physical function, improvements that remain significant also 12 weeks after the rehabilitation program. No significant changes are shown in the degree of dyspnea (52).

2.1.2 Asthma and Physiotherapy

Within what is known as asthma there are various phenotypes that share similar clinical manifestations, but of different aetiology. It is considered a chronic inflammatory disease of the respiratory tract, in whose pathogenesis various cells and inflammatory mediators are involved, conditioned by genetic factors and that causes bronchial hyperresponsiveness and variable airflow obstruction, which may be totally or partially reversible: for medicinal action or spontaneously. Asthmatic exacerbation is caused by narrowing of the airway, which produces obstruction of the bronchial smooth muscle, edema and hypersecretion of the mucosa. Characteristic of this disease is bronchial hyperresponsiveness, defined as an exaggerated bronchoconstrictor response to a variety of physical, chemical, or biological stimuli (53).

The characteristic signs and symptoms of asthma are dyspnea, cough, wheezing, and chest pressure. These symptoms and signs are susceptible to seasonal variations and family and

personal history of atopy. Since they are symptoms common to other diseases, respiratory function tests must be performed for their diagnosis (53).

Asthma is classified based on daytime and nighttime symptoms, rescue medication, activity limitation, lung function (FEV1), peak expiratory flow (PEF) % predicted, and exacerbations, as shown in Table 2 (53). .

Table 2. Classification of asthma based on symptoms.

	Intermittent	Mild persistent	Moderate persistent	Severe persistent
Daytime symptoms	No (2 days or less a week)	More than two days a week	Daily	Continuous and several times a day
Reliever medication	No (2 or fewer days a week)	More than two days per week, but not daily	Every day	Several times a day
Nocturnal symptoms	No more than twice a month	More than twice a month	More than once a week	Frequent
Activity limitation	None	Something	Quite	A lot
Lung function (FEV1 or PEF) % predicted	>80%	>80%	>60-80%	≤60%
Exacerbations	None	One or none a year	Two or more a year	Two or more a year

FEV1: Forced expiratory volume in the first second; PEF: peak expiratory flow

An exacerbation is considered to be an acute or subacute episode characterized by a progressive increase in one or more of the typical symptoms (dyspnea, cough, wheezing, and chest tightness) accompanied by a decrease in PEF (53).

Asthma is boarded from Physiotherapy through two approaches: respiratory exercises (54-59) and physical exercise, generally aerobic (55,60). There are two recent systematic reviews that analyse asthma and its intervention from Physiotherapy in adult patients (61,62), and one aimed at analysing its benefits in the child population (63). One of the systematic reviews in adults is based on 21 clinical trials, of which only eleven are focused on therapeutic exercise

as a treatment for asthma (61). Of these eleven, six evaluate the improvement in quality of life in relation to exercise (64–69), obtaining a significant improvement in five of them (64–68). Regarding symptoms, they were analysed in three of the studies (64,65,70), with a significant improvement being observed in one of them (70) and an increase in symptom-free days in another (64). Only one of the studies assessed asthma control, which had improved significantly (66), and medication use decreased in one (67) of the three trials studied (65,67,68). VO2 improved in all the studies that analysed it (64,68,70), FEV1 only in one of the nine in which it was studied (71); FVC in one of the seven (71) and PEF in one of the four (72). It is concluded that therapeutic exercise can improve quality of life, decrease symptoms, and improve cardiopulmonary endurance and physical fitness, without changes in lung function (61).

Finally, the conclusions obtained in children are similar to those mentioned above: Physiotherapy, and specifically physical exercise and sports (such as basketball, swimming, cycling, exercise on a treadmill, exercise at home...), improve cardiorespiratory function (maximum consumption of oxygen (VO2max), oxygen pulse, ventilatory threshold) and its benefits on lung function are limited to PEF only. Regarding quality of life, a positive trend is shown, although the results are not conclusive (63).

There is another systematic review and meta-analysis, carried out exclusively with physical exercise as a therapeutic tool. All study groups improved symptoms compared to controls; regarding bronchial hyperreactivity, a significantly positive trend is shown in favour of the study group, as well as aerobic capacity measured according to VO2max, maximum workload and exercise resistance. Quality of life also improved significantly, although a meta-analysis of the data was impossible due to the variety of questionnaires used, as with symptoms (62).

Different studies have shown that aerobic exercise reduces bronchial hyperreactivity, proinflammatory cytokines, improves quality of life and exacerbation of symptoms in asthmatic patients (55). As well as improving VO2max, pulse oxygen, expiratory volume and maximum work rate. Regarding quality of life, there is a statistically significant improvement, although not high enough to exceed the threshold of clinical significance, and according to the Asthma Quality of Life Questionnaire (AQLQ), the improvement is also significant , but only in activities, emotions and general quality of life, not in symptoms (60).

2.2 Justification of work

As seen in the review of the literature carried out for the contextualization of this work, to date, there is no bibliography that demonstrates the validity of training based on Nordic walking in patients with asthma. That is therefore the objective of this work, through which it is intended

to obtain a possible training guideline for people diagnosed with asthma, which generates sufficient adherence and the necessary benefits to obtain an improvement in their physical condition, in their health and in their quality of life.

The possibility of carrying out this training, which we could refer to as treatment, is especially interesting, since it would be carried out in a context of green and blue urban environments, following the Urban Training® work model, which has shown better results than conventional care in patients with COPD and that also fits into the way of life of a Mediterranean culture (73), results that could be extrapolated to patients with asthma, given the similar characteristics of both pathologies: in both there is an airflow limitation (although reversible in the case of asthma), and similarly share symptoms, such as cough, dyspnea, and limited exercise tolerance (53,74).

3. Hypotheses and objectives

3.1 Hypothesis: null and alternative

Null hypothesis

Nordic walking does not present extra benefits than an educational plan in patients with asthma.

Alternative hypothesis

There are statistically significant differences between the application of a Nordic walking program and an educational plan versus the exclusive application of an educational plan in patients with asthma, in favour of first.

3.2 Research question

Does Nordic walking offer extra benefits compared to the exclusive application of an educational plan in patients with asthma?

3.3 Objectives: general and specific

General objectives

- To analyse whether there are statistically significant differences in relation to exercise tolerance and quality of life in people with asthma, when a training program based on Nordic walking is added to the usual procedure of an educational plan.
- To analyse the experience of patients regarding Nordic walking as a treatment for asthma.

Specific objectives

- To analyse whether there are statistically significant differences in people with asthma, when a training program based on Nordic walking is added to the usual procedure of an educational plan in relation to:
  - Exercise tolerance.
  - Quality of life.
  - Weekly physical activity.
  - Lung function.
  - Appearance and control of signs and symptoms.
  - Number of visits to emergency department.
  - Number of times that patients have to take rescue medicine through inhalers.
  - Adherence to treatment.
- To analyse, from a qualitative approach, the experience of the Nordic walking group in relation to:
  - How to deal with the disease.
  - Satisfaction with the intervention performed.
  - Perceived progress in the management of their disease after the intervention.

4. Methodology

4.1 Scope of study

To carry out this study, people diagnosed with asthma, of legal age, will be selected by those responsible for the Pulmonology area of the HM Modelo Hospital in A Coruña, and the Allergology and Pulmonology areas of the Complex University Hospital of A Coruña (CHUAC). Basing on the inclusion and exclusion criteria, patients will be offered the possibility of participating in the study. The patient will be who contacts research team after being selected.

Once the participants have been chosen, they will be randomly distributed into the control group or study group.

4.2 Study period

The study period runs from December 2018, when this project begins, to March 2024, when the investigation is planned to be completed.

4.3 Type of study

A single-blind randomized clinical trial, in which only the person who performs the evaluations and the one who analyses the data do not know to which group they belong.

4.4 Selection criteria

Inclusion criteria:

- People over 18 years old.
- Asthma diagnosis.
- Desire to participate in the study.
- Ability to sign informed consent.

Exclusion criteria:

Contraindications for cardiovascular exercise according to the American Heart Association (AHA) (75), contraindications for performing a 6MWT according to the American Thoracic Society European Respiratory Society (ATS/ERS) (76), spirometry according to SEPAR criteria (77). All of them are available in Appendix 1. The following criteria are added, which are those used in the study carried out with Nordic walking and patients with COPD (4), and those of a study carried out following a training protocol in patients with asthma (66):

- Contraindications for performing the 6MWT according to the ATS/ERS (Appendix 1)
- Contraindications for performing a forced spirometry according to Separ criterio (Appendix 1).
- People with other respiratory conditions besides asthma.
- Asthma exacerbation in the last 12 weeks. Exacerbations are defined as episodes of worsening of the patient's baseline situation that require modifications in treatment. They are identified by changes in symptoms, reliever medication, or lung function relative to the daily variation for a particular patient. Retrospectively, they could also be identified by an increase in the maintenance treatment dose for at least 3 days. In Appendix 1 of contraindications there is also a classification table of the severity of the asthmatic exacerbation (53).
- Acute myocardial infarction in the last 6 months.
- Cardiac arrhythmias greater than grade IIIb on the Lown scale.
- Gait disorders due to problems of the musculoskeletal system.
- Respiratory infection in the last 4 weeks.
- Smokers.
- Former smokers less than 2 years.
- Comorbidities involving reduced ability to exercise: significant anemia, electrolyte imbalance or hyperthyroidism.

- Current participants in moderate or vigorous exercise sessions of more than 30 minutes/day.
- Participation in a pulmonary rehabilitation program in the past 12 months.
- Pregnant and lactating women.

4.5 Sample size justification

The sample size is selected based on the distance walked in the 6MWT as it is considered the main outcome variable of the study. Knowing the data of the minimum clinically relevant difference (MCID), 30.5m (78) and using the standard deviation data from the study by Coelho et al. (79), in which subjects with asthma presented a standard deviation of 41.5m on the 6MWT, for a bilateral hypothesis, introducing a significance of 0.05, a statistical power of 80%, and assuming a 10% loss throughout the training period, 34 subjects per group are required. To calculate the sample size, the tool created by the CHUAC Clinical Epidemiology and Biostatistics Uni from CHUAC was used. (https://bit.ly/2JsRUzh).

The calculation of the sample size (n=68) has been carried out to guarantee that despite the fact that there are losses to follow-up (estimated at 10%), the study has sufficient power to observe statistically significant differences between the groups (if any). In addition, the impact of loss to follow-up will be evaluated a posteriori by comparing the baseline characteristics of patients who do not complete the study and those who complete it as planned.

4.6 Sample selection

The letter of invitation to participate will be sent to the heads of the Pulmonology and Allergology service of the CHUAC and of the Pulmonology service of the HM Modelo Hospital, so that they can inform their patients about the performance of this study during a routine visit to the consultation in their reference hospital. Those responsible for each area, collaborating researchers in this study, will recruit the participants. Dr. Larissa Patricia Fontán García Boente as head of the Pulmonology area at the HM Modelo Hospital, Dr. Marina Blanco Aparicio as head of Pulmonology at CHUAC and Dr. Manuel Jorge Rial Prado as head of the Allergology area at CHUAC.

A person outside the research will distribute the participants into both groups using a randomization computer program.

4.7 Description of the variables

The study variables are detailed below, summarized in Table 4.

4.7.1 Exercise tolerance

The 6MWT will be used to measure exercise capacity and associated respiratory distress. It is a test validated by the American Thoracic Society (76) with a known MCID of 30.5 meters (78).

4.7.2 Daily physical activity level

The tools used to objectify the changes achieved in this aspect will be:

- International Physical Activity Questionnaire (IPAQ) short version, validated questionnaire (80), in its Spanish version.
- Number of steps. Patients should note the value of the diary app in their patient diary (Appendix 2).

4.7.3 Quality of life

Regarding quality of life, three questionnaires will be used to analyse the changes achieved in said variable:

- SF-36, validated questionnaire (81), also in Spanish (82), with MCID, classified according to its significance (low, moderate or high significance), in its 8 sections. Table 3 (83).

Table 3. Minimum clinically significant difference classified by significance and by sections of the Short Form - Health Survey.

	Low significance	Moderate significance	High significance
Physical function	10	20	30
Limitation due to physical problems	12,5	25	30
Body ache	10	20	37,5
General health	10	20	30
Vitality	12,5	25	37,5
Social role	12,5	25	37,5
Emotional	16,7	33,3	50
Mental health	10	20	30

- *Asthma Quality of Life Questionnaire (AQLQ), questionnaire validated in patients with asthma (84), as well as its version in Spanish (85), with known MCID: 0.5 point difference (86).*

4.7.4 Asthma control and symptoms

For asthma control, the Asthma Control Test (ACT) questionnaire will be used (87), with a mean MCID of 2.2 (88) and validated for use with the Spanish-speaking population (89). The Test of Adhesion to Inhalers (TAI), a questionnaire validated in Spanish (90), will also be used. Regarding the symptoms, the characteristics and intensity of these and the days that the patients have spent without symptoms will be analyzed through the same diary used for the previous measurements (Appendix 2).

4.7.5 Dyspnea

The Borg scale (91) and the Modified Medical Research Council (mMRC) (92) scale will be used.

4.7.6 Pulmonary function

Pulmonary function should be measured regularly in asthmatic patients (93), using forced spirometry, with which we will obtain the values of FEV1 and FVC, as well as the ratio between both parameters.

The PEF is used to measure the variation or fluctuation of symptoms and lung function over time, that is, the characteristic variability of asthma. The patient will measure it daily, with a portable PEF meter, and must record its value in the symptom diary (Appendix 2) (53,94).

4.7.7 Respiratory muscle strength

The maximum inspiratory pressure (PIM) and the maximum expiratory pressure (PEM) will also be measured.

4.7.8 Muscular strength-endurance

A grip strength measurement will be made with a dynamometer (Jamar Dynamometer) (95). This measurement has been validated as useful and reliable for assessing muscle strength in a clinical setting (96). Grip strength is related to short-term mortality and morbidity, with the risk of hospitalization, re-hospitalization, post-surgical complications, loss of independence, functional limitations, and is a marker of the patient's nutritional status (97).

4.7.9 Medication taking

Through a diary that will be provided to the patient (Appendix 2).

4.7.10 Emergency visits

Through a diary that will be provided to the patient, and in relation to the previous section (Appendix 2).

4.7.11 Adherence

Through a diary that will be provided to the patient (Appendix 2).

Table 4 shows the study variables chosen for this clinical trial and the measurement instrument corresponding to each of them.

Tabla 4. Variables de estudio e instrumentos de medida.

STUDY VARIABLE	MEASURE INSTRUMENT
xercise tolerance	6 minute walk test (6MWT)
Physical activity level	International Physical Activity Questionnaire (IPAQ)<br>APP (step counter)
Quality of life	*Short-form Health Survey*<br>*Asthma Quality of Life Questionnaire*
Asthma control and symptoms	*Asthma Control Test*<br>Inhaler adherence test<br>Patient diary
Dyspnoea	Borg scale<br>Medical Research Council modified
Pulmonary function	Spirometry<br>Peak Flow Meter
Peak respiratory pressures	PIM and PEM meter
Muscular strength-endurance	Dynamometer
Medication taking	Patient diary
Emergency visits	Patient diary
Adherence	Patient diary

4.7.12. Qualitative data

Qualitative data will be collected from a semi-structured group interview in focus group format, with the aim of achieving a better understanding of the participants' experience with the proposed activity (Nordic walking), and if possible, compare these qualitative findings, relating and completing them, with quantitative information, thus providing a comprehensive view between both approaches, through a mixed-method study.

4.8 Measurements and intervention

4.8.1 Measurements

All measurements will be carried out by the same physiotherapist previously trained for it. Pre- and post-treatment will be carried out, likewise, 3 and 6 months after the last session, in order to evaluate the effects in the medium and long term.

4.8.1.1 Personal interview and physical examination

A personal interview will be carried out, whose data collection notebook can be consulted in Appendix 5, which contains the following information:

- Sociodemographic data: age, sex, nationality, employment status.
- Smoking habits.

- Pathological history.
- General health.

In the physical examination, the anthropometric measurements will be recorded with a scale with a height rod (Seca 700, Hamburg, Germany), with the subject barefoot, head erect, weight distributed on both heels and with empty pockets. At that time, the rest of the measurements of this research will also be carried out: exercise tolerance and quality of life questionnaires, 6MWT, spirometry and grip strength measurement; and patients will be instructed to correctly cover their patient diary (Appendix 2), information that will be reinforced during the educational plan class.

4.8.1.2 Exercise tolerance

6 minute walking test

The 6MWT will be performed, according to the indications of the guide for carrying out the ATS/ERS test (76). It will take place in an interior corridor 30 meters long delimited by two cones located at a distance of 29 meters (leaving a space of 0.5 m on each side for turns). Participants will be encouraged to "walk as fast as possible" without running. They must not talk or be distracted during the course, and if necessary, they will be instructed that they can stop or slow down, but that at no time will the stopwatch stop counting.

Vital signs (HR, oxygen saturation and respiratory rate) will be taken, as well as the feeling of fatigue and dyspnea according to the Borg scale, before and after the test. The number of stops, their total time and their reasons will be recorded. The meters traveled will also be recorded. At the end of the test, vital signs will be monitored for the first 5 minutes to check that the patient is recovering properly. Two tests will be carried out with an intermediate interval of 30 minutes of rest. All these data are collected in the registration sheet (Appendix 6).

4.8.1.3 Physical activity level

IPAQ

We will use the IPAQ questionnaire in its short version (Appendix 7), which will be administered through a personal interview. The results will be analyzed according to the guidelines of the IPAQ research committee (98).

APP (STEP COUNTER)

A mobile application will be used in charge of counting the steps on the patient's mobile, to count the steps they take in their day to day. Patients will be instructed in its use and must write in their diary the steps performed each day (Appendix 2).

4.8.1.4 Quality of life

The SF-36 (Appendix 8), AQLQ (Appendix 9) quality of life questionnaires will be administered to the subjects through a personal interview.

4.8.1.5 Asthma control and symptoms

The ACT scale (Appendix 10) will be used to assess the control of symptoms presented by the patient. The Inhaler Adherence Test will be used to evaluate their intake (Appendix 11).

In addition, in the patient's diary (Appendix 2), the patient must record information regarding dyspnea (according to Borg's scale), expectoration, cough, wheezing (sensation of whistling in the chest), medication intake and visits to the emergency room, as an instrument of assessment for symptom control.

4.8.1.5 Dyspnea

To assess dyspnea, the mMRC scale will be applied during the personal interview, (scale that appears in Appendix 12). In addition, before and after exercise, questions will be asked about the sensation of dyspnea according to the modified Borg scale. During the exercise, you will be asked in the same way to graduate the dyspnea according to the same scale, every 5 minutes elapsed. This scale appears as part of Appendix 13.

4.8.1.6 Lung function

To carry out the spirometries, the international recommendations of the ATS/ERS will be followed and they will be carried out with a Dadospir® 120C spirometer (Sibel Group, Barcelona, Spain), and the FEV1, FVC and FEV1/FVC data obtained after a forced spirometry. A disposable mouthpiece will be used which must be in place and well-sealed around the patient's lips. From the functional residual capacity (FRC), the patient will be asked to perform a rapid, but not forced, maximal inspiratory manoeuvre. Next, and after an apnea of less than 1 second in total lung capacity, maximum, rapid and forced expiration will be requested until the lungs are completely emptied (99).

The patient must, at their own expense, perform a PEF measurement on a daily basis, with a Peak Flow meter (asmaPLAN+, Vitalograph®) attached to a tube mouthpiece. Patients will be instructed that for this measurement they must exhale all the air out of the device up to the expiratory reserve volume, then inhale deeply until TLC, insert the mouthpiece sealing the lips well around it and exhale as forcefully as possible at through the device. This measurement should be performed every day at the same time (when getting up in the morning) and under the same circumstances, a total of 3 times, noting in the patient's diary (Appendix 3), only the highest of these values (100).

4.8.1.7 Respiratory muscle strength.

PIM and PEM will be measured following national SEPAR recommendations (101) and using the Micromedical RPM Carefusion device connected to PUMA® software. The measurement of maximum respiratory pressures is one of the few evaluations of lung function that presents disagreement between national and international scientific societies on its protocol (76,101). We have opted for the application of the protocol proposed by SEPAR since it has been shown that higher values of PIM and PEM are obtained with it (102). The participants will be placed in the same position as in the case of spirometry. As additional material, a rigid material diver-type mouthpiece will be used, with a washer to place between the inner face of the lips and the gums, preventing air leaks, as well as nose clips. Before carrying out the tests, the patient must be relaxed and seated comfortably for 5 minutes, a moment that we will take advantage of to give the appropriate explanations. It will start by measuring the PEM as it is easier to understand and execute. It is important to bear in mind that between the evaluation of both pressures, there will be a 5-minute rest, and between the performance of maneuvers of the same type, a one-minute rest will be given. Up to a maximum of 10 attempts will be made to obtain 6 technically acceptable maneuvers, which means that they have lasted between 3 and 5 seconds, that there are no evidence of air leaks and that there is a tendency to plateau in the curve of both pressures. In addition, 3 reproducible maneuvers will be sought among the 6 acceptable ones (that is, with a variability of less than 5%). Among the 3 acceptable and reproducible maneuvers, the one with the highest value, measured in cmH_2O (101), will be selected. The results obtained will be compared with the reference values for the healthy adult Spanish population proposed by Morales et al. (103). According to the lower limits of normality collected in the literature (104) we will consider the existence of muscle weakness when the PIM and PEM are below 80% to 65% of the reference value.

4.8.1.8 Fuerza de agarre

A measurement of grip strength will be made with both hands, through the use of a dynamometer (Jamar Dynamometer). For the measurement, the dynamometer cuff will be

placed in the second smallest position, and the second phalanges should rest around it. If not, the position will be readjusted. First, the patient will see a demonstration of the measurement carried out by the physiotherapist in charge of it, who will squeeze a small ball. Afterwards, the patient will try himself to squeeze said ball (95). Subsequently, the patient will carry out the movement himself on the dynamometer, in a sitting position, with the arm close to the chest, neutral rotation, elbow flexed at 90 degrees and forearm and wrist in neutral position (105). The best result of three measurements made with each hand, measured in kilos, will be recorded. The physiotherapist must be sure that the patient's effort is the maximum, looking for it in his facial expression, contraction of the muscles of the arm and forearm, coloration of the phalanges, which will be white, and a concordance between the three measurements (86).

4.8.1.9 Medication taking

The patient must record in his patient diary (Appendix 2) when, what and in what quantity he needs to administer medications to relieve symptoms.

4.8.1.10 Emergency visits

The patient must also write down in the patient's diary (Appendix 2) the visits to the emergency room that he or she makes.

4.8.1.11 Adherence

Adherence to training will be assessed after analyzing the data that the patient has collected in his symptoms diary (number of steps taken per day) and the results of the IPAQ questionnaire.

4.8.1.12 Qualitative data

In order to analyze the experience and satisfaction of the participants with the Nordic walking intervention, the group that performs Nordic walking will be summoned to conduct an interview in focus group format, together with the members of their training group, once finished this. Therefore, each focus group will be made up of 5 people (the 5 members of each Nordic walking group), and during them, semi-structured open-ended questions will be asked (these can be consulted in Appendix 15). Participants will be able to respond freely and interacting with each other. The focus group interview will be conducted by an expert in this type of qualitative methodology and will take place at the Faculty of Physiotherapy of the Universidade da Coruña, with a duration of approximately one hour (it will be considered finished when the information is saturated). Another assistant will participate in the sessions passively, taking charge of taking notes. These two people will start the meeting by introducing themselves and giving clear information about the purpose of the meeting. The participants, who will have

signed the informed consent for this, will be recorded on video and audio, for the subsequent transcription and analysis of the data collected during these interviews.

4.8.2 Intervention

Patients will be scheduled initially for a clinical interview and for all measurements and tests to be carried out.

All participants, study group and control group, will be instructed in two classes of two hours each, in an educational plan aimed at knowing and managing their pathology, as well as providing education to patients regarding healthy lifestyle habits. The outline of the educational plan is that of table 5, and can be checked in Appendix 14. All participants will be given an explanatory guide on their disease and with indications and advice for its management (Appendix 16).

Table 5. Educational plan for the participants.

General information about asthma	What is asthma? Definition and description<br>Prevalence<br>Asthma phenotypes: “types of asthmatics”<br>asthma management
Medication	What? Types of medications<br>How? Correct administration of inhalers
Control of the environment and lifestyle	optimal environment<br>Nutrition<br>Physical exercise and active life
Asthma care techniques	
Asthma guide	

Only the study group will undergo a training program, detailed in Table 5. This program is based on the ATS/ERS recommendations for pulmonary rehabilitation (106), as well as on the study by Breyer et al (4), in which the effects of training based on Nordic walking in patients with COPD were analysed, since there is no literature on Nordic walking and asthma; and in the guide by Garber et al.(107), for the prescription of exercise.

The necessary material for the development of the session will consist of a pair of Power Poles Nordic walking poles (Leki; Hamburg, Germany) (4), and a Polar® H7 beat heart rate watch, to control the intensity of the exercise, which between 50 and 75% of the theoretical maximum heart rate (HRmax) and between 4 and 6 on a Modified Borg Scale for fatigue and dyspnea, intensity at which physiological adaptation to exercise occurs, for which also a minimum of 20 sessions is necessary, so since the recommended frequency is 3 sessions/week, we will need a minimum of 7 weeks. We will set a total of 8 weeks, or 2 months (106).

The training plan appears summarized in table 6, and can be checked in Appendix 17.

Table 6. Training plan.

	Time	Type of exercise
Heating	5 minutos	Joint mobility / dynamic stretching.
	10 minutes	Walk at a light pace.
Central part	30 minutes	Initially 50% of theoretical HRmax<br>Intensity is increased by 5% per week until reaching 75% of the theoretical HRmax.
Back to the calm	10 minutes	Stretching
	5 minutes	Relaxing exercises
3 sessions/week		
Total duration: 8 weeks		

The study group will be instructed as a group in a previous Nordic walking session. The remaining sessions will also be group sessions. The groups will consist of 5 patients each.

The sessions will take place on the promenade of A Coruña and through the old town and the Marina, an asphalted terrain and without too many changes in level, and will be carried out by a physiotherapist, trained in Finland to put Nordic walking into practice.

If necessary, the patient will use the usual rescue medication for asthma exacerbations.

The measurements will be carried out by a person other than the person who carries out the intervention and who indexes and analyzes the data, before the intervention and at the end of it. In addition, the same follow-up measurements will be made 3 and 6 months after the last session.

4.9 Statistic analysis

A descriptive analysis of the variables included in the study will be carried out. The qualitative variables will be expressed with their absolute values and percentages. From the quantitative variables, their mean, standard deviation, median, quartiles and maximum and minimum values will be calculated.

For the analysis of the result variables, a repeated measures ANOVA or a Kruskal-Wallis test will be carried out, as appropriate, based on the normality of the data distribution, which will be verified through the Shapiro-Wilks statistic.

Qualitative data will be analyzed using thematic analysis. Two researchers will carry out an in-depth reading of the transcripts and will take notes throughout the text, to generate initial codes. Afterwards, the text will be reread, based on these codes, to search for and review related topics, in order to contextualize and conceptualize them. Then a report can be generated. Another member of the team will collate the data, to ensure the veracity of the findings, and through the "peer debriefing technique" (108), will make their colleagues aware of their interpretations, with the ultimate goal of cleaning the process of preconceived assumptions and wrong assumptions. The absolute and relative frequencies of the information of each theme will be analyzed. The agreement between evaluators will be evaluated through percentages of agreement, calculated as the number of thematic units in which they coincide, divided by the total units measured. Cohen's index will also be used, considering a value >0.81 as an acceptable level of agreement (109).

The data analysis will be carried out with the SPSS program, version 26.00.

5. Timeline and stages of development

Table 7. Schedule and work plan

	**Dates**
**Project Design**	December 2018 - July 2019
**Drafting of the manuscript**	December 2018 – July 2019
**Ethics Committee**	December 2019 – July 2020
**Sample selection**	August 2021 – September 2023
**Field work**	June 2022 – November 2023
**Focus groups**	October 2022 - December 2023
**Analysis of the results**	December 2023 – March 2024
**Dissemination of results**	March 2024 – November 2024

Due to the difficulty of recruiting people with asthma, recruitment and intervention will be carried out in parallel. For every 5 people recruited, a group will be formed that will start with the intervention, regardless of not having recruited the entire sample.

5.1. Project design

The design of the project, as well as the writing of the manuscript, were carried out by Ms. María Vilanova Pereira, with the collaboration of Ana Lista Paz, in the period of previous documentation: context of the study and existing information on the subject.

5.2 Ethics Committee.

The manuscript was sent to the Clinical Research Ethics Committee of Galicia on November 4, 2019, and was approved on July 20, 2020. After acceptance, it is forwarded for project monitoring and validation of its addenda.

5.3 Sample selection

Una vez obtenida la aprobación para el proyecto, se procederá a la selección de los sujetos que participarán en el proyecto (68 en total; 34 para el grupo de estudio y 34 para el grupo control)

5.4 Investigation Development

Once the sample has been selected, Ana Lista Paz will start carrying out the previous measurements, and María Vilanova Pereira, who will carry out the educational and training plan, who has been trained in Nordic walking.

5.5 Analysis of the results.

Once the interventions and evaluations, both quantitative and qualitative, will proceed to the analysis of the results, seeking the joint understanding of both types of data, through a mixed method approach.

5.6 Dissemination of results.

The conclusions obtained will be disseminated through the means described below and by the hand of three members of the research team: Dr. Ana Lista Paz, the professor and the physiotherapists María Vilanova Pereira and Margarita Barral Fernández.

6. Ethical-legal aspects.

Once the drafting of this project has been completed, it will be sent to the Ethics Committee for Clinical Research (CEIC) of Galicia, for approval that allows the development and dissemination of its results. After its approval, the protocol is re-sent, with follow-up information and validation of an addendum, for a qualitative approach to the study.

This clinical trial is accepted and will comply with the standards of Good Clinical Practice (ICH) and the Declaration of Helsinki (Brazil 2013).

Truthful and understandable information will be provided to the participants of this project, about the objectives of this study, the tests that we are going to carry out, as well as what their contraindications are, and the possible complications that may arise during them. This information is provided verbally and in writing, together with the corresponding informed consent (Appendix 4). Said informed consent was prepared in accordance with the provisions of article 8 of Law 41/2002, of November 14, regulating basic patient autonomy and rights and obligations in terms of information and clinical documentation.

In accordance with the provisions of article 7 of Law 41/2002, as well as in European Regulation 2016/679 and in Organic Law 3/2018 on the Protection of Personal Data and guarantee of digital rights, confidentiality will be strictly respected. of the personal and health data of the participants.

In relation to the protection of personal data, as can be seen in the data collection notebook (Appendix 5), no identification data will be collected from the participants (name, surname,

address, contact telephone number, DNI will not be collected). , etc). Participating subjects will be pseudonymous with an identification number assigned by the principal investigator (PI) of the study (NUMID).

The audio and image recordings obtained during the group interviews will be saved by the main researcher in an encrypted file on a computer at the Faculty of Physiotherapy to which only she and the people who make the transcripts will have access. The transcripts will be made by pseudonymizing the personal data that may be mentioned during the sessions.

Once the study is finished, the information will be stored exclusively in digital format, and guarded by the data protection delegate of the University of Coruña, Luz María Puente Aba, being stored in an encrypted folder, on a computer with a personal password to which only she will have access. To contact it: email dpg@udc.gal or by phone 881 01 16 05 and 881 011 61.

The PI of the project and the secondary researchers, María Vilanova Pereira (Col. 3524), Ana Lista Paz (Col. 2122) and Margarita Barral Fernández (Col. 3547), have civil liability insurance provided by the College of Physiotherapists of Galicia (COFIGA). The insurance has a civil liability policy (with a limit of €601,012.10 per claim, year and collegiate), which covers damage caused by work activity to third parties. In addition, the Spanish Association of Physiotherapists, of which COFIGA is in turn a member, defines physiotherapists in its statutes as "that person, who, being in possession of the Official Title of Physiotherapist, can develop any facet of his profession in the teaching, care, research and management fields, using, for this, the knowledge acquired in their curriculum." In addition, in ORDER CIN/2135/2008, which regulates training in the degree in Physiotherapy, in section 3, point 15, research competence is mentioned as one of the competences achieved in the degree.

7. Applicability of the study

The results of this study will allow, in the first place, to know the effectiveness of an alternative therapy to conventional ones and perfectly adaptable to people's lives, based on Nordic walking, for subjects with asthma. As it is an unknown activity and little carried out in our geographical and cultural context, it will also help us to assess the acceptance of this exercise in society, being able to open up new methods of training and therapeutic exercise in the future. Exercise with, presumably, more effectiveness than conventional walking, which is perfectly accepted in society.

Finally, it allows us to open a new line of research in our community: Nordic walking. If the benefits of Nordic walking are proven, patients with asthma could be approached in a new way. It would be a low-cost treatment to develop and would allow the health system to save costs, if exacerbations, medications, visits to the health centre, etc. are reduced. Therefore, the cost-effectiveness of Nordic walking in respiratory diseases could be analysed.

8. Results dissemination plan

8.1 Congresses

It is planned to present the results at the SEPAR National Congress in 2021. This congress is defined as a meeting point in which the achievements of clinical activity and research each year are shown, in addition to having an important social role. , disseminating among the public the most important aspects of pulmonology and thoracic surgery.

In the same way, the Congress of the Spanish Association of Physiotherapists would be of interest to us, a congress that represents and makes visible the value of national Physiotherapy and that serves as a meeting point for professionals and organizations from all over the territory.

Likewise, at the international congress of the European Respiratory Society, which defines itself as a showcase of excellence in the field of respiratory medicine.

8.2 Journals

An attempt will be made to have the results published as a scientific research article in journals related to Physiotherapy and the health field: Archivos de Bronconeumología, SEPAR's organ of scientific expression, (impact factor 2.979; quartile 2); in the European Respiratory Journal (impact factor 12.242, quartile 1), Physiotherapy (impact factor 1.085, quartile 1), Chest (impact factor 2.591, quartile 1). In addition, to publicize this modality, part of the results will be published in the AEF journal, Physiotherapy (SCImago Journal Rank-SJR:0.127).

9. Economic memory

9.1 Necessary resources

Table 8 lists the material necessary to carry out the study, as well as its cost.

Tabla 8. Material necesario.

NECESSARY MATERIAL	UNITS	PRICE (with VAT)
Inventoriable material		
Spirometer Datapir ® 120c	1	1.839,33€
S3000-3L Calibration Syringe	1	417,56€
Oregon Scientific ® Digital Weather Station	1	45,13€
Nasal forceps	1	1,71€
Scale and height rod	1	140€
Jamar Dynamometer	1	526,63€
Littman 3200 Electronic Stethoscope	1	502,73€
HYLOGY® upper arm blood pressure monitor	1	26,99€
Onyx® 9500 Finger Pulse Oximeter	1	399,30€
Asmaplan Vitalograph Peak Flow Meter	68	1.394€
Polar® H7 beat heart rate monitor	5	575,63€
Decathlon Nordic Walking Poles	12	95,88€
Consumables		
1 box of 100 antibacterial filters	1	165,77€
Thermal printer paper spirometer (10 units)	1	21,78€
Other expenses		
Attendance at SEPAR congress	1	784€
Travel SEPAR congresses	1	250€
Hotel night – SEPAR Congress	3	201€
Maintenance allowances – SEPAR Congress	3	111€
AEF congress attendance	1	190€
AEF congress travel	1	250€
Hotel night - AEF Congress	3	201€
Maintenance allowances – AEF Congress	3	111€
ERS congress attendance	1	625€
ERS congress displacement	1	400€
Hotel night – ERS Congress	3	201€
Maintenance allowances – ERS Congress	3	111€
Proofreading	2	200€
Printing costs®	1	300€
		Total cost
		13.048,76€

9.2 Possible funding sources

For the realization and subsequent dissemination of the results obtained with this study, funding will be sought through different institutions and organizations.

- “Ayudas respira”. SEPAR allocates €20,000 each year in grants to professional nurses or physiotherapists for the presentation of communications at the SEPAR National

Congress. These grants cover travel and accommodation expenses of those interested, to facilitate their attendance at said congresses.

- COFIGA research grants. COFIGA allocates €5,000 in aid, to be distributed among a total of 5 researchers. Granted.
- SEPAR research grants. Every year SEPAR opens a call for endowed scholarships of 18,000 euros in the case of Physiotherapy, which we can opt for with this project. Granted.
- SEPAR Scholarships. They are aimed primarily at SEPAR members who have recently completed their clinical training as residents in Pulmonology or Thoracic Surgery, or for University Diplomas and Graduates in Nursing and Physiotherapy, who wish to train in the field of respiratory research, developing a scientific research project .

10. Available means and research team to carry out the proposal.

To carry out this project we have the support of the Faculty of Physiotherapy of the Universidade da Coruña, which will make available to the research team an adequate space to carry out the field measurements, in which the privacy of the participants is guaranteed. the participants. To carry out the Nordic walking training, the green and blue spaces of the city of A Coruña will be used.

10.1 Research team experience.

Regarding the human resources available, the IP of the project, the physiotherapist María Vilanova Pereira, who will carry out the training with Nordic walking. This professional has made a stay at the University of Applied Sciences of Savonia (Kuopio), located in Finland, cradle of Nordic walking. During the same she has done a training as a Nordic walking instructor in the Suomen Latu. María Vilanova Pereira will also be in charge of carrying out the health education plan for the participants, for which she will be previously trained by the study's collaborating researcher, Ana Lista Paz.

In addition, Professor Ana Lista Paz has extensive experience in conducting research studies that involve measurement of lung function variables, strength and resistance of the respiratory muscles, and exercise tolerance. Examples of this are the studies: "Comparative analysis of lung function in subjects with and without hemiplegia/chronic hemiparesis", which has already given scientific production, and the multicenter study entitled "Determination of the reference values of maximum respiratory pressures and nasal pressure in maximum inhalation in a healthy adult Spanish population”, currently under development.

The recruitment of the study subjects will be facilitated by the heads of the CHUAC Pneumology and Allergology service, and the patients of the Pneumology consultation of the HM Modelo. The randomization to the groups will be carried out by the Physiotherapist Margarita Barral Fernández.

The subjects must read the patient information sheet (Appendix 3) and sign an informed consent, available in Appendix 4, after being informed by their doctor about the objective and the intervention of the study, as well as the measurements that will be taken. will carry out for the realization of the same.

Finally, in the statistical analysis of the data we will have the participation of Professor Alejandro Quintela del Río, an expert in statistics and with extensive experience in biosanitary data analysis.

10.2 Material resources available

The Faculty of Physiotherapy of the UDC makes the following material available for this project:

- Spirometer Datapir®120C.
- S3000-3L calibration syringe.
- Scale and height rod.
- Jamar Dynamometer.
- Littman 3200 Electronic Stethoscope.
- Pulse oximeter
- arm blood pressure monitor
- Polar® watches.
- Consumables.

It will not be necessary for the research team to cover these expenses, therefore, the material budget (without taking into account dissemination expenses) is reduced from €8,415.63 to €2,119.38.

Lastly, the Faculty of Physiotherapy of the Universidade da Coruña has the capacity to access scientific information through the different Health Sciences databases, and has access to the statistical package necessary to carry out the statistical analysis of the data.

11. Bibliography

1. What is Nordic Walking? [Internet]. International Nordic Walking Federation. 2019. Disponible en: http://www.inwa-nordicwalking.com/what-is-nordic-walking/

2. Skórkowska-Telichowska K, Kropielnicka K, Bulińska K, Pilch U, Woźniewski M, Szuba A, et al. Nordic walking in the second half of life. Aging Clinical and Experimental Research. 2016;28(6):1035-46.

3. Bombieri F, Schena F, Pellegrini B, Barone P, Tinazzi M, Erro R. Walking on four limbs: A systematic review of Nordic Walking in Parkinson disease. Parkinsonism & Related Disorders. 2017;38:8-12.

4. Breyer M-K, Breyer-Kohansal R, Funk G-C, Dornhofer N, Spruit MA, Wouters EF, et al. Nordic Walking improves daily physical activities in COPD: a randomised controlled trial. Respiratory Research [Internet]. 2010 [citado 1 de diciembre de 2018];11(1). Disponible en: http://respiratory-research.biomedcentral.com/articles/10.1186/1465-9921-11-112

5. World Record-Nordic Walking. Nordic Walking history. [Internet]. World Record-Nordic Walking. 2018. Disponible en: https://www.wr-nw.com/nordic-walking-history.html

6. International Nordic Walking Asociation. History of Nordic Walking [Internet]. International Nordic Walking Association. 2018. Disponible en: http://www.inwa-nordicwalking.com/inwa-history/

7. International Nordic Walking Asociation Spain. INWA SPAIN [Internet]. International Nordic Walking Association (INWA) Spain. [citado 12 de diciembre de 2018]. Disponible en: http://www.inwaspain.com/

8. Original Nordic Walking Federation. Original pole length recommendation [Internet]. Original Nordic Walking Federation. 2018. Disponible en: https://onwf.org/original-pole-length-recommendation-for-nordic-walking/

9. World Record-Nordic Walking. ONW AND INWA TECHNIQUES [Internet]. World Record-Nordic Walking. 2018. Disponible en: https://www.wr-nw.com/onw-inwa-techniques.html

10. World Record-Nordic Walking. FITTREK TECHNIQUE [Internet]. World Record-Nordic Walking. 2018. Disponible en: https://www.wr-nw.com/fittrek-technique.html

11. Pellegrini B, Peyré-Tartaruga LA, Zoppirolli C, Bortolan L, Savoldelli A, Minetti AE, et al. Mechanical energy patterns in nordic walking: comparisons with conventional walking. Gait & Posture. 2017;51:234-8.

12. Pellegrini B, Peyré-Tartaruga LA, Zoppirolli C, Bortolan L, Bacchi E, Figard-Fabre H, et al. Exploring Muscle Activation during Nordic Walking: A Comparison between Conventional and Uphill Walking. Carrier D, editor. PLOS ONE. 2015;10(9):e0138906.

13. Shim J, Kwon H, Kim H, Kim B, Jung J. Comparison of the Effects of Walking with and without Nordic Pole on Upper Extremity and Lower Extremity Muscle Activation. Journal of Physical Therapy Science. 2013;25(12):1553-6.

14. Saulicz M, Saulicz E, Myśliwiec A, Wolny T, Linek P, Knapik A, et al. Effect of a 4-week Nordic walking training on the physical fitness and self-assessment of the quality of health of women of the perimenopausal age. Menopausal Review. 2015;2:105-11.

15. Park H-S, Lee S-N, Sung D-H, Choi H-S, Kwon TD, Park GD. The Effect of Power Nordic Walking on Spine Deformation and Visual Analog Pain Scale in Elderly Women with Low Back Pain. Journal of Physical Therapy Science. 2014;26(11):1809-12.

16. Park SD, Yu SH. The effects of Nordic and general walking on depression disorder patients' depression, sleep, and body composition. Journal of Physical Therapy Science. 2015;27(8):2481-5.

17. Vílchez Barrera ME, Calvo-Arencibia A. Evidencia científica de la marcha nórdica en Fisioterapia: revisión bibliográfica. Fisioterapia. 2016;38(5):251-64.

18. Fritz T, Caidahl K, Krook A, Lundström P, Mashili F, Osler M, et al. Effects of Nordic walking on cardiovascular risk factors in overweight individuals with type 2 diabetes, impaired or normal glucose tolerance: Randomized Control Nordic Walking Study. Diabetes/Metabolism Research and Reviews. 2013;29(1):25-32.

19. Venojärvi M, Korkmaz A, Wasenius N, Manderoos S, Heinonen OJ, Lindholm H, et al. 12 Weeks' aerobic and resistance training without dietary intervention did not influence oxidative stress but aerobic training decreased atherogenic index in middle-aged men with impaired glucose regulation. Food and Chemical Toxicology. 2013;61:127-35.

20. Gram B, Christensen R, Christiansen C, Gram J. Effects of Nordic Walking and Exercise in Type 2 Diabetes Mellitus: A Randomized Controlled Trial. Clin J Sport Med. 2010;20(5):7.

21. Fritz T, Caidahl K, Osler M, Östenson CG, Zierath JR, Wändell P. Effects of Nordic walking on health-related quality of life in overweight individuals with Type 2 diabetes mellitus, impaired or normal glucose tolerance: Nordic walking-effects on quality of life in overweight individuals. Diabetic Medicine. 2011;28(11):1362-72.

22. Wiklund P, Alen M, Munukka E, Cheng SM, Yu B, Pekkala S, et al. Metabolic response to 6-week aerobic exercise training and dieting in previously sedentary overweight and obese pre-menopausal women: A randomized trial. Journal of Sport and Health Science. 2014;3(3):217-24.

23. Sentinelli F, La Cava V, Serpe R, Boi A, Incani M, Manconi E, et al. Positive effects of Nordic Walking on anthropometric and metabolic variables in women with type 2 diabetes mellitus. Science & Sports. 2015;30(1):25-32.

24. Hartvigsen J, Morsø L, Bendix T, Manniche C. Supervised and non-supervised Nordic walking in the treatment of chronic low back pain: a single blind randomized clinical trial. BMC Musculoskeletal Disorders [Internet]. 2010 [citado 28 de abril de 2019];11(1). Disponible en: https://bmcmusculoskeletdisord.biomedcentral.com/articles/10.1186/1471-2474-11-30

25. Gerhard B, Manuela P, Helga T, Erwin G. Work-site health promotion of frequent computer users: Comparing selected interventions. Work. 2013;(3):233-41.

26. Mannerkorpi K, Nordeman L, Cider Å, Jonsson G. Does moderate-to-high intensity Nordic walking improve functional capacity and pain in fibromyalgia? A prospective randomized controlled trial. Arthritis Research & Therapy. 2010;12(5):R189.

27. Spafford C, Oakley C, Beard JD. Randomized clinical trial comparing Nordic pole walking and a standard home exercise programme in patients with intermittent claudication: Nordic pole walking *versus* standard home exercise programme in patients with intermittent claudication. British Journal of Surgery. 2014;101(7):760-7.

28. Collins EG, O'Connell S, McBurney C, Jelinek C, Butler J, Reda D, et al. Comparison of Walking With Poles and Traditional Walking for Peripheral Arterial Disease Rehabilitation: Journal of Cardiopulmonary Rehabilitation and Prevention. 2012;32(4):210-8.

29. Keast M-L, Slovinec D'Angelo ME, Nelson CRM, Turcotte SE, McDonnell LA, Nadler RE, et al. Randomized Trial of Nordic Walking in Patients With Moderate to Severe Heart Failure. Canadian Journal of Cardiology. 2013;29(11):1470-6.

30. Homma D, Jigami H, Sato N. Effects of Nordic walking on pelvis motion and muscle activities around the hip joints of adults with hip osteoarthritis. Journal of Physical Therapy Science. 2016;28(4):1213-8.

31. Müllerova H, Agusti A, Erqou S, Mapel DW. Cardiovascular Comorbidity in COPD. Chest. 2013;144(4):1163-78.

32. Cugusi L, Manca A, Yeo TJ, Bassareo PP, Mercuro G, Kaski JC. Nordic walking for individuals with cardiovascular disease: A systematic review and meta-analysis of randomized controlled trials. European Journal of Preventive Cardiology. 2017;24(18):1938-55.

33. Wilk et al. Assessment of the selected physiological effects of Nordic Walking performed as a part of a physical exercise program during the second phase of rehabilitation after a myocardial infarction. Rehabilitacja Medyczna. 2005;(9):20-5.

34. Kocur P, Deskur-Śmielecka E, Wilk M, Dylewicz P. Effects of Nordic Walking training on exercise capacity and fitness in men participating in early, short-term inpatient cardiac rehabilitation after an acute coronary syndrome — a controlled trial. Clinical Rehabilitation. 2009;23(11):995-1004.

35. Shin J-H, Kim C-B, Choi J-D. Effects of trunk rotation induced treadmill gait training on gait of stroke patients: a randomized controlled trial. Journal of Physical Therapy Science. 2015;27(4):1215-7.

36. Kang T-W, Lee J-H, Cynn H-S. Six-Week Nordic Treadmill Training Compared with Treadmill Training on Balance, Gait, and Activities of Daily Living for Stroke Patients: A Randomized Controlled Trial. Journal of Stroke and Cerebrovascular Diseases. 2016;25(4):848-56.

37. Latosik E, Zubrzycki IZ, Ossowski Z, Bojke O, Clarke A, Wiacek M, et al. Physiological Responses Associated with Nordic-Walking Training in Systolic Hypertensive Postmenopausal Women. Journal of Human Kinetics. 2014;43(1):185-90.

38. Lejczak A, Josiak K, Węgrzynowska - Teodorczyk K, Rudzińska E, Jankowska E, Banasiak W, et al. Nordic Walking May Safely Increase the Intensity of Exercise Training in Healthy Subjects and in Patients with Chronic Heart Failure. Advances in Clinical and Experimental Medicine. 2016;25(1):145-9.

39. Beyer SE, Sanghvi MM, Aung N, Hosking A, Cooper JA, Paiva JM, et al. Prospective association between handgrip strength and cardiac structure and function in UK adults. Abete P, editor. PLOS ONE. 2018;13(3):e0193124.

40. Piotrowicz E, Zieliński T, Bodalski R, Rywik T, Dobraszkiewicz-Wasilewska B, Sobieszczańska-Małek M, et al. Home-based telemonitored Nordic walking training is well accepted, safe, effective and has high adherence among heart failure patients, including those with cardiovascular implantable electronic devices: a randomised controlled study. European Journal of Preventive Cardiology. 2015;22(11):1368-77.

41. Bulińska K, Kropielnicka K, Jasiński T, Wojcieszczyk-Latos J, Pilch U, Dąbrowska G, et al. Nordic pole walking improves walking capacity in patients with intermittent claudication: a randomized controlled trial. Disability and Rehabilitation. 2016;38(13):1318-24.

42. Langbein WE, Collins EG, Orebaugh C, Maloney C, Williams KJ, Littooy FN, et al. Increasing exercise tolerance of persons limited by claudication pain using polestriding. Journal of Vascular Surgery. 2002;35(5):887-93.

43. Collins EG, Edwin Langbein W, Orebaugh C, Bammert C, Hanson K, Reda D, et al. PoleStriding Exercise and Vitamin E for Management of Peripheral Vascular Disease: Medicine & Science in Sports & Exercise. 2003;35(3):384-93.

44. Collins EG, Langbein WE, Orebaugh C, Bammert C, Hanson K, Reda D, et al. Cardiovascular Training Effect Associated With Polestriding Exercise in Patients With Peripheral Arterial Disease: The Journal of Cardiovascular Nursing. 2005;20(3):177-85.

45. Collins EG, McBurney C, Butler J, Jelinek C, O'Connell S, Fritschi C, et al. The Effects of Walking or Walking-with-Poles Training on Tissue Oxygenation in Patients with Peripheral Arterial Disease. International Journal of Vascular Medicine. 2012;2012:1-8.

46. Oakley C, Spafford C, Beard JD. A Three Month Home Exercise Programme Augmented with Nordic Poles for Patients with Intermittent Claudication Enhances Quality of Life and Continues to Improve Walking Distance and Compliance After One Year. European Journal of Vascular and Endovascular Surgery. 2017;53(5):704-9.

47. Kropielnicka K, Dziubek W, Bulińska K, Stefańska M, Wojcieszczyk-Latos J, Jasiński R, et al. Influence of the Physical Training on Muscle Function and Walking Distance in Symptomatic Peripheral Arterial Disease in Elderly. BioMed Research International. 2018;2018:1-16.

48. Girold S, Rousseau J, Le Gal M, Coudeyre E, Le Henaff J. Nordic walking versus walking without poles for rehabilitation with cardiovascular disease: Randomized controlled trial. Annals of Physical and Rehabilitation Medicine. 2017;60(4):223-9.

49. Rinaldo N, Bacchi E, Coratella G, Vitali F, Milanese C, Rossi A, et al. Effects of Combined Aerobic-Strength Training vs Fitness Education Program in COPD Patients. International Journal of Sports Medicine. 2017;38(13):1001-8.

50. Casaburi R, ZuWallack R. Pulmonary Rehabilitation for Management of Chronic Obstructive Pulmonary Disease. New England Journal of Medicine. 2009;360(13):1329-35.

51. Ochman M, Maruszewski M, Latos M, Jastrzębski D, Wojarski J, Karolak W, et al. Nordic Walking in Pulmonary Rehabilitation of Patients Referred for Lung Transplantation. Transplantation Proceedings. 2018;50(7):2059-63.

52. Jastrzebski D, Ochman M, Ziora D, Labus L, Kowalski K, Wyrwol J, et al. Pulmonary Rehabilitation in Patients Referred for Lung Transplantation. En: Pokorski M, editor. Respiratory Regulation - Clinical Advances [Internet]. Dordrecht: Springer Netherlands; 2013 [citado 27 de abril de 2019]. p. 19-25. Disponible en: http://www.springerlink.com/index/10.1007/978-94-007-4546-9_3

53. Sociedad Española de Medicos Generales y de familia. GEMA 4.4: Guía española para el manejo del asma.

54. Gastaldi AC, Paredi P, Talwar A, Meah S, Barnes PJ, Usmani OS. Oscillating Positive Expiratory Pressure on Respiratory Resistance in Chronic Obstructive Pulmonary Disease With a Small Amount of Secretion: A Randomized Clinical Trial. Medicine. 2015;94(42):e1845.

55. França-Pinto A, Mendes FAR, de Carvalho-Pinto RM, Agondi RC, Cukier A, Stelmach R, et al. Aerobic training decreases bronchial hyperresponsiveness and systemic inflammation in patients with moderate or severe asthma: a randomised controlled trial. Thorax. 2015;70(8):732-9.

56. Sodhi C, Singh S, Bery A. Assessment of the Quality of Life in Patients with Bronchial Asthma, Before and After Yoga: a Randomised Trial. 2014;13(1):6.

57. Lorenc AB, Wang Y, Madge SL, Hu X, Mian AM, Robinson N. Meditative Movement for Respiratory Function: A Systematic Review. Respiratory Care. 2014;59(3):427-40.

58. Cramer H, Posadzki P, Dobos G, Langhorst J. Yoga for asthma: a systematic review and meta-analysis. Annals of Allergy, Asthma & Immunology. 2014;112(6):503-510.e5.

59. Raghavendra P, Shetty P, Shetty S, Manjunath NK, Saoji AA. Effect of high-frequency yoga breathing on pulmonary functions in patients with asthma. Annals of Allergy, Asthma & Immunology. 2016;117(5):550-1.

60. Meyer A, Günther S, Volmer T, Taube K, Baumann HJ. A 12-month, moderate-intensity exercise training program improves fitness and quality of life in adults with asthma: a controlled trial. BMC Pulmonary Medicine [Internet]. diciembre de 2015 [citado 28 de diciembre de 2018];15(1). Disponible en: http://bmcpulmmed.biomedcentral.com/articles/10.1186/s12890-015-0053-8

61. Bruurs MLJ, van der Giessen LJ, Moed H. The effectiveness of physiotherapy in patients with asthma: A systematic review of the literature. Respiratory Medicine. 2013;107(4):483-94.

62. Eichenberger PA, Diener SN, Kofmehl R, Spengler CM. Effects of Exercise Training on Airway Hyperreactivity in Asthma: A Systematic Review and Meta-Analysis. Sports Medicine. 2013;43(11):1157-70.

63. Wanrooij VH, Willeboordse M, Dompeling E, van de Kant KD. Exercise training in children with asthma: a systematic review. British Journal of Sports Medicine. 2014;48(13):1024-31.

64. Mendes FAR, Gonçalves RC, Nunes MPT, Saraiva-Romanholo BM, Cukier A, Stelmach R, et al. Effects of Aerobic Training on Psychosocial Morbidity and Symptoms in Patients With Asthma. Chest. 2010;138(2):331-7.

65. Basaran S, Guler-Uysal F, Ergen N, Seydaoglu G, Bingol-Karakoç G, Ufuk Altintas D. EFFECTS OF PHYSICAL EXERCISE ON QUALITY OF LIFE, EXERCISE CAPACITY AND PULMONARY FUNCTION IN CHILDREN WITH ASTHMA. Journal of Rehabilitation Medicine. 2006;38(2):130-5.

66. Turner S, Eastwood P, Cook A, Jenkins S. Improvements in Symptoms and Quality of Life following Exercise Training in Older Adults with Moderate/Severe Persistent Asthma. Respiration. 2011;81(4):302-10.

67. Flapper BCT, Duiverman EJ, Gerritsen J, Postema K, van der Schans CP. Happiness to be gained in paediatric asthma care. European Respiratory Journal. 2008;32(6):1555-62.

68. Fanelli A, Cabral ALB, Neder JA, Martins MA, Carvalho CRF. Exercise Training on Disease Control and Quality of Life in Asthmatic Children: Medicine & Science in Sports & Exercise. 2007;39(9):1474-80.

69. Ram, FSF, Robinson SM, Black PN, Picot J. Physical training for asthma. 2005;19;(4):CD001116.

70. Mendes FAR, Almeida FM, Cukier A, Stelmach R, Jacob-Filho W, Martins MA, et al. Effects of Aerobic Training on Airway Inflammation in Asthmatic Patients: Medicine & Science in Sports & Exercise. 2011;43(2):197-203.

71. Shaw BS, Shaw I. Pulmonary Function and Abdominal and Thoracic Kinematic Changes Following Aerobic and Inspiratory Resistive Diaphragmatic Breathing Training in Asthmatics. Lung. 2011;189(2):131-9.

72. Wang J-S, Hung W-P. The effects of a swimming intervention for children with asthma. Respirology. 2009;14(6):838-42.

73. Arbillaga-Etxarri A, Gimeno-Santos E, Barberan-Garcia A, Balcells E, Benet M, Borrell E, et al. Long-term efficacy and effectiveness of a behavioural and community-based exercise intervention (Urban Training) to increase physical activity in patients with COPD: a randomised controlled trial. European Respiratory Journal. 2018;52(4):1800063.

74. Guía de Práctica Clínica para el Diagnóstico y Tratamiento de Pacientes con Enfermedad Pulmonar Obstructiva Crónica (EPOC) - Guía Española de la EPOC (GesEPOC). Archivos de Bronconeumología. 2012;48:2-58.

75. Fletcher GF, Ades PA, Kligfield P, Arena R, Balady GJ, Bittner VA, et al. Exercise Standards for Testing and Training: A Scientific Statement From the American Heart Association. Circulation. 2013;128(8):873-934.

76. ATS Statement: Guidelines for the Six-Minute Walk Test. Am J Respir Crit Care Med. 2002;Vol 166:pp 111-117.

77. García-Río F, Calle M, Burgos F, Casan P, del Campo F, Galdiz JB, et al. Espirometría. Archivos de Bronconeumología. 2013;49(9):388-401.

78. Bohannon RW, Crouch R. Minimal clinically important difference for change in 6-minute walk test distance of adults with pathology: a systematic review: Systematic review of MCID in 6MWT. Journal of Evaluation in Clinical Practice. 2017;23(2):377-81.

79. Coelho CM, Reboredo MM, Valle FM, Malaguti C, Campos LA, Nascimento LM, et al. Effects of an unsupervised pedometer-based physical activity program on daily steps of adults with moderate to severe asthma: a randomized controlled trial. Journal of Sports Sciences. 2018;36(10):1186-93.

80. Kim Y, Park I, Kang M. Convergent validity of the International Physical Activity Questionnaire (IPAQ): meta-analysis. Public Health Nutrition. 2013;16(03):440-52.

81. Brazier JE, Harper R, Jones NM, O'Cathain A, Thomas KJ, Usherwood T, et al. Validating the SF-36 health survey questionnaire: new outcome measure for primary care. BMJ. 1992;305(6846):160-4.

82. Vilagut G, Ferrer M, Rajmil L, Rebollo P, Permanyer-Miralda G, Quintana JM, et al. El Cuestionario de Salud SF-36 español: una década de experiencia y nuevos desarrollos. Gaceta Sanitaria. 2005;19(2):135-50.

83. Wyrwich KW, Tierney WM, Babu AN, Kroenke K, Wolinsky FD. A Comparison of Clinically Important Differences in Health-Related Quality of Life for Patients with Chronic Lung Disease, Asthma, or Heart Disease: Clinically Important Differences on the SF-36. Health Services Research. 2005;40(2):577-92.

84. Juniper EF, Buist AS, Cox FM, Ferrie PJ, King DR. Validation of a Standardized Version of the Asthma Quality of Life Questionnaire. Chest. 1999;115(5):1265-70.

85. Perpiñá M, de Diego A, Compte L, Belloch A, Pascual LM. Calidad de vida en el asma: validación del cuestionario AQLQ para su utilización en población española. Archivos de Bronconeumología. 1995;31(5):211-8.

86. Jones PW. Interpreting thresholds for a clinically significant change in health status in asthma and COPD. European Respiratory Journal. 2002;19(3):398-404.

87. Schatz M, Sorkness CA, Li JT, Marcus P, Murray JJ, Nathan RA, et al. Asthma Control Test: Reliability, validity, and responsiveness in patients not previously followed by asthma specialists. Journal of Allergy and Clinical Immunology. 2006;117(3):549-56.

88. Schatz M, Kosinski M, Yarlas AS, Hanlon J, Watson ME, Jhingran P. The minimally important difference of the Asthma Control Test. Journal of Allergy and Clinical Immunology. 2009;124(4):719-723.e1.

89. Vega JM, Badia X, Badiola C, López-Viña A, Olaguíbel JM, Picado C, et al. Validation of the Spanish Version of the Asthma Control Test (ACT). Journal of Asthma. 2007;44(10):867-72.

90. Plaza V, Fernández-Rodríguez C, Melero C, Cosío BG, Entrenas LM, de Llano LP, et al. Validation of the 'Test of the Adherence to Inhalers' (TAI) for Asthma and COPD Patients. Journal of Aerosol Medicine and Pulmonary Drug Delivery. 2016;29(2):142-52.

91. Kendrick KR, Baxi SC, Smith RM. Usefulness of the modified 0-10 Borg scale in assessing the degree of dyspnea in patients with COPD and asthma. Journal of Emergency Nursing. 2000;26(3):0216-22.

92. Hajiro T, Nishimura K, Tsukino M, Ikeda A, Koyama H, Izumi T. Analysis of Clinical Methods Used to Evaluate Dyspnea in Patients with Chronic Obstructive Pulmonary Disease. 1998;158:5.

93. Mulholland A, Ainsworth A, Pillarisetti N. Tools in Asthma Evaluation and Management: When and How to Use Them? The Indian Journal of Pediatrics. 2018;85(8):651-7.

94. National Institutes of Health, National Heart, Lung and Blood Institute. Global Strategy for Asthma management and prevention. [Internet]. Disponible en: www.ginasthma.org

95. Cuesta-Vargas A, Hilgenkamp T. Reference Values of Grip Strength Measured with a Jamar Dynamometer in 1526 Adults with Intellectual Disabilities and Compared to Adults without Intellectual Disability. Gallup AC, editor. PLOS ONE. 2015;10(6):e0129585.

96. Stark T, Walker B, Phillips JK, Fejer R, Beck R. Hand-held Dynamometry Correlation With the Gold Standard Isokinetic Dynamometry: A Systematic Review. PM&R. 2011;3(5):472-9.

97. Norman K, Stobäus N, Gonzalez MC, Schulzke J-D, Pirlich M. Hand grip strength: Outcome predictor and marker of nutritional status. Clinical Nutrition. 2011;30(2):135-42.

98. IPAQ. Guidelines for Data Processing and Analysis of the International Physical Activity Questionnaire (IPAQ) – Short and Long Forms [Internet]. 2005. Disponible en: http://www.academia.edu/5346814/Guidelines_for_Data_Processing_and_Analysis_of_t he_International_Physical_Activity_Questionnaire_IPAQ_Short_and_Long_Forms_Co nte nts.

99. Miller MR. Standardisation of spirometry. European Respiratory Journal. 2005;26(2):319-38.

100. Kimura Y, Takahashi M, Wada F, Hachisuka K. Differences in the Peak Cough Flow among Stroke Patients With and Without Dysphagia. Journal of UOEH. 2013;35(1):9-16.

101. Calaf N. Medición de las presiones respiratorias máximas. En: Comité científico SEPAR. Manual SEPAR de procedimientos: procedimientos de evaluación de la función pulmonar II [Internet]. Sociedad Española de Neumología y Cirugía Torácica. 2004;122-44.

102. Sergio Sancho Marín. Análisis comparativo de la medición de las presiones respiratorias máximas según dos protocolos distintos. 2018;

103. Morales P, Sanchis J, Cordero PJ, Díez JL. Presiones respiratorias estáticas máximas en adultos. Valores de referencia de la población caucasica mediterránea. Archivos de Bronconeumología. 1997;(33):213-9.

104. Barreiro E, Bustamante V, Cejudo P, Gáldiz JB, de Lucas P, et al. Normativa SEPAR sobre disfunción muscular de los pacientes con Enfermedad Pulmonar Obstructiva Crónica. Archivos de Bronconeumología. 51(8).

105. Mathiowetz V et al. Grip and pinch strength: normative data for adults. Arch Phys Med Rehabil. 1985;66(2):69-74.

106. Nici L, Donner C, Wouters E, Zuwallack R, Ambrosino N, Bourbeau J, et al. American Thoracic Society/European Respiratory Society Statement on Pulmonary Rehabilitation. American Journal of Respiratory and Critical Care Medicine. 2006;173(12):1390-413.

107. Garber CE, Blissmer B, Deschenes MR, Franklin BA, Lamonte MJ, Lee I-M, et al. Quantity and Quality of Exercise for Developing and Maintaining Cardiorespiratory, Musculoskeletal, and Neuromotor Fitness in Apparently Healthy Adults: Guidance for Prescribing Exercise. Medicine & Science in Sports & Exercise. 2011;43(7):1334-59.

108. Lincoln Y, Guba E. Naturalistic inquiry. Beverly Hills, CA:Sega.

109. McHugh. Interrater reliability: the kappa statistic. Biochem Med. 2012;22(3):276-82.

12. Appendix

Appendix 1: Contraindications for participation in the study.

12.2.1 Contraindicaciones para la realización de la prueba de 6MWT.

Following ATS/ERS (2).

Absolutes:

- Unstable angina in the last month.
- Acute myocardial infarction in the last month.

Relatives:

- Resting heart rate greater than 120.
- Systolic blood pressure greater than 180mmHg.
- Diastolic blood pressure greater than 100mmHg.

12.2.2 Contraindications for performing a spirometry.

Following SEPAR (3).

Absolutes:

- Hemodynamic instability.
- Pulmonary embolism (until adequate anticoagulation).
- Recent pneumothorax (2 weeks after reexpansion).
- Acute hemoptysis.
- Active respiratory infections (tuberculosis, norovirus, influenza).
- Recent myocardial infarction (7 days).
- Unstable angina.
- Enlarged or large thoracic aortic aneurysm (>6 cm).
- Intracranial hypertension.
- Acute retinal detachment.

Relatives:

- Children under 5-6 years old.
- Confused or demented patients.
- Recent abdominal or thoracic surgery.
- Recent brain, eye, or ear, nose, and throat surgery.

- Diarrhea or acute vomiting, nausea.
- Hypertensive crisis.
- Oral or facial problems that prevent or make it difficult to place and hold the mouthpiece.

12.2.3 Classification of asthma exacerbations

	Mild crisis	Moderate-severe crisis	Respiratory arrest
Dyspnea	Mild	Moderate-intense	Very intense
Speech	Paragraphs	Phrases-words	
Respiratory rate (x')	Augmented	>20-30	
Heart rate (x)	<100	>100-120	Bradycardia
Accessory muscle use	Absent	Present	Thoracoabdominal paradoxical movement
wheezing	Presents	presents	auscultatory silence
Conscience level	Normal	Normal	Diminished
paradoxical pulse	Absent	>12-25mmHg	Absence (muscle fatigue)
FEV1 or PEF (reference values)	>70%	<70%	
SaO2 (%)	>95%	90-95%	<90%
PaO2mmHg	Normal	80-60	<60
PaCO2	<40	>40	>40
FEV: forced expiratory volume in the first second; PEF: peak expiratory flow; x': per minute; SaO2: oxyhemoglobin saturation; PaO2: arterial oxygen pressure; PaCO2: arterial carbon dioxide pressure.			

(4)

1. ATS Statement: Guidelines for the Six-Minute Walk Test. Am J Respir Crit Care Med. 2002;Vol 166:pp 111–117.

2. García-Río F, Calle M, Burgos F, Casan P, del Campo F, Galdiz JB, et al. Espirometría. Archivos de Bronconeumología. 2013;49(9):388-401.

3. Sociedad Española de Médicos Generales y de familia. GEMA 4.4: Guía española para el manejo del asma.

Appendix 2. Patient's Diary

**Day: ___/___/_____**			
**Number of steps**			
		Yes	No
**Síntomas**	Dyspnea (feeling of respiratory suffocation)		
	From 0 to 10 (Borg scale):		
	Wheezing		
	Expectoration		
	Cough		
		Yes	No
PEF (L/min)			
Medication taking			
Which?			
Emergency visit			

I would say that today I feel: ☹ 😐 ☺

The word that best defines my mood today is:

____

Activities I have done today:

Observations:

If you have any questions, you can contact the research team by writing us an SMS or Whatsapp, calling or sending us an email through the following means: Phone Number : +34 628 10 10 24 e-mail: mn.udc2019@gmail.com

Appendix 3. Patient Information Sheet

STUDY TITLE: COMPARISON OF A NORDIC WALKING PROGRAM VERSUS THE EXCLUSIVE USE OF AN EDUCATIONAL PLAN IN PATIENTS WITH ASTHMA.

RESEARCHER: MARIA VILANOVA PEREIRA

CENTRE: UNIVERSITY OF A CORUÑA

This document is intended to provide you with information about a research study in which you are being invited to participate. This study was approved by the Clinical Research Ethics Committee of Galicia.

If you decide to participate in it, you should receive personalized information from the researcher, read this document first and ask all the questions you need to understand the details about it. If you wish, you can take the document with you, consult it with other people and take the necessary time to decide whether or not to participate..

Participation in this study is completely voluntary. You can decide not to participate or, if you agree to do so, change your mind by withdrawing your consent at any time without giving reasons. We assure you that this decision will not affect the relationship with the health professionals who treat you or the health care to which you are entitled..

What is the purpose of the study?

Check if a training based on Nordic walking has benefits in patients with asthma, with respect to an educational plan.

Why do they offer me to participate?

You are invited to participate because you appear in the allergology at the University Hospital Complex of A Coruña and the HM Modelo Hospital in A Coruña, and you also meet the inclusion criteria for this study: you are over 18 years of age and have been diagnosed with asthma. In addition, you want to participate in this study and have the ability to sign the informed consent. This is why your specialist doctor proposes you in consultation to be part of this project.

What does my participation consist of?

Depending on the group you belong to, you will have to carry out Nordic walking training and/or follow a series of recommendations, and fill in a diary that serves as a record sheet, as well as undergo a series of simple and non-invasive tests.

First, you will undergo a series of measurements, which we will perform again at the end of the treatment period and 3 and 6 months after completion. These measurements will be carried out at the Faculty of Physiotherapy of the Universidade da Coruña and will last approximately 2 hours and a half. They include:

- 6-minute walking test.
- Questionnaire on the level of daily physical activity: International Physical Activity Questionnaire (IPAQ).
- General SF-36 quality of life questionnaire.
- Quality of life questionnaire in people diagnosed with respiratory diseases: Saint George's Respiratory Questionnaire.
- Quality of life questionnaire for people with asthma: Asthma Quality of Life Questionnaire.
- Questionnaire on asthma control and symptoms: Asthma Control Test.
- Measurement of dyspnea according to the Borg Scale and the modified scale of the Medical Research Council.
- Forced spirometry.
- Measurement of the resistance force of the muscles, carried out with a dynamometer, assessing the grip strength.
- In addition to this, we will ask you to keep a daily diary that will provide you with the following data:
  - Number of steps taken each day. This information will be obtained from a mobile application in which you will be instructed.
  - Peak expiratory flow. You will take a daily measurement with a portable meter that will be provided to you.
- Taking medications.
- Emergency department visits.
- Adherence

The program includes an educational plan, which consists of 2 classes of 2 hours each in which you will be provided with information about your disease and recommendations and advice on your care. These classes will take place at the same test site, the Faculty of Physiotherapy of the Universidade da Coruña.

If you belong to the group that will carry out a Nordic walking training, you will be subjected to a training of 60 minutes a day, 3 days a week for 8 weeks. The training place will be urban,

that is, in surroundings of the city of A Coruña. This will include joint mobility exercises, stretching and relaxation, as well as a 30-minute walk doing Nordic walking.

Therefore, your participation will have a total estimated duration of 10 weeks, and 3 subsequent follow-up visits.

The Nordic walking group, once the intervention is over, will also be summoned for a group interview, of the focus group type, in which they will talk with their group companions about different aspects raised by the moderator of the meeting, in in relation to their experience and satisfaction with the proposed Nordic walking activity. During this group interview, you will have total freedom when giving your answers, and your interaction with your classmates will be necessary. You will be recorded on video and audio, and your answers will be transcribed for later analysis. Only the researchers of this project will have access to this recording and transcript, and they will not be disseminated in any case. They will be stored in an encrypted folder, on a computer with a password that will remain at the Faculty of Physiotherapy of the University of La Coruña, until the data analysis is complete. In this same document, in the section "Information regarding your data", you have information on how to contact the data protection delegate of the University of La Coruña, in case you want to make any query, claim or exercise rights in this regard. of this material.

Para la realización To carry out this study, given the measurements that will be made, you must have a mobile device with an Internet connection, in which we will ask you to download an application in charge of counting the steps you take in your day to day. You will be instructed on how to download the application, its basic operation and what data we are interested in from it.

What inconveniences does my participation have?

Your participation does not imply additional inconvenience to those of performing moderate physical exercise.

Note that we are going to use submaximal type stress tests (6-minute walk test) as an evaluation tool, which does not entail any risk for you, since they are tests in which the maximum of your capacities is not reached (a wide margin to the limit of them), are limited by their own symptoms and sensations, and their vital signs (heart rate and oxygen saturation) will be continuously monitored. They are no more dangerous than general sports practice, and their duration will not exceed 10-15 minutes. It is true that as much effort and physical activity as they are, you may feel tired after doing them.

Both the main researcher of the project, Ms. María Vilanova Pereira (Col. 3524) and the secondary researcher, Dr. Ana Lista Paz (Col. 2122), and the person who performs the evaluations, together with Dr. Lista, the collaborating researcher Margarita Barral Fernández (Col. 3547), are trained in the data collection and training techniques used, and also have civil liability insurance provided by the Official College of Physiotherapists of Galicia, which although as we have said, the risk presented is the same as that of any physical or sports activity, it covers the damages of any incident, inconvenience or accident that may arise, also to third parties, due to the development of their profession in the research field.

If needed, you can take your asthma reliever medication as usual.

Will I get some benefit for participating?

You are not expected to benefit directly from participating in the study. The research aims to discover unknown or unclear aspects of Nordic walking in patients with asthma.

Will I receive the information obtained from the study?

If you wish, you will be provided with a summary of the study results.

Will the results of this study be published?

The results of this study will be sent to scientific publications for dissemination, but no data will be transmitted that allows the identification of the participants.

Information regarding your data:

The collection, treatment, conservation, communication and transfer of your data will be done in accordance with the provisions of the General Data Protection Regulation (EU Regulation 2016-679 of the European Parliament and of the Council, of April 27, 2016) and the regulations Spanish law on personal data protection in force.

The data necessary to carry out this study will be collected and kept in such a way:

- **-** Pseudonymized (Coded), pseudonymization is the processing of personal data in such a way that they cannot be attributed to a data subject without additional information being used. In this study, only the research team will know the code that will allow their identity to be known**.**

The regulations that regulate the processing of personal data grant you the right to access your data, oppose, correct, cancel, limit its processing, restrict or request its deletion. You can also request a copy of these or that it be sent to a third party (portability right).

To exercise these rights, you can contact the Center's Data Protection Officer, via email dpd@udc.gal. The person in charge of data protection at the center is Luz María Puente Aba, postal address Rúa da Maestranza,9, 15001, A Coruña. The contact telephone number is 881 011 605, or 88 101 161.

Likewise, you have the right to file a claim with the Spanish Data Protection Agency when you consider that any of your rights has not been respected..

Only the research team and the health authorities, who have a duty to maintain confidentiality, will have access to all the data collected by the study. Information that cannot be identified may be transmitted to third parties.

10 years after the end of the study, the data collected will be deleted or kept anonymous for use in future research according to what you choose in the consent signature sheet.

Are there financial interests in this study?

The researcher will not receive specific remuneration for dedication to the study.

You will not be compensated for participating. The results of the study may lead to commercial products or patents; in this case, you will not participate in the economic benefits generated.

How to contact the research team of this study?

You can contact María Vilanova Pereira, on the phone +34 628101024 or at the email marchanordicafisio@gmail.com, or with your reference doctor, collaborating researcher of this study, at the numbers detailed previously in this document (paragraph of information regarding your data)

Thank you very much for your help

Appendix 4. Informed consent

INFORMED CONSENT DOCUMENT FOR PARTICIPATION IN A RESEARCH STUDY

Title: Comparison of a Nordic walking program versus the exclusive use of an education plan in patients with asthma.

Mr./Miss ____

________, adult, with ID __________ and residence in ______________________ ________________________________,

I DECLARE that

I have been informed of the characteristics of the study	Yes ☐ No ☐
I have read the information sheet given to me	Yes ☐ No ☐
I was able to make the observations or questions and my doubts were clarified	Yes ☐ No ☐
I have understood the explanations that have been provided to me and how you sewed my participation in the study	Yes ☐ No ☐
I know how and to whom I have to contact to ask questions about the study in the present and in the future	Yes ☐ No ☐
I have been informed of the risks associated with my participation	Yes ☐ No ☐
I do not meet any of the exclusion criteria as a participant and I know that if this changes at any time I must let the research team know	Yes ☐ No ☐
I confirm that my participation is voluntary	Yes ☐ No ☐
I understand that I can revoke the consent at any time without having to give explanations and without this negatively affecting me.	Yes ☐ No ☐

I CONSENT

Participate in study	Yes ☐ No ☐
That the data provided is used for research	Yes ☐ No ☐
That the data provided in scientific publications be used	Yes ☐ No ☐
That the data provided in meetings and congresses be used	Yes ☐ No ☐
That the data provided is used for teaching	Yes ☐ No ☐
That it be recorded in audio to obtain the data	Yes ☐ No ☐
That it be recorded on video to obtain the data	Yes ☐ No ☐
Verbatim quotes from my interventions, without being identified, are used in publications	Yes ☐ No ☐
That the data be kept anonymously at the end of the study for use in future research	Yes ☐ No ☐
That the coded data be kept at the end of the study for use in future research as long as they guarantee the treatment of the data in accordance with this consent	Yes ☐ No ☐
Contact me for new information	Yes ☐ No ☐

I request

Access the general results of the study	Yes ☐ No ☐

Access information about my derivative of the study	Yes ☐ No ☐
Access scientific articles once they were published	Yes ☐ No ☐
The destruction of my data once the study is finished	Yes ☐ No ☐
Include the following restrictions on the use of my data:	

And in proof of conformity, I sign this document in the place and on the date indicated below.

____________, _______ of _____________________ of ___ ___.

Name and surname of the participant:	*Name and surname of the researcher:*
Signature:	Signature:

Appendix 5. Patient data collection notebook

Sociodemographic data

Gender: Male Female

Month and year of birth: ____/________

Age: ___

Nacionality: ____

Currently work status:

- ◻ Active
- ◻ Inactive
- ◻ Sick leave
- ◻ Pensionist
- ◻ Other (specify): ____

In case of being inactive/unemployed/sick or retired, for how long?:

Years: ___ Months:____

Profession:____

Toxic habits (smoking)

- Have you ever smoked?
  - ◻ Yes, daily *(exclusion)*
  - ◻ Yes, occasionally *(exclusion).*
  - ◻ I do not currently smoke, but have smoked before.
  - ◻ No, I have never smoked.

For people who do not currently smoke, but have smoked in the past (former smokers)

- At what age did you start smoking? ____ years.
- How long have you stopped smoking? ____years and months.

◻ Therefore, the total time he has been a smoker has been: ____years and ____months.

What type and amount of tobacco did you smoke on average per day?

Number of cigarettes/day: ____

Number of pipes/day: ___ (1 pipe/week= 0.14 pipe/day).

- Are you currently exposed to smoke? (passive smoker):

  Yes

  For how long? (hours/day): ____

  No

- Calculation of the smoking index (years smoked x cigarettes smoked/20)/20)

Respiratory history.

- Do you suffer or have you suffered from any of the following respiratory diseases?

	YES	NO
COPD		
Chronic allergy		
Guy:		
Cystic fibrosis		
Pulmonary fibrosis		
bronchiectasis		
Tuberculosis		
Pleural effusion		
When?		
Pneumothorax		
When?:		
Others:		

- Have you undergone a lung transplant or lung resection?
  - □ Yes.
  - □ No.
- Do you frequently suffer from pneumonia/respiratory infections?
  - □ Yes.
    - How often? (number times/year):_____
    - When was the last one? (in months):_____
  - □ No.

Pathological history.

- Do you have or have you ever had any of the following diseases?

	SÍ	NO
High blood pressure		
It is controlled?		
Acute myocardial infarction		
When? _____ years _____ months		
Angina pectoris		
Heart transplant		
When? _____ years _____ months		
Other cardiovascular diseases:		
Diabetes		
Varicose veins in the legs		
Osteoarthritis, arthritis or rheumatism		
Urinary/faecal incontinence		
Malignant tumors		
Thoracic/abdominal surgery		
When?		
Reason?		
Neuromuscular diseases		
Chronic back pain (lumbar)		
Chronic back (cervical) pain		
Stomach or duodenal ulcer		
High cholesterol		
Waterfalls		
Chronic skin problems		
Chronic constipation		
Cirrhosis, liver dysfunction		
Depression		
Chronic anxiety		
Other mental problems		
Stroke (embolism, cerebral infarction, cerebral hemorrhage)		
Migraine or frequent headache		
Hemorrhoids		
Osteoporosis		
Thyroid problems		
Kidney problems		
Prostate problems (men only)		
Menopausal period problems (women only)		
Permanent injuries or dysfunctions caused by an accident		
None of the above		
Any illness or musculoskeletal disorder that prevents or makes it difficult for you to walk?		
Other underlying diseases.		

PHARMACOLOGICAL CONSUMPTION

What medications do you currently take?

□ Medicines for cold, flu, throat

□ Medicines for the bronchial tubes (inhalers)

Which? ____

- □ Pain medicine
- □ Antibiotics
- □ Tranquilizers, relaxants (Exclusion if they are muscle relaxants and you take them regularly), sleeping pills
- □ Antidepressants (Exclusion if they are barbiturates)
- □ Allergy medications
- □ Medicines for rheumatism
- □ Heart medicine
- □ Blood pressure medications
- □ Medications for stomach and/or digestive disorders
- □ Pills to prevent pregnancy (for women only)
- □ Hormones for menopause (for women only)
- □ Medications to lose weight
- □ Cholesterol-lowering medications
- □ Diabetes medications
- □ Thyroid medications
- □ None of the above
- □ Other:____

PHYSICAL EXPLORATION

**ANTHROPOMETRIC MEASURES**		
Weight (kg)	Height (cm)	BMI (kg/m^2)
**VITAL SIGNS**		

HR (lpm):
SpO_2(%):

DYNAMOMETRY

Left hand		Right hand	
1º		1º	
2º		2º	
3º		3º	

SPIROMETRY

Parameter	Obtained value	Reference value
FVC		
FEV1		
FVC/FEV1		
FEF25-75%		

RESPIRATORY MUSCULATURE STRENGTH

Parameter	Obtained value (cmH20)	Reference value
MIP		
MEP		

Observations

____

____

____

DISCONTINUATION IN THE STUDY

Complete if during the interview, physical examination or during the tests, it is detected that the subject does not meet any of the exclusion criteria, or if the patient decides that he does not want to continue with the program in the course of it.

Reason for exclusion:

____

____

____

Appendix 6. Record of the 6-minute walking test

Subject:

Stretch	Distance traveled (m)	Time (s)	HR (lpm)
1	30		
2	60		
3	90		
4	120		
5	150		
6	180		
7	210		
8	240		
9	270		
10	300		
11	330		
12	360		
13	390		
14	420		
15	450		
16	480		
17	510		
18	540		
19	570		
20	600		
21	630		
22	660		
23	690		
24	720		
Additional distance			m
**Total distance**			m
Number of stops			-
Total time of stops			s

Stops	Time (s)	Reason
1'	You're doing great, 5 minutes to go	
2'	Perfect, continue like this, 4 minutes left	
3'	You are in the middle of the test, you are doing very well	
4'	Perfect, continue like this, 2 minutes left	
5'	You are doing very well, 1 minute left	
5'45"	You must stop with the indication: STOP	
6'	STOP	

Date and Time:

**BASELINE VALUES**		
HR		Lpm
SpO_2		%
RR		rpm
Dispnea		Borg
Lower limb fatigue		Borg
**FINAL VALUES**		
HR		lpm
SpO_2		%
RR		Rpm
Dispnea		Borg
Lower limb fatigue		Borg
**Recuperation values**		
Time	HR (lpm)	SpO2 (%)
1'		
2'		
3'		
4'		
5'		
**REASONS TO SUSPEND THE 6MWT**		
	Chest pain	
	Intolerable dyspnea	
	Cramps in lower limbs	
	Unexplained diaphoresis	
	Paleness, feeling dizzy	
	SpO2%<85%	
	Want to stop	
	Chest pain	
CONTRAINDICATIONS FOR THE 6MWT		
	Unstable angina (<1 month)	
	AMI (<1 month)	
	HR at rest >120 bpm	
	HR at rest <40 bpm + SBP <110mmHg	
	Uncontrolled hypertension or TAS >180mmHg at rest	
	DBP > 100mmHg at rest (when there are symptoms of hypertension)	

Appendix 7. International Physical Activity Questionnaire. Short version.

We are interested in knowing about the kind of physical activity that people do as part of their daily lives. The questions will ask about the time you spent being physically active in the last 7 days. Please answer each question even if you do not consider yourself to be an active person.

Please think about those activities that you do as part of work, in the garden and at home, to go from one place to another, and in your free time for rest, exercise or sports.

Think about all the vigorous activities you did in the last 7 days. Vigorous activities are those that require strong physical exertion and make you breathe much harder than normal. Think only of those activities that you did for at least 10 minutes continuously.

1. During the last 7 days, on how many days did you do vigorous physical activity such as lifting heavy objects, digging, aerobics, or fast cycling?

_____ days per week

2. How much total time did you spend doing vigorous physical activity on one of those days?

_____ horas por día

_____ minutos por día

Think about all the moderate activities you did in the last 7 days. Moderate activities are those that require moderate physical exertion and make you breathe a little harder than normal. Think only of those activities that you did for at least 10 minutes at a time.

3. During the last 7 days, on how many days did you do moderate physical activities such as carrying light objects, cycling at a regular pace, or playing doubles tennis? Do not include walks.

_____ days per week

4. How much time do you usually spend on one of those days doing moderate physical activity?

_____ **hours per day**

_____ **minutes per day**

Think about the time you spent walking in the last 7 days. This includes housework, walking from place to place, or any other walking that you did solely for recreation, sport, exercise, or pleasure..

5. During the last 7 days, on how many days did you walk for at least 10 minutes at a time?

_____ **Days per week**

→

6. How much time did you usually spend on one of those days walking?

_____ **Hours per day**

_____ **Minutes per day**

The last question refers to the time you spent sitting in the week in the last 7 days. Include time sitting at work, home, studying, and in your free time. This may include time sitting at a desk, visiting friends, reading, or sitting or lying down watching TV.

7. During the last 7 days, how much time did you spend sitting on one day weekday?

_____ **hours per day**

_____ **minutes per day**

Anexo 8. Cuestionario de calidad de vida *Short Form- Health Survey* 36

SF-36 QUESTIONNAIRE

Name:____________________ **Ref. Dr:**__________________ **Date:** _______

ID#: _______________ **Age:** _______ **Gender: M / F**

Please answer the 36 questions of the **Health Survey** completely, honestly, and without interruptions.

GENERAL HEALTH:
In general, would you say your health is:
Excellent   Very Good   Good   Fair   Poor

Compared to one year ago, how would you rate your health in general now?
- Much better now than one year ago
- Somewhat better now than one year ago
- About the same
- Somewhat worse now than one year ago
- Much worse than one year ago

LIMITATIONS OF ACTIVITIES:
The following items are about activities you might do during a typical day. Does your health now limit you in these activities? If so, how much?

Vigorous activities, such as running, lifting heavy objects, participating in strenuous sports.
Yes, Limited a lot   Yes, Limited a Little   No, Not Limited at all

Moderate activities, such as moving a table, pushing a vacuum cleaner, bowling, or playing golf
Yes, Limited a Lot   Yes, Limited a Little   No, Not Limited at all

Lifting or carrying groceries
Yes, Limited a Lot   Yes, Limited a Little   No, Not Limited at all

Climbing several flights of stairs
Yes, Limited a Lot   Yes, Limited a Little   No, Not Limited at all

Climbing one flight of stairs
Yes, Limited a Lot   Yes, Limited a Little   No, Not Limited at all

Bending, kneeling, or stooping
Yes, Limited a Lot   Yes, Limited a Little   No, Not Limited at all

Walking more than a mile
Yes, Limited a Lot   Yes, Limited a Little   No, Not Limited at all

Walking several blocks
Yes, Limited a Lot   Yes, Limited a Little   No, Not Limited at all

Walking one block
Yes, Limited a Lot   Yes, Limited a Little   No, Not Limited at all

Bathing or dressing yourself
◯Yes, Limited a Lot ◯Yes, Limited a Little ◯No, Not Limited at all

PHYSICAL HEALTH PROBLEMS:
During the past 4 weeks, have you had any of the following problems with your work or other regular daily activities as a result of your physical health?

Cut down the amount of time you spent on work or other activities
◯Yes ◯No

Accomplished less than you would like
◯Yes ◯No

Were limited in the kind of work or other activities
◯Yes ◯No

Had difficulty performing the work or other activities (for example, it took extra effort)
◯Yes ◯No

EMOTIONAL HEALTH PROBLEMS:
During the past 4 weeks, have you had any of the following problems with your work or other regular daily activities as a result of any emotional problems (such as feeling depressed or anxious)?

Cut down the amount of time you spent on work or other activities
◯Yes ◯No

Accomplished less than you would like
◯Yes ◯No

Didn't do work or other activities as carefully as usual
◯Yes ◯No

SOCIAL ACTIVITIES:
Emotional problems interfered with your normal social activities with family, friends, neighbors, or groups?

◯Not at all ◯Slightly ◯Moderately ◯Severe ◯Very Severe

PAIN:
How much bodily pain have you had during the past 4 weeks?

◯None ◯Very Mild ◯Mild ◯Moderate ◯Severe ◯Very Severe

During the past 4 weeks, how much did pain interfere with your normal work (including both work outside the home and housework)?

◯Not at all ◯A little bit ◯Moderately ◯Quite a bit ◯Extremely

ENERGY AND EMOTIONS:
These questions are about how you feel and how things have been with you during the last 4 weeks. For each question, please give the answer that comes closest to the way you have been feeling.

Did you feel full of pep?
- All of the time
- Most of the time
- A good Bit of the Time
- Some of the time
- A little bit of the time
- None of the Time

Have you been a very nervous person?
- All of the time
- Most of the time
- A good Bit of the Time
- Some of the time
- A little bit of the time
- None of the Time

Have you felt so down in the dumps that nothing could cheer you up?
- All of the time
- Most of the time
- A good Bit of the Time
- Some of the time
- A little bit of the time
- None of the Time

Have you felt calm and peaceful?
- All of the time
- Most of the time
- A good Bit of the Time
- Some of the time
- A little bit of the time
- None of the Time

Did you have a lot of energy?
- All of the time
- Most of the time
- A good Bit of the Time
- Some of the time
- A little bit of the time
- None of the Time

Have you felt downhearted and blue?
- All of the time
- Most of the time
- A good Bit of the Time
- Some of the time
- A little bit of the time
- None of the Time

Did you feel worn out?
- All of the time
- Most of the time
- A good Bit of the Time
- Some of the time
- A little bit of the time
- None of the Time

Have you been a happy person?
- All of the time
- Most of the time
- A good Bit of the Time
- Some of the time
- A little bit of the time
- None of the Time

Did you feel tired?
- All of the time
- Most of the time
- A good Bit of the Time
- Some of the time
- A little bit of the time
- None of the Time

SOCIAL ACTIVITIES:
During the past 4 weeks, how much of the time has your physical health or emotional problems interfered with your social activities (like visiting with friends, relatives, etc.)?

- All of the time
- Most of the time
- Some of the time
- A little bit of the time
- None of the Time

GENERAL HEALTH:
How true or false is each of the following statements for you?

I seem to get sick a little easier than other people
Definitely true Mostly true Don't know Mostly false Definitely false

I am as healthy as anybody I know
Definitely true Mostly true Don't know Mostly false Definitely false

I expect my health to get worse
Definitely true Mostly true Don't know Mostly false Definitely false

My health is excellent
Definitely true Mostly true Don't know Mostly false Definitely false

Appendix 9. *Asthma Quality of Life Questionnaire*

Please answer all questions by circling the answer that best describes how you have felt over the past two weeks due to asthma.

In general, how often during the last 2 weeks did you:

	Always	Almost always	A lot of time	Part of time	Few time	Never
1... feel short of breath due to asthma?						
2 ... felt that the dust bothered her, or had to avoid a place because of the dust?						
3 ... felt frustrated or irritated because of his asthma?						
4... felt discomfort due to cough?						
5... was afraid of not having his asthma medication on hand?						
6... noticed a feeling of choking or tightness in the chest?						
7... felt that tobacco smoke bothered him, or he had to avoid a place because of tobacco smoke?						
8... had difficulty sleeping well at night due to asthma?						
9... had asthma symptoms from exposure to tobacco smoke?						
10... felt wheezing or whistling in his chest?						
11... felt that it bothered you or had to avoid leaving the house due to weather or air pollution?						
12...feel intense efforts (such as hurrying, exercising, running up stairs, playing sports)						
13... feel moderate exertion (such as walking, housework, gardening, grocery shopping, climbing stairs without running)						

To what extent has your asthma limited you from doing these activities during the past 2 weeks?

*Lim: limited	Tottally lim	Extremly im	Very lim	Moderat ed lim	Sort lim	Little lim	No lim at [illegible]
14...social activities (such as talking, playing with children/pets, visiting friends/family)							
15...activities related to your job (tasks you have to do at your job*)							

Anexo 10. *Asthma Control Test*

Este cuestionario consta de 5 preguntas, cuyas respuestas se puntuan de 0 a 6. Dichas puntuaciones se suman y el resultado se divide entre 5. Si el resultado es:

- ≤0.75: control adecuado del asma.
- 0.75-1.5 Asma parcialmente controlada.
- >1.50: control inadecuado del asma.

1. In the past 4 weeks, for how long has asthma prevented you from completing your usual activities at work, school, or home?

   □ Always
   □ Almost always
   □ Sometimes
   □ Rarely
   □ Never

2. During the past 4 weeks, how often have you felt short of breath?

   □ More than once a day
   □ Once a day
   □ 3 to 6 times a week
   □ Once or 2 times a week
   □ Never

3. During the past 4 weeks, how often have your asthma symptoms (wheezing/heartburn, cough, shortness of breath, chest tightness, or pain) woke you up at night or earlier than usual in the morning?

   □ 4 or more nights per week
   □ 2 to 3 nights per week
   □ Once a week
   □ Once or twice a week
   □ Never

4. During the last 4 weeks, how often have you used your rescue medication inhaler or nebulizer (eg Salbutamol)?

   □ 3 or more times a day
   □ 1 or 2 times a day
   □ 2 or 3 times a week
   □ Once a week or less
   □ Never

5. To what extent would you say your asthma has been controlled in the last 4 weeks?

- ◻ Nothing controlled
- ◻ Poorly controlled
- ◻ Somewhat controlled
- ◻ Well controlled
- ◻ Fully controlled

Anexo 11. Test de Adhesión a los Inhaladores (TAI)

	Score
**1. How often did you forget to take your regular inhalers in the last 7 days?**<br>☐1. Always ☐2. More than half ☐3. About half ☐4. Less than half ☐5. None	
**2. You forget to take your inhalers:**<br>☐1. Always ☐2. Almost always ☐3. Sometimes ☐4. Almost never ☐5. Never	
**3. When you are feeling well, you stop taking your inhalers:**<br>☐1. Always ☐2. Almost always ☐3. Sometimes ☐4. Almost never ☐5. Never	
**4. At the weekend or when you go on holiday, you stop taking your inhalers:**<br>☐1. Always ☐2. Almost always ☐3. Sometimes ☐4. Almost never ☐5. Never	
**5. When you are anxious or sad, you stop taking your inhalers:**<br>☐1. Always ☐2. Almost always ☐3. Sometimes ☐4. Almost never ☐5. Never	
**6. You stop taking your inhalers out of fear of potential side effects:**<br>☐1. Always ☐2. Almost always ☐3. Sometimes ☐4. Almost never ☐5. Never	
**7. You stop taking your inhalers because you believe that they are of little help in treating your condition:**<br>☐1. Always ☐2. Almost always ☐3. Sometimes ☐4. Almost never ☐5. Never	
**8. You take fewer inhalations than prescribed by your doctor:**<br>☐1. Always ☐2. Almost always ☐3. Sometimes ☐4. Almost never ☐5. Never	
**9. You stop taking your inhalers because you believe that they interfere with your day-to-day or work life:**<br>☐1. Always ☐2. Almost always ☐3. Sometimes ☐4. Almost never ☐5. Never	
**10. You stop taking your inhalers because you have trouble paying for them:**<br>☐1. Always ☐2. Almost always ☐3. Sometimes ☐4. Almost never ☐5. Never	

A healthcare professional responsible for the patient must answer the following two questions according to the data that appears in the patient's medical record (question 11) and after confirming their inhalation technique (question 12).

**11. Does the patient know or remember the regimen (dose and frequency) that they were prescribed?**<br>☐1. No ☐2. Yes	
**12. The patient's inhalation technique for the device:**<br>☐1. Has critical errors ☐2. Has no critical errors or is correct	
**TOTAL SCORE**	

Appendix 12. Modified *Medical Research Council*

GRADE	DYSPNEA
0	Dyspnea only with very intense physical activity.
1	Dyspnea when walking too fast or going up a slight hill.
2	Inability to walk at the same pace as other people of the same age.
3	Dyspnea that forces to stop before 100 meters despite walking at its pace and on flat ground.
4	Dyspnea when making minimum efforts of daily physical activity such as getting dressed or that prevent the patient from leaving his home.

Anexo 13. Borg modified scale

10	Maximum
9	Very, very strong
8	- - - - - - - - - - - - - -
7	Very strong
6	- - - - - - - - - - - - - -
5	Strong
4	Little strong
3	Regular
2	Slight
1	Very slight
0	Nothing

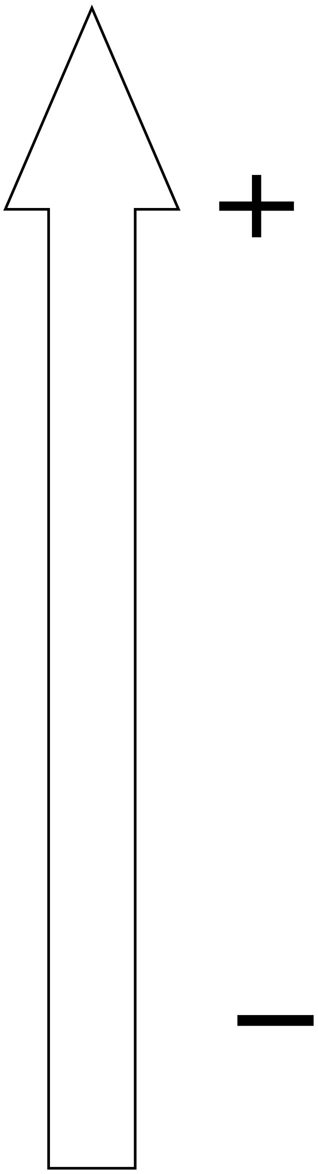

Appendix 14. Educational plan for patients

1. Presentation of the program and patients

The people in charge of the study will explain to the patients how it will be carried out, in terms of intervention, dates and times. In addition, the patients will be able to get to know each other, since part of the total, the study group, will carry out the sessions together.

It is important that the participants are aware of what their pathology entails and the importance of continuous treatment even if they do not have discomfort. It is just as important that they know how to recognize the symptoms of the disease and the signs and symptoms of its aggravation, in order to be able to react to them and prevent a crisis or exacerbation, and that they use inhalers correctly, monitor symptoms and peak expiratory flow (PEF) (1).

2. Asthma information

2.1 What is asthma? Definition and description

Asthma: Heterogeneous disease normally characterized by chronic airway inflammation (2), which produces narrowing of the bronchi. In addition, it is accompanied by hyperreactivity or irritability of these. The bronchi are the tubes that carry oxygen from the air to our lungs. This situation of inflammation and hyperreactivity causes them to narrow or close abruptly in different situations. If the inflammation is perpetuated over time, the disease becomes chronic (3).

Historically, it has been defined with the following respiratory symptoms: wheezing, shortness of breath, chest tightness, cough that varies over time and intensity, along with variable expiratory airflow limitation.

All these symptoms vary in time and intensity, often depending on factors such as: exercise, exposure to allergens or irritants, changes in the weather or viral respiratory infections.

Symptoms and airflow limitation can resolve spontaneously or with pharmacological treatment, however, on other occasions, patients may suffer episodic exacerbations that require them to continue treatment for life (2).

2.2 Asthma prevalence

It affects 300 million people in the world (4). In adults, this prevalence ranges between 3 and 9%, worldwide, and in Spain between 3-4%, being 8% in the population. It is more frequent in developed countries and in recent years its frequency has increased, with theories that

attribute this increase to insufficient exposure to bacteria and infections in children in these countries, which produces a disorder in the maturation of the immune system. It is more common in women than in men, but less common in girls than in boys.

The frequency of the disease means that its socio-health cost for society is very high (3).

2.3 Asthma phenotypes: “types of asthmatics”

- Allergic asthma. The most common. It begins in childhood. Allergic family history is associated such as: eczema, allergic rhinitis, food or drug allergy. Pretreatment sputum from these patients often shows airway inflammation. They normally respond via inhaled corticosteroids.
- Non-allergic asthma. It is not associated with any allergy and there may or may not be evidence of airway inflammation in the sputum. Respond worse to inhaled corticosteroids.
- Late-onset asthma. Usually in women, who suffer the first episode in adulthood. Not associated with allergies and require high doses of inhaled corticosteroids or are relatively resistant to them.
- Asthma with permanent airflow limitation. Patients with long-standing asthma develop permanent limitation as a consequence of airway remodeling.

Asthma and obesity. Obese patients with significant respiratory symptoms and minor airway inflammation (2).

2.4 Asthmatic exacerbation

It is caused by the narrowing of the airway that produces airflow obstruction, which is reversible, the main differentiating characteristic of asthma. This narrowing is produced by bronchial muscle contraction, edema, and mucous hypersecretion.

It can be produced by direct causes, such as respiratory viral infection, tobacco, cold, humidity, allergens and atmospheric pollutants; due to indirect causes, such as physical exercise, food allergens and addictives, pregnancy, storms or thermal inversion, drugs, sinusitis, menstruation or gastroesophageal reflux.

Symptom variability can be assessed with daily PEF measurement.

Exacerbations are acute or subacute episodes characterized by a progressive increase in dyspnea, cough, wheezing, or chest tightness (i.e., any of the typical symptoms), accompanied by decreased expiratory flow (PEF).

Exacerbations can be slow onset, which take days or weeks, and are the most frequent (80% of those who go to the emergency room) and rapid onset, which take less than three hours and have different causes, pathogenesis and prognosis.

Initially, a static evaluation is performed, which allows identifying patients with risk factors, identifying signs and symptoms and objectively measuring the degree of obstruction. After treatment, a second evaluation is carried out, which assesses the changes obtained and the need to perform more diagnostic tests.

The exacerbation is classified as mild, moderate or severe based on the value obtained with the PEF or FEV1.

	PEF or FEV_1
Mild	>70%
moderate	50-70%
Serious	<50%

PEF: forced expiratory peak. FEV_1: fraction of air expelled in the first second.

Treatment consists of reversing the airflow obstruction and hypoxemia, if present. The main thing is to preserve the patient's life, so once this problem is overcome, it will be when the therapeutic plan is reviewed to prevent new crises.

Crises classified as mild can be treated by the patient independently or in a hospital emergency room. Treatment consists of rapid-acting B2 adrenergic agonist bronchodilators, oral glucocorticoids, and oxygen, if necessary. Regarding B2 adrenergic agonist bronchodilators, salbutamol or terbutaline are used, at doses ranging between 200 and 400ug, with an inhalation chamber. 2 to 4 inhalations will be carried out every 20 minutes during the first hour. If the situation does not improve, the patient should be referred to a hospital emergency department, otherwise, inhalation of salbutamol is continued at doses of one to two inhalations every 3-4 hours, until the crisis completely subsides.

The evolution is considered favorable if the symptoms disappear and the PEF is greater than 80% of the reference value or the patient's best value.

In the event that remission of the obstruction is not achieved with this treatment, the patient has already been taking them, has already treated his crisis with other therapeutic options and has failed, or in the event that there is a history of previous exacerbations that required it, the patient should take oral glucocorticoids.

In case of moderate and severe exacerbations, the patient should always go to a medical service (1).

2.5 Asthma management

Asthma control has two parts: symptom control (time without symptoms, reduction or elimination of symptoms with pharmacological treatment) and risk of exacerbations. It is important to note that asthma control is not about how quickly symptoms resolve when medication is taken (2).

2.5.1 Difficult-to-control asthma

It affects 5% of asthmatic patients. Difficult-to-control asthma refers to those patients whose asthmatic disease is characterized by being very aggressive and being poorly or insufficiently controlled (1).

3 Medication

3.1 Types of medications

Control medications.

They are used to reduce airway inflammation, control symptoms, and reduce future risk of exacerbations and decreased lung function.

Rescue medications

They are recommended to prevent short-term exercise-induced bronchoconstriction. One goal should be to reduce or even eliminate the need to resort to this type of medication. This achievement is a measure of success in treating asthma (4).

3.2 Correct administration of inhalers

There are different types of inhalers, depending on the form of presentation of the dose: pressurized inhaler, pressurized inhaler with solution of extrafine particles, powder devices, and nebulizers.

Pressurized inhaler	With spacer chamber
	Without spacer chamber
Pressurized inhaler with extrafine particle solution	
dry powder devices	
Nebulizers	

In addition, pressurized inhalers can vary their form of application, if a spacer chamber is added or not.

It is decided by one or the other depending on the age and the skill of the patient in the use of each one of them. Pressurized cartridges require coordination between inhalation and lung movement, and powder cartridges depend on flow, which must be high (more than 60 l/minute), since particle deposition depends on it.

To solve the problem of lack of coordination between inhalation and lung movement, during the use of pressurized cartridges, a spacer chamber can be used. This also improves the distance and the amount of medicine that reaches the bronchial area. Decreases the deposit in the oropharyngeal region, cough, candidiasis and the risk of systemic deleterious effects.

You have to choose the one that best suits, explain the inhalation pattern, train it, correct it if necessary and review it at each visit.

Nebulizers are used only in special situations (4).

3.2.1 Pressurized cartridge inhaler

Device Maintenance:

- Do not expose to temperatures above 50ºC.
- Keep them protected from direct sunlight.
- Storage at temperatures below 30ºC.
- Keep away from fire. Even though it's empty.
- Perform 2-4 puffs in the air when using it for the first time, as well as if it has been several days since last use.

Device cleaning:

- The cartridge is removed and the casing is washed with water and neutral soap.
- Rinse with plenty of water and dry well (insist on the area where the valve sits).
- Reinsert the cartridge.
- In compact systems, in which the cartridge is not removed, it is preferable to clean the container with a damp or dry cloth, but it is not advisable to disassemble or wet them. (5)

Correct use of the pressurized cartridge inhaler:

1. For pMDI shake before each dose to ensure dose homogeneity.
2. Remove the cap from the pMDI.
3. Air pulsation if it is the first time it is used or it has been 1-2 weeks since its last use.
4. Place the device between the teeth and seal the lips around the mouthpiece.
5. Exhale to near residual volume.
6. Activate the cartridge only once. Begin exhaling afterwards (no more than 3 seconds), slowly to total lung capacity. Slow and deep inspiration: less than 30 litres/minute.
7. Apnea 10 seconds after full inhalation. It increases the permanence of the particles in the lungs, which favors their deposition by sedimentation and diffusion.
8. If two doses are needed, the process is repeated. Pressing twice at the same time reduces the amount of drug that reaches the lungs. (6)

Camera maintenance and cleaning

They are personal cameras, for individual use. It requires cleaning at least once a week. It should be completely disassembled and washed with warm water and a mild detergent. It is allowed to dry without rubbing, in order to avoid the electrostatic charge that could attract the aerosol particles to the walls of the chamber, which would reduce the amount of aerosol that reaches the lungs.

The valves should be checked before using the inhaler, and if they are not in good condition, or if they are cracked, the chamber should be replaced.

3.2.2 Dry powder inhalers

(Dry power inhaler-DPI)

Manteinance

- Store the inhaler in a dry place, protected from moisture.

Cleaning

- Clean the device with a lint-free cloth or dry paper around the mouthpiece after use.
- Then close the inhaler and store it (5).

Correct use of dry powder inhaler

1. Remove the cap from the mouthpiece.
2. Open the DPI, insert the capsule immediately, and close it.
3. Press the button located at the base of the DPI to pierce the capsule.
4. Empty the lungs by avoiding exhalation into the mouthpiece after piercing the capsule.
5. Place the mouthpiece in the mouth. Lips closed around and prevent the tongue from obstructing.
6. Inhale from the beginning with the maximum inspiratory effort, as strong and deep as you can, holding it as long as possible.
7. Stop inhaling only after you have completely filled your lungs.
8. Remove the DPI from the mouth without exhaling into it.
9. Hold your breath for 8-10 seconds when inhalation is complete. (6)

3.2.3 Nebulizers

There are several types of nebulizers: pneumatic or jet, ultrasonic and vibrating mesh. They differ from each other by the way of achieving the aerosol: atomization, piezoelectric crystal that converts electrical energy into ultrasonic waves generated by the aerosol when passing through the solution or electrical energy, respectively..

- They can be used through mouth pieces or masks.
    - Avoid nasal breathing.
    - Avoid aerosol leakage.
    - Avoid spray impaction on the face

- A slow respiratory pattern is recommended, at tidal volume, interspersed with deep inspirations and avoiding hyperventilation.
- The use of a reservoir, of valves that increase the outlet surface during the inspiratory phase, or the fact that nebulization is provided only during the inspiratory phase, reduces aerosol loss, all depending on its design. (5)

4. What can I do?

4.1 Respiratory monitoring

The main thing to change is:

- Number of breaths per minute.

- Depth of respiration (volume of air inspired each time).
- Flow speed.
- Timing (inspiration/expiration)
- Swing.
- Main region in which movement occurs.

There is no evidence that chest movements affect the regional distribution of breathing, but it has been proposed that abnormal proprioceptive afferents associated with upper costal breathing may directly increase the perception of respiratory symptoms (7).

It is initially proposed to control the rhythm of breathing, rather than its volume. For this, it is suggested to lengthen the expiratory times and the pauses at the end of expiration.

In patients with asthma, nasal breathing has been associated with a decrease in nocturnal symptoms (8). In addition, mouth breathing appears associated with moments of crisis (9). Furthermore, it is always preferable to breathe through the nose, since it is a filter, heater and humidifier of the inspired air, which can potentially reduce the impact of asthma (10).

As for pauses, they have been associated with multiple theoretical benefits. For example, 3-second pauses at the end of inspiration (end-inspiratory pauses) are associated with a better distribution of ventilation, allowing air to overcome the secretion barrier (11). It also desensitizes subjects to high concentrations of carbon dioxide and reduces the respiratory rate (12). There are different ways to perform pauses: inspiratory/expiratory, different volumes (maximal inspiration or VR), hold with nose or not, at rest or during activity. The most used: pauses at the end of the expiration, through the nose, until the feeling of discomfort, but not so much as to have to take a deep breath later.

So:

Nasal breaths, 1:3 inspiration:expiration, pausing at the end of expiration until uncomfortable but not so that you need a further deep inspiration, and diaphragmatic, not using the upper costal part

4.2 Lifestyle

- General recommendations:

- Have all mandatory vaccinations up to date.
- Get a flu shot. The flu can contribute to asthma exacerbations, although you have to know that getting vaccinated will not reduce them. Just prevent them from appearing to a greater extent.
- Quit smoking and avoid environments with tobacco smoke (1,2).
- Doing physical activity. Important for general health. Improves cardiopulmonary health (2). Regarding exercise-induced bronchoconstriction:
    - Inhalation of a short-acting beta-agonist (SABA) is recommended 15 minutes before exercise (13).
- Healthy diet, rich in fruit and vegetables.
- Reduce weight in case of overweight or obesity.

4.3 Environment control

- Avoid the use of polluting heating and cooking sources, if unavoidable, in the open air as far as possible.
- Avoid environmental contamination.
- Coping with emotional stress.
- Avoid exercising and spending long periods of time outdoors, under unfavorable weather conditions: very cold temperatures, low humidity or significant air pollution (2).
- In the case of allergic asthma, it is also recommended to reduce exposure to the allergen.

All the actions that we can carry out add up to achieve environmental control and with it the crises, exacerbations and asthmatic symptoms.

If you need an analgesic, paracetamol is recommended, in doses of less than 650mg/dose. Non-steroidal anti-inflammatory drugs are not recommended, as they can cause bronchoconstriction (1)

5. Material

Each patient will be provided with a guide with the information explained above. Annex 16.

The diary will also be provided at this meeting in which they have to write down everything related to their daily physical activity, cough, expectoration, dyspnea, medication intake, visits to the emergency room and adherence (Annex 2).

1. Sociedad Española de Neumología y Cirugía Torácica. GEMA 2009: Guía Española para el manejo del asma. Madrid: Luzán 5; 2009.

2. Global Initiative for Asthma. Global Strategy for Asthma Management and Prevention, 2018 [Internet]. [cited Dic 18 2018]. Available from: www.ginasthma.org

3. Calvo Corbella E, Grupo de Respiratorio de Atención Primaria, Sociedad Española de Medicina Rural y Generalista, Sociedad Española de Neumología y Cirugía Torácica, Sociedad Española de Neumología Pediátrica, Associació Asmatològica Catalana. Guía Española para el Manejo del Asma (GEMA) para pacientes, padres y amigos. Barcelona; 2005.

4. Global Initiative for Asthma. Global Strategy for Asthma Management and Prevention. Online Appendix, 2018. [Internet]. Available from: www.ginasthma.com

5. Consenso SEPAR-ALAT sobre terapia inhalada. Archivos de Bronconeumología. 2013;49:2-14.

6. Sociedad Española de Neumología y Cirugía Torácica (SEPAR). Terapia inhalada. Teoría y práctica. [Internet]. [cited Dic 18 2018]. Available from: https://issuu.com/separ/docs/terapia_inhalada._teoria_y_practica

7. Howell JB. The hyperventilation syndrome: a syndrome under threat? Thorax. 1997;52. Suppl. 3:S30-4.

8. Petruson B, Theman K. Reduced nocturnal asthma by improved nasal breathing. Acta Otolaryngol. 116.ª ed. 1996;490-2.

9. Kairaitis K, Garlick SR, Wheatley JR, et al. Route of breathing in patients with asthma. Chest. 1999;116:1646-52.

10. D. Price Q. Zhang V. S. Kocevar D. D. Yin M. Thomas. Effect of a concomitant diagnosis of allergic rhinitis on asthma-related health care use by adults. Clin Exp Allergy. 2005;35:282-7.

11. McIlwaine M. Physiotherapy and airway clearance techniques and devices. Paediatr Respir Rev. 7. Supl 1. 2006;S220-2.

12. Courtney R, Cohen M. Investigating the claims of Konstantin Buteyko MD PhD: the relationship of breath holding time to end tidal CO2 and other proposed measures of dysfunctional breathing. J Altern Complement Med. 14.ª ed. 2008;115-23.

13. Parsons JP, Hallstrand TS, Mastronarde JG, Kaminsky DA, Rundell KW, Hull JH, et al. An Official American Thoracic Society Clinical Practice Guideline: Exercise-induced Bronchoconstriction. American Journal of Respiratory and Critical Care Medicine. 2013;187(9):1016-27.

Appendix 15. Focus group script.

- What does it mean, in your experience, to have asthma?
- What challenges does it present to you daily? What fears does it provoke in you to face your day-to-day life?
- What learning did it mean and does it mean to you?
- Why would you recommend another person to do Nordic walking/participate in this type of study? What positive aspects do you find in the activity?
- What negative aspects do you find in carrying out these interventions?
- What has changed in your way of living with asthma after carrying out this intervention?
- What would you like to be able to change, to have the opportunity to modify the sessions/interventions somewhat?
- What aspects of the design of the interventions do you think should not be modified?
- What other treatments have you tried for asthma apart from conventional medication?

Appendix 16. Patient guide

Using a pressurized cartridge inhaler

Maintenance	Cleaning
Do not expose to temperatures above 50ºC.<br>Keep them protected from direct sunlight.<br>Storage at temperatures below 30ºC.<br>Keep away from fire. Even though it's empty.<br>Perform 2-4 puffs in the air when using it for the first time, as well as if it has been several days since last use.	The cartridge is removed and the casing is washed with water and neutral soap.<br>Rinse with plenty of water and dry well (insist on the area where the valve sits).<br>Reinsert the cartridge.<br>In compact systems, in which the cartridge is not removed, it is preferable to clean the container with a damp or dry cloth, but it is not advisable to disassemble or wet them.

Inhaler use
For pMDI shake before each dose, to ensure dose homogeneity.<br>Remove the cap from the pMDI.<br>Air pulsation if it is the first time it is used or 1-2 weeks have passed since its last use.<br>Place the device between the teeth and seal the lips around the mouthpiece.<br>Exhale to near residual volume.<br>Activate the cartridge only once. Start breathing in later (no more than 3 seconds), slowly to total lung capacity. Slow and deep inspiration: less than 30 litres/minute.<br>Apnea 10 seconds after full inhalation. It increases the permanence of the particles in the lungs, which favors their deposition by sedimentation and diffusion.<br>If two doses are needed, the process is repeated. Pressing twice at the same time reduces the amount of drug that reaches the lungs.

You can find a video in the ASMACONTROL app that will help you remember the correct protocol. Video: MDI.

Using a pressurized cartridge inhaler with a spacer

Spacer chamber
It avoids the need for coordination between inhalation and lung movement.<br>It improves the distance and amount of medicine that reaches the bronchial tree.<br>Decreases the deposit in the oropharyngeal region, cough, candidiasis and the risk of deleterious side effects.<br><u>Cleaning and maintenance:</u><br>Chamber cleaning is required at least once a week. To do this, it must be completely disassembled, washed with warm water and a mild detergent.<br>It is left to dry without rubbing, to avoid the electrostatic charge that would cause the aerosol particles to stick to the walls of the same, reducing the amount of it that reaches the lungs.<br>The valves must be checked before using the inhaler and the chamber will be replaced if they are not in good condition or have cracks.

You can find a video in the ASMACONTROL app that will help you remember the correct protocol. Video: MDI+CAM

Using a dry powder inhaler

Maintenance	Cleaning
Store the inhaler in a dry place, protected from moisture.	Clean the device with a lint-free cloth or dry paper around the mouthpiece after use.<br>Close the inhaler afterwards and store it.
Correct use of the inhaler	
Remove the cap from the mouth piece.<br>Open the DPI, insert the capsule immediately, and close it.<br>Press the button located at the base of the DPI to pierce the capsule.<br>Empty the lungs by avoiding exhalation into the mouthpiece after piercing the capsule.Colocar la pieza bucal en la boca. Labios cerrados alrededor y evitar que la lengua obstruya.	

Inhale from the beginning with the maximum inspiratory effort, as strong and deep as you can, holding it as long as possible.<br>Stop inhaling only after you have completely filled your lungs.<br>Remove the DPI from the mouth without exhaling into it.<br>Hold your breath for 8-10 seconds when inhalation is complete

You can see an explanatory video in ASMACONTROL searching for the name of the one you use. If it does not appear, you can be guided by the videos that are there, since they are similar

Nebulization

Correct use of nebulization
A slow breathing pattern is recommended<br>Tidal volume.<br>Deep breaths are interspersed.<br>Avoid hyperventilation.

Tips for a fully healthy life

General recommendations:
Have all mandatory vaccinations up to date.<br>Get vaccinated against the flu. The flu can contribute to asthma exacerbations, although you have to know that getting vaccinated will not reduce them. Just prevent them from appearing to a greater extent.<br>Quit smoking and avoid environments with tobacco smoke.<br>Doing physical activity. Important for general health. Improves cardiopulmonary health. Regarding exercise-induced bronchoconstriction: inhalation of a short-acting beta-agonist (SABA) is recommended 15 minutes before exercise.<br>Healthy diet, rich in fruit and vegetables.<br>Reduce weight in case of overweight or obesity.<br>Avoid the use of polluting heating and cooking sources, if unavoidable, outdoors as far as possible.<br>Avoid environmental pollution.

Coping with emotional stress.<br>Avoid exercising and spending long periods of time outdoors, under unfavorable weather conditions: very cold temperatures, low humidity or significant air pollution.<br>Use anti-mite covers on the mattress (especially in cases of allergic asthma).<br>Use acaricides (especially allergic asthma).<br>In case of allergic asthma, it is also recommended to reduce exposure to the allergen.

If you have any questions, you can contact the research team by writing us an SMS or Whatsapp, calling or sending us an email through the following means:

Phone Number: +34 628 10 10 24 *e-mail: mn.udc2019@gmail.com*

Appendix 17. Training program

Warm-up

Joint mobility

Ankle: 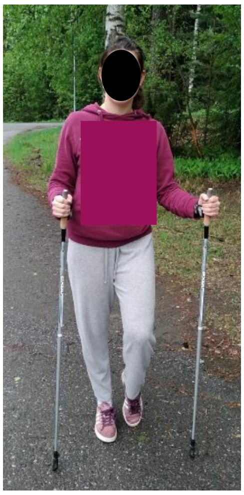	Knee: 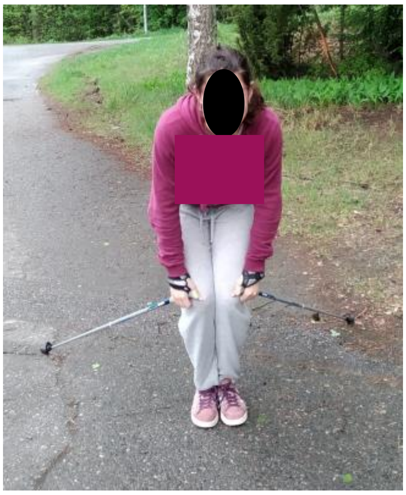	10 x pierna x sentido
Hip 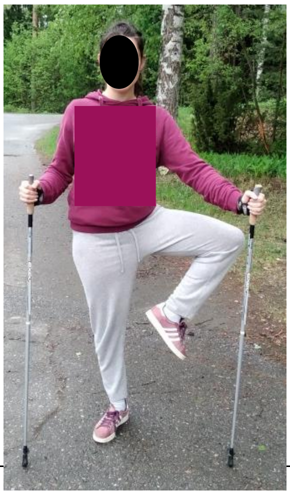	Hip 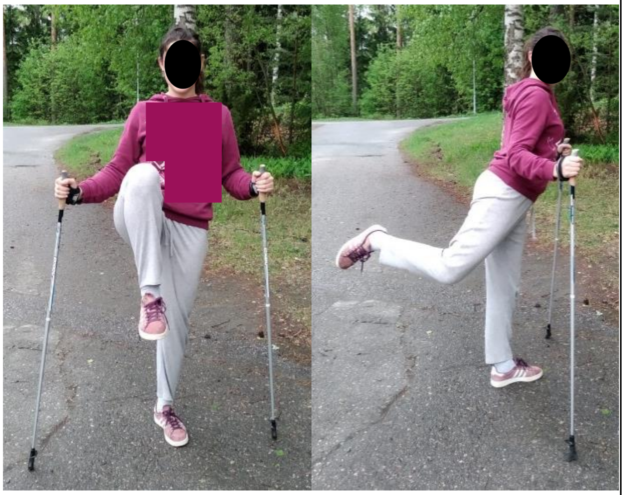	

Upper limbs joint mobility with rods	
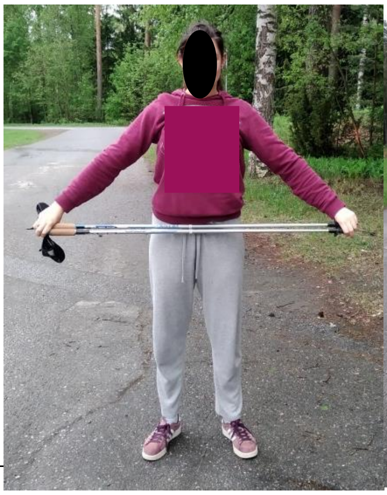 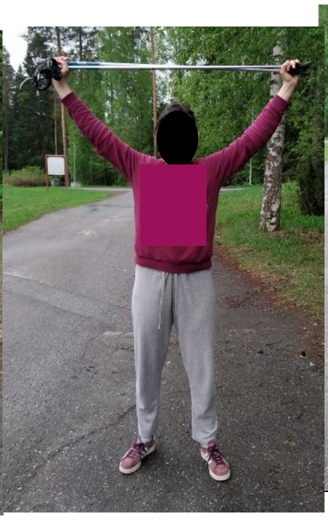 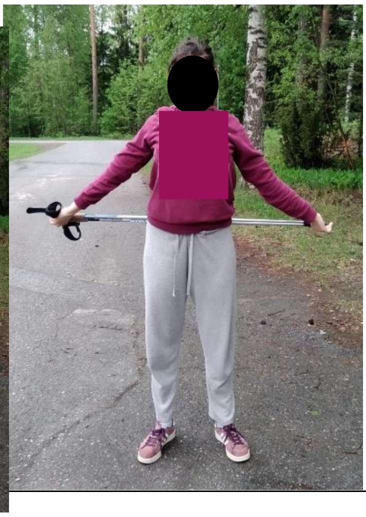 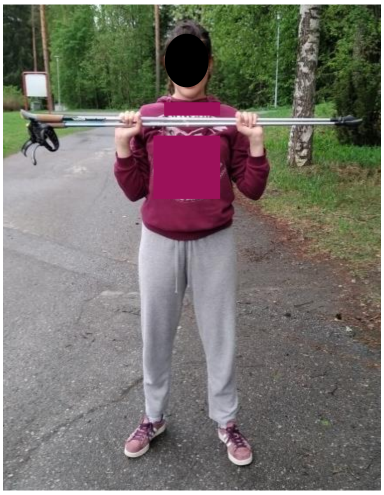 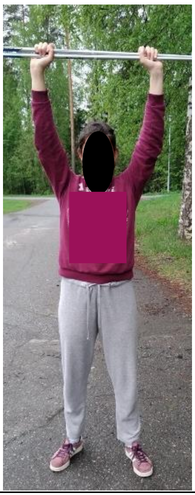	X 10

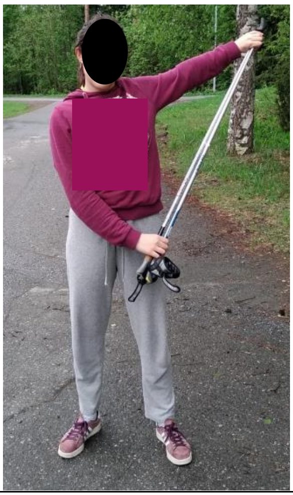	

Poles on the ground in front of the body. Feet set wider than the poles. A squat is performed at the same time that the MMSS perform an oblique ABD that ends with both arms parallel to each other. That is, at the same time that the squat is performed, the arms make an imaginary circle that closes in front: 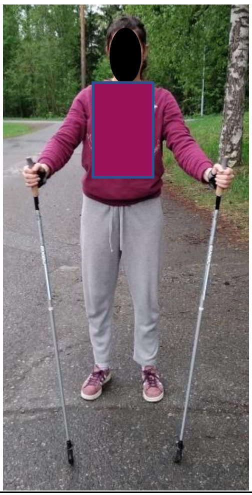 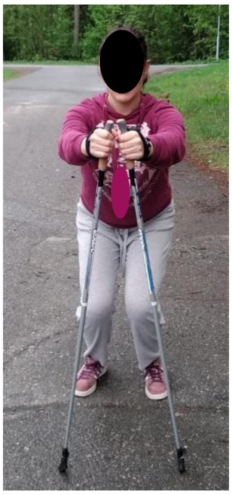	x 10

Poles on the ground in front of the body, about the same width as the feet. A "half squat" (shorter path) is performed at the same time as a glenohumeral external rotation movement is performed, so the arms open:<br>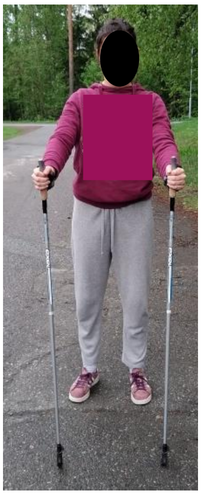 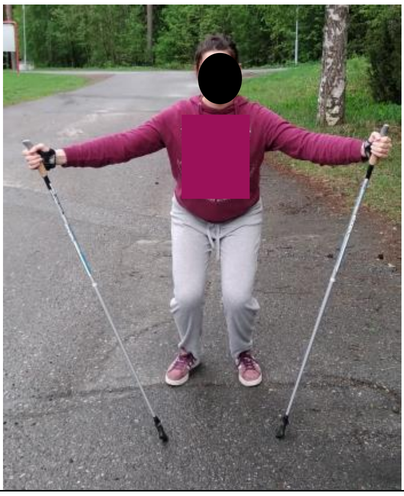	x 10
"Fighting with sticks" Semi flexion of the knee and holding the poles in front of the body, the arms move alternately, coordinated and quickly making a "punching" movement".<br>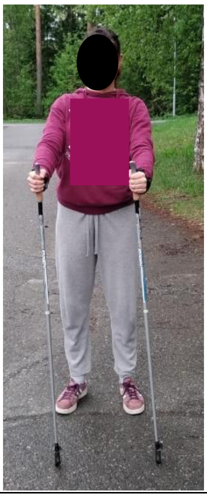	x 10

With the poles advanced, we make strides on the spot.<br>.<br>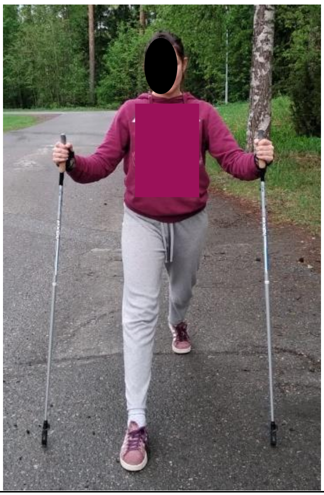 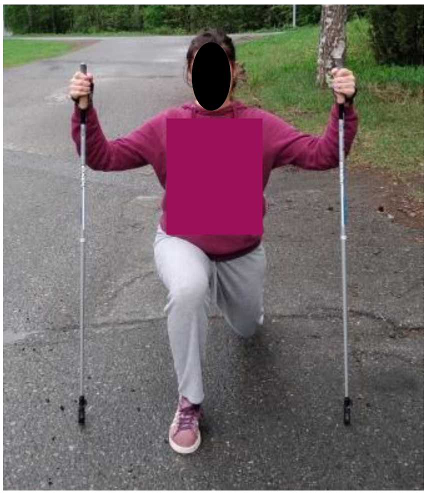	x 10

Jumps on the spot with the poles on the ground	x 10

Walk at a normal pace for 5 minutes and with the poles	x 10

Main part

Participants will perform 30 minutes of continuous Nordic walking.

Initially, work is done at 50% of the theoretical HRmax, and it will gradually increase, 5% per week, until reaching the maximum HR of work, which will be 75% of the theoretical HRmax.

Participants will be instructed to work between a 4 and 6 of dyspnea and fatigue of lower limbs in the Modified Borg Scale.

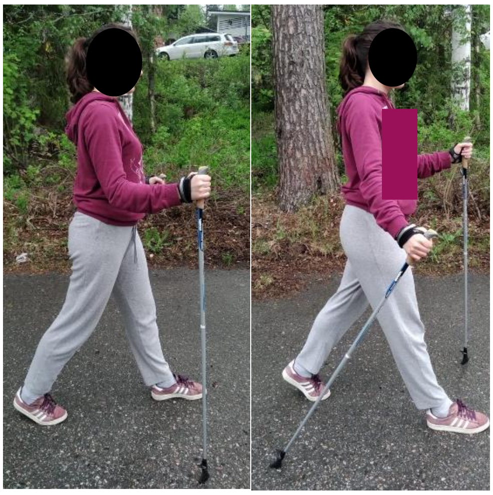

Back to the calm

Stretching

Stretching of the main muscles involved in walking will be performed. Triceps surae, quadriceps, hamstrings, glutes, piriformis, latissimus dorsi, pectoralis, triceps will be stretched.

Triceps surae
Quadriceps
hamstrings
Buttocks
Pyramidal

Latissimus dorsi
Pectoral
Triceps

Relaxation

We will do 5 minutes of relaxation to end the session. Seated patients (if there are benches or walls of adequate height, otherwise sitting on the floor), they will be aware of their breathing for 5 minutes. They will focus on different sensations sequentially. From the sensation of breathing in your nose, how the air goes in and out, to noticing how the lungs inflate and deflate just like your belly. Instruct that they should divert their thinking towards those sensations whenever it strays into other topics or feelings.

Summary table

Heating	Main part	Back to calm
Joint mobility:<br>Ankles.<br>Knees.<br>Hip.<br>Shoulders.<br>Squat + circle<br>Half squat + RE<br>punches in the air<br>Stride<br>jumps<br>walk at a slow pace	Walking at defined HR	Stretching<br>• Triceps surae.<br>• Hamstrings.<br>• Quadriceps.<br>• Buttocks.<br>• Pyramidal.<br>• Wide dorsal.<br>• Chest.<br>• Triceps.<br>Relaxation
